# Experimental versus clinical studies capture distinct types of relapse in tuberculosis

**DOI:** 10.1128/mbio.01977-26

**Published:** 2026-09-18

**Authors:** Christian T. Michael, Maral Budak, Philana Ling Lin, Denise Kirschner

**Affiliations:** 1 Kirschner lab, Department of Microbiology & Immunology, University of Michigan - Michigan Medicine 12266, Ann Arbor, Michigan, USA; 2 Department of Pediatrics, UPMC Children’s Hospital of Pittsburgh, University of Pittsburgh School of Medicine 12317, Pittsburgh, Pennsylvania, USA; Universiteit Gent, Gent, Belgium

**Keywords:** multi-scale modeling, paucibacillary tuberculosis, diagnostic sensitivity

## Abstract

Tuberculosis (TB) remains a global health concern, as *Mycobacterium tuberculosis* (Mtb) currently infects a quarter of the world’s population. Though many TB patients cure infection with short-course treatment, regimens shorter than 4 months risk post-treatment relapse. This is facilitated by the presence of granulomas, spatially heterogeneous immune structures that form during infection that harbor a caseated core that sequesters Mtb in a non-replicating state and pharmacokinetically impedes antibiotics. Typically, clinical relapse is described as TB recurrence <2 years after a misdiagnosis of cure upon treatment completion (MDxC). Comparatively, many experimental models cannot (or do not) screen for cure and examine relapse ~2 months after treatment completion. Understanding how relapse occurs, and whether it is impacted by experimental versus clinical study designs, may improve treatment. Here, we examine two hypothesized mechanisms of relapse: (i) reservoir, where treatment kills all Mtb except those in a non-replicating, diagnostically inaccessible host reservoir; and (ii) threshold, where replicating Mtb remain alive below detectable levels within a test-accessible reservoir. We use our computational model capturing whole-host Mtb infection dynamics, HostSim. Our simulations uncover rates of reported relapse correlating with how studies are performed. Experimentally, relapse is likely driven by incomplete sterilization of replicating bacteria, whereas clinical relapse is most likely driven by gradual expansion of non-replicating Mtb from caseum into granuloma cellular areas. This suggests that TB patients who relapse may best be re-treated with regimens including caseum-penetrating antibiotics, such as rifamycin-class antibiotics, and that experimental animal relapse models better translate to clinical when “cure” is established at treatment completion.

**IMPORTANCE:**

Incomplete treatment of TB leads to risks relapse, which may occur years later. The threat of relapse is the main reason TB treatment takes 4–9 months. Predictors of relapse are not well-defined given variability in technical definitions of relapse and nuances of study designs. Here, we use biologically based computation to simulate both clinical and experimental TB relapse studies, employing multiple diagnostic tests and relapse definitions. We find that two hypothesized types of relapse, each possibly requiring different re-treatment strategies, are simultaneously at play. Simulations suggest that relapse after a “cure” diagnosis (most often clinical studies) is caused by reactivation of non-replicating bacteria hidden from treatment within necrotic granuloma tissue or other sites, whereas experimental systems that cannot or do not test for cure post-treatment are likely to report relapse caused by incomplete sterilization of replicating Mtb. Predictions also depend on the currently unclear relationship between bacterial burden and clinical symptoms.

## INTRODUCTION

Pulmonary tuberculosis (TB) remains a dire concern for countries across the globe and requires months of intensive treatment. A staggering one-quarter of the human population is infected with *Mycobacterium tuberculosis* (Mtb) (1), with over 10,000 cases provisionally reported in the United States in 2025 (2). Approximately 90% of those infected individuals harbor clinically latent TB infection (LTBI), while the remainder progress to clinically active TB disease (3–6). Accurate diagnosis and successful treatment of Mtb infection are required to eradicate TB disease worldwide, as incomplete treatment and poor compliance have led to a rise in drug-resistant Mtb strains (7). The World Health Organization (WHO)—and Centers for Disease Control and Prevention (CDC)—recommended treatments for drug-susceptible, active TB are between 4–9 months using multiple drugs (8, 9), and the WHO-recommended treatment for latent Mtb infection takes 3–6 months (10) often with many side effects. The WHO has identified a need for shorter treatment regimens to improve adherence and lower health costs (11).

Many patients likely only need three months of treatment or less, though shortening the recommended treatment duration is dangerous as we cannot predict who will relapse (12). Relapse (or recurrent TB disease consequent to effects of incomplete antibiotic sterilization) poses a risk to patient health and promotes evolution of drug-resistant Mtb strains (13–15). Standard treatment with HRZE—a first-line regimen of care for treating drug-susceptible TB, consisting of isoniazid (H), rifampicin (R), pyrazinamide (Z), and ethambutol (E) (9)—results in ~5% relapse rate with 6 months of treatment or ~a 20% rate for shorter-course treatment (16). Still, that more than half of TB patients are successfully treated within 2–3 months (12) presents a critical balancing act for public health agencies and clinicians alike as shortening treatment has the potential to reduce the risk of drug-induced side effects, financial and human public health resources.

Granulomas, the site of pulmonary Mtb infection and hallmark of human TB, pose a central challenge to studying relapse. These complex lesions form during Mtb infection and they provide infection-site-specific pharmacokinetic and pharmacodynamic (PK/PD) barriers to treatment. They primarily form within the lungs and mediastinal lymph nodes in humans (17–20). Necrotic cores found in most granulomas (caseum) can harbor non-replicating Mtb, which may be a source of delayed relapse. Note that we refer to lung granulomas unless otherwise specified, though granulomas commonly form in thoracic lymph nodes (21), likely impacting adaptive immune priming (22, 23); granulomas may also form during extrapulmonary TB—e.g., liver (24) (see File SA1 for more on granulomas) (17, 25).

**Fig 1:**
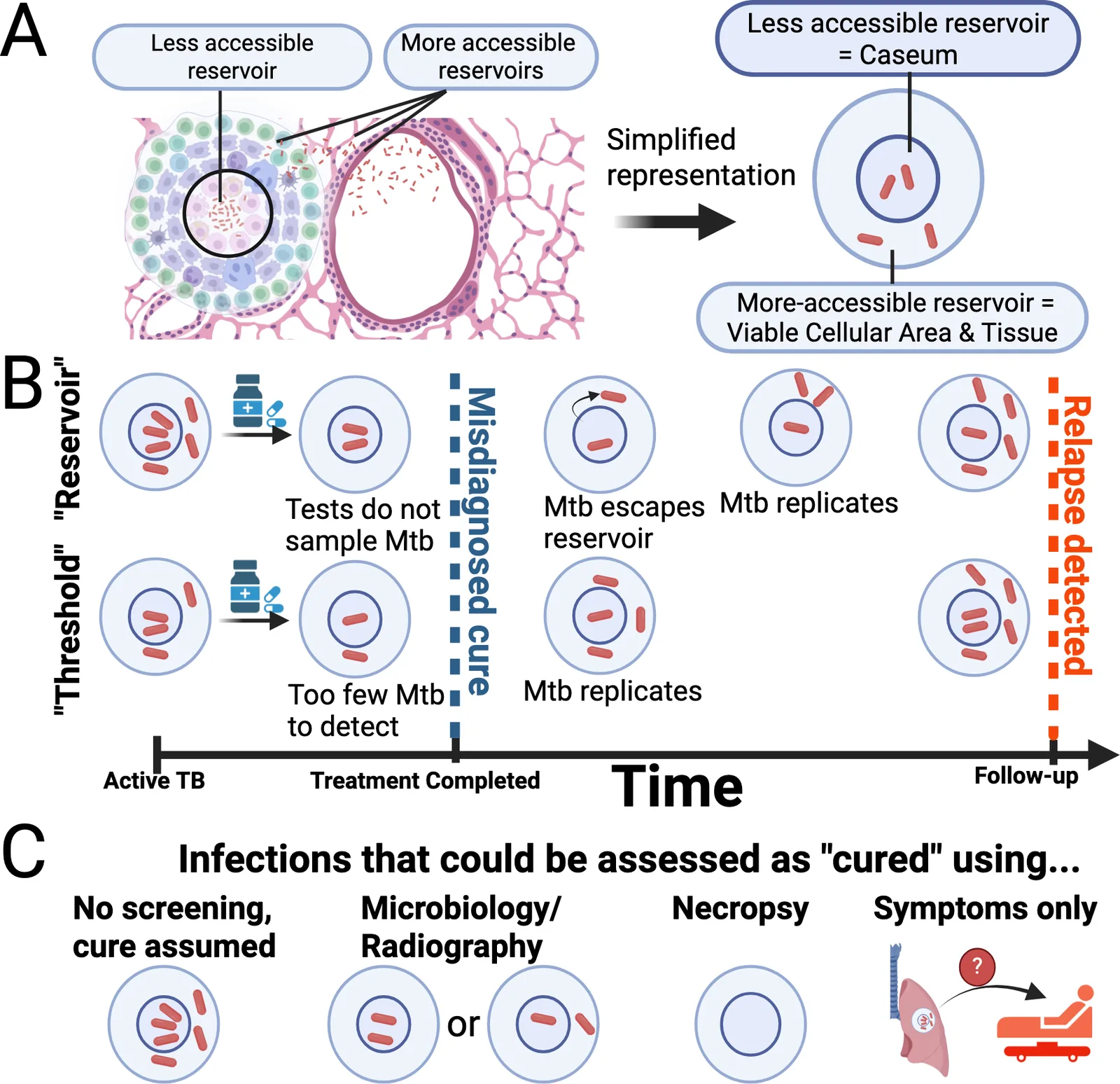
Reservoir- and threshold-driven TB relapse. We represent the two different means by which relapse can result from misdiagnosed cure (MDxC) during cure screening at the completion of a TB treatment regimen. (**A**) This framing of relapse dichotimizes TB infection into two reservoirs (although both could be present): a less-accessible reservoir and a more-accessible reservoir of Mtb. A simplification we make through this work is to assume that the less-accessible reservoir is caseous necrosis within granulomas, though less-accessible reservoirs may vary between species—e.g., most relapse mouse models (RMMs) do not form caseum necrosis. (**B**) Reservoir-based relapse is the event that occurs when a host is MDxC because all surviving Mtb are in a reservoir less accessible to diagnostic tests. By contrast, threshold-based relapse occurs when the diagnostic test is capable of sampling Mtb from still-infected reservoirs, but there are too few Mtb bacteria there to detect. In either case, the surviving Mtb bacilli eventually replicate and result in a TB-positive test result at a follow-up time. (**C**) Rates of relapse reported by a given study may be affected by the diagnostic test used to establish MDxC. Some animals like RMMs cannot be resampled, and thus cure must be assumed at treatment completion. Other experimental approaches use microbiology, radiography, symptoms, or necropsy to probe the level of Mtb either at initial diagnosis, treatment completion, or follow-up. It is not clear how the location and metabolic activity of Mtb lead to symptoms, sometimes used to assess relapse. (Created in BioRender [C. Michael, 2026, https://BioRender.com/7q7afva.])

Though disease relapse is consequent to incomplete Mtb sterilization during treatment, the precise sequence of events within granulomas leading to clinical relapse is unknown. A recent review (12) theorized two mutually compatible mechanisms that may lead to relapse: reservoir and threshold (Fig. 1). “Reservoir” suggests a reservoir of non-replicating Mtb are all that remain after TB treatment, which then later evolve the host into a subclinical state of TB (14); and “threshold” implies there are a few replicating Mtb that are below a level of test detection (LOD) that remain after TB treatment or reside in a test-inaccessible compartment until treatment stops and subsequently, Mtb repopulate. (We note that the reservoir mechanism originally hypothesized in reference 12 was originally phrased as “persistence relapse,” but we adopt the terminology “reservoir-driven” to disambiguate from “persister Mtb”; the latter denotes Mtb that survives antibiotic treatment [26] and could be used to describe Mtb in either reservoir- or threshold-driven relapse.) In the case of threshold-driven relapse, non-replicating Mtb likely still remains in reservoirs like caseum, but their role in TB recurrence is likely overshadowed by surviving Mtb that remains in their replicative metabolic state. Knowing which is at play may inform recommended re-treatment during relapse and also advise experimental studies on better ways to translate predictions from animals to humans. These mechanisms are challenging to separate both experimentally and clinically (12), so it is currently not known what drives relapse.

There are relatively few high-resolution relapse data sets because (i) studies aimed at shortening treatment in humans are designed to consider an increased associated relapse risk (12), and (ii) animal studies face financial and translational challenges. Clinical trials are the gold standard for analyzing treatment regimens and their relapse potential, but can take years to complete and cost an average of $40M (27). These are often longer-course treatments designed to minimize relapse likelihood; thus, relapses appear as rare events in human data sets by design. Multiple *in vivo* animal studies are employed to study TB outcomes (20), including relapse—most notably including expensive and low sample size nonhuman primates (NHPs) (28–30) and a variety of relapse mouse models (RMMs) (31–34), each with advantages and disadvantages.

It is not currently known how differences in relapse study designs between human and experimental animal studies impact translatability of conclusions. To our knowledge, studies addressing relapse assume (often by necessity) that relapse is principally driven by a uniform set of mechanisms across all study designs. Differences between human and animal studies may be in part due to either study design, how relapse is measured, or underlying physiological differences. Currently, precise relapse measurements are not directly compared between humans and animal studies. We bridge this gap by using a whole-host scale quantitative systems pharmacology computational model to study TB treatment, called HostSim.

### TB relapse in humans

We summarize the clinical presentation of active TB and relapse to clarify terminology. Clinically, active TB is associated with signs (e.g., cavitary lesions on chest X-rays, fever, etc.) and symptoms (cough, fatigue) of TB, usually with microbiologic confirmation of Mtb infection (sputum). During treatment, signs and symptoms often improve (improved chest X-ray, sputum smear, and culture are Mtb-negative, resolution of cough and weight loss). Subclinical TB is another form of TB disease that is clinically asymptomatic but has diagnostic markers associated with active TB, such as Mtb present in sputum or bronchoalveolar lavage fluid (BALF), unlike most LTBI cases (4). Clinically, relapse refers to having new signs and symptoms of TB after resolution by the end of treatment and after exogenous Mtb re-infection has been ruled out (12) (though in practice, ruling out reinfection is challenging (35).

Clinical relapse is typically defined as occurring after a patient has been misdiagnosed as cured (MDxC, Fig. 1). We use the phrasing of MDxC rather than “tested as false-negative” to avoid confusion between “Mtb-negative” versus “cure-negative.” Occasionally, relapse is grouped with treatment failure under the primary endpoints of “unfavorable outcomes” in studies with few reported instances of TB relapse (36, 37). The rate of relapse is typically very low in trials (<5% in most cases), as clinical trials prioritize patient safety and may be canceled if the risk of relapse is unacceptably high (12, 38). It is impossible to eliminate MDxC cases, as to date there are no studies that can prove sterilization from all sites of infection. That is, there is no test that can determine whether a patient is completely sterile.

While the goal of treatment is always sterilization of Mtb from all sites (lungs, lymph nodes, extrapulmonary sites may have varying burdens), resolution of symptoms and/or negative sputum cultures are typically used as a surrogate for assessing true sterilization. Patients treated for TB include those who are considered “TB-cured” (e.g., sputum culture negative after treatment is completed) and “TB-treated” (meaning they complete treatment but did not necessarily confirm that sputum was Mtb-negative). The additional step of cure-screening with a bacteriological confirmation of cure is not always performed, and it is common to conflate “TB-cured” with “TB-treated” as equivalent stand-ins for sterilization. However, when relapse clinically follows MDxC, it may be that the most influential mechanisms driving relapse (and optimal re-treatment) are different between “TB-cured” and “TB-treated” patients; or may differ by clinical and microbiologic measures used to assess patients for cure. For this reason, we delineate between “cure-screened relapse” (typically clinical studies) to indicate relapse after an Mtb-infected host has relapsed subsequent to a microbiologic test for Mtb negativity, and “unscreened relapse” (typically experimental studies) as those relapse events that follow from treatment completion without any microbiologic test at the time of treatment completion.

Relapse assessment is not uniformly performed, varying based on test availability, operational definition, and consistency of medical care. Relapse is typically reported using a composite of one or more measures including positive bacterial smears or cultures, rapid-test molecular analysis, radiographic exams, and/or clinical TB symptom presentation (12, 29, 36, 37, 39). Confounding things, each test may potentially identify different patients as having relapsed if tests used have different levels of detection (LODs). Some tests are commonly used to determine cure after treatment (e.g., two consecutive Mtb-negative sputum smears [40]), though no tests to date have been validated to predict relapse. Though specific tests can vary, some form of cure-screening test is performed in most human studies that we re-examine here, except where noted. We find that many studies report relapse only as a recurrence of symptomatic active TB disease, possibly with bacteriological follow-up (16, 41–44), though detection of non-symptomatic infection after treatment is sometimes reported or discussed as relapse (37). Table SA1 presents a list of diagnostic tests used to assess TB diagnosis, cure, and relapse (based in part on reference 12).

Although relapse can often be based on symptom presentation, the connection between patient symptoms, diagnostic inflammatory markers, and lung bacterial burden remains murky in both human and animal model TB. Even if one knew the precise number of Mtb and their metabolic state at every timepoint within lungs, we may not know whether culturable Mtb or symptomatic relapse will result. Patients with subclinical TB remain asymptomatic while shedding viable Mtb, which was historically considered a hallmark feature of clinical TB (4, 45). (This phenomenon is observed in NHPs, referred to as percolators, and comprises ~5% of macaques [46–48].) Previous studies found 10-25% of LTBI patients exhibited neutrophilic whole-blood transcriptomic signatures consistent with active TB (4, 49). In tandem with this, we observed in simulated virtual patients that inflammation markers, such as interferon gamma (IFN-γ; often used to diagnose TB; see File SA), do not predict host outcomes (50). Conversely, clinical studies have shown multiple inflammatory markers are present in patients with LTBI (51). Together, this suggests that not all asymptomatic cases are equal (4), with some engaging in an ongoing immune battles likely to contain metabolically active Mtb from escaping granulomas, while other cases involve surveillance over non-replicating, caseum-trapped Mtb. This creates a spectrum of inflammatory signatures and symptoms that suggest more than one modality of relapse is possible.

### Experimental animal models of TB relapse

Both RMMs and NHPs, the most common animal relapse models, provide detailed relapse data sets but bear some substantial differences from each other and from human clinical relapse studies. RMMs can be readily treated with human-equivalent doses (i.e., corresponding average plasma concentrations (52, 53) of first and second-line antibiotics. However, most mice do not form necrotic granulomas with the exception of C3HeB/FeJ mice and the ultra-low dose model (34, 54–56), and mice have disease states incongruent from those in humans (for example, mice experience chronic not latent Mtb infection). NHPs most closely represent human TB, forming lung granulomas in human-like caseum, but studying NHPs requires expensive resources and expertise; however, sample sizes can be small (57). Animal models have been given (intentionally) weak antibiotic regimens to allow for reliable and experimentally controllable relapse (29), though this experimental formulation of relapse may capture a disease state with large (if potentially asymptomatic) bacterial burdens at treatment completion.

In many cases, it is necessary to use proxy measures in lieu of host state when studying relapse in experimental animal models, and it is unclear whether these proxies over/underrepresent different mechanisms driving relapse. Assessing cure in animal models is not always comparable to diagnostic tests used in humans (e.g., repeated sampling of CFU in RMMs is not consistently possible (58), or there is species-specific disease presentation (59); thus, individual animals are often not screened for cure when studying relapse (Fig. 1) and use alternative measures. For instance, CFU enumeration in experimental mouse lung homogenates or NHP granulomas is used as one proxy for clinical relapse (Table SA1). It should be noted that some of these tests may be more accurate than measures used in humans—e.g., culturing tissue homogenates can capture both replicating and non-replicating Mtb, though such cultures distinguish between them. It is also unclear whether human sputum CFU measures capture similar outcomes.

### Assessing translatability of experimental animal models with human clinical outcomes

If both reservoir- and threshold-driven relapse can occur *in vivo* as hypothesized (12), it is unclear whether both differ in terms of optimal re-treatment, or whether they are uniformly captured in different study designs. While each measurement and proxy has advantages and disadvantages (12) and Table SA1, none can categorically predict relapse. For example, it was found experimentally that in NHPs, measuring IFN-γ in BALF was insufficient for assessing effective antibiotic treatment efficacy (60). Similarly, sputum culture conversion at 8 weeks is a common endpoint for human clinical trials but is not necessarily a good indicator of sterilizing activity (61–63).

To understand how relapse measures and study designs impact translatability and conclusions of relapse studies, we recreate multiple *in vivo* relapse studies using a computational model of relapse. For this, we extend our virtual host treatment model of pulmonary TB, called HostSim*,* to capture mechanisms that may lead to either reservoir- or threshold-driven relapse, as well as perform virtual diagnostic tests that mimic *in vivo* diagnostic tests. HostSim bridges the translational gap between animal and human relapse studies by reproducing both clinical and experimental relapse studies and comparing conclusions. Our simulations suggest relapse that is observed in the clinic after a MDxC is less frequent and primarily reservoir-driven relapse*—*i.e., driven by a slow, continuous release of non-replicating bacteria derived from caseum. This can take a longer time before Mtb levels exceed LOD, potentially explaining why some humans take up to 2 years to relapse (12, 64, 65). On the other hand, simulating relapse as done in most experimental studies without cure-screening upon treatment completion more often results from a reservoir of replicating bacteria, leading to rapid threshold-driven relapse. Though this trend is true regardless of LOD, the relative frequency of these two relapse types depends on the LOD of diagnostic tests used.

## RESULTS

### Simulating misdiagnosed cure of antibiotic TB treatment (MDxC)

To simulate relapse, we study treatment of virtual hosts using HostSim to capture a plausible spectrum of infection and treatment outcomes observed in both humans and animal models. This includes capturing Mtb trajectories before and during treatment, relative rates of reported MDxC, and subsequent Mtb recurrence.

#### HostSim simulates Mtb-immune interactions within heterogeneous primate-like virtual hosts

To mechanistically simulate host outcomes during Mtb infection and treatment, we use HostSim, our recently published, calibrated, and validated whole-host pulmonary TB model (62, 66, 67). Briefly, HostSim is a hybrid agent-based and ordinary differential equation model that represents interactions between host-immune cell populations within lungs, uninfected lung-draining lymph nodes, and a blood compartment. We can also track different Mtb subpopulations within multiple lung granulomas of a single virtual host (Mtb residing within caseum, macrophages, or tissue). HostSim allows us to simulate temporal trajectories of various T cell and macrophage population sizes, as well as subpopulations of replicating and non-replicating Mtb within each granuloma for each host (see Materials and Methods) and their total counts within lungs. Recently, we calibrated HostSim to compare multiple measurements of drug efficacy of various first- and second-line antibiotic regimens by predicting the time-course trajectory of Mtb over time during treatment of drug-susceptible infection (62).

We simulate a heterogeneous set of virtual hosts (our “virtual cohort”) that we treat, recapitulating each treatment and relapse scenario from clinical and experimental studies. For all analyses, we generate a single virtual cohort of 500 Mtb-infected hosts, each patient is inoculated with 13 bacteria leading to 13 granulomas at the start at time = 0 (62). We allow each virtual patient to develop naturally a mature Mtb infection until 300 days p.i., then take a snapshot of each granuloma simulation. From those snapshots, we can use our virtual cohort to study multiple what-if scenarios where we treat the same exact entire cohort with one antibiotic regimen, halt treatment, then observe regimen-specific host and cohort outcomes both short- and long-term.

**Fig 2:**
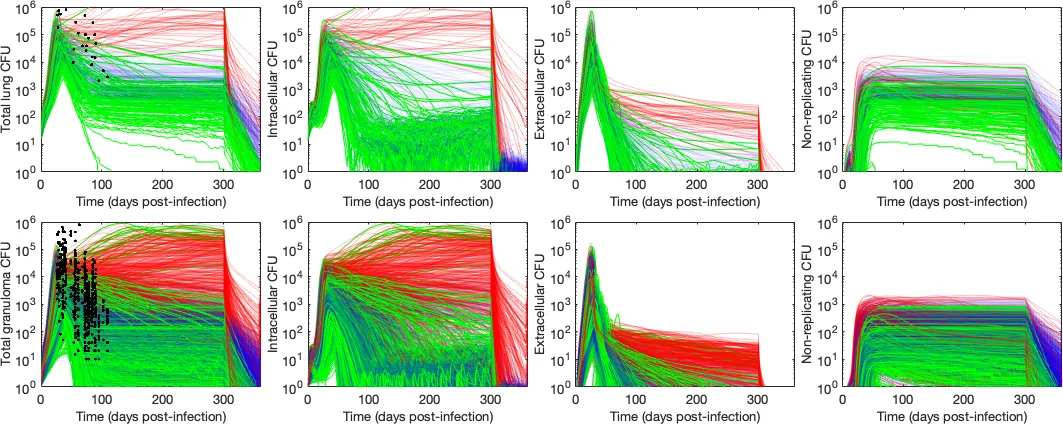
Infection trajectory of virtual hosts and granulomas before and after HRZE treatment. Each panel shows Mtb populations within *n* = 500 hosts (top row) and individual granulomas (13 for each patient, bottom row). All hosts are infected with Mtb at time 0, and, for visibility, we only show CFU from primary granulomas. Treatment begins at day 300 p.i. and ends at day 360 (i.e., after 2 months of treatment). Bacterial trajectories of virtual hosts with active disease prior to treatment (Materials and Methods) are shown in red. Bacterial trajectories of hosts that sterilized with 0 CFU (true cure) by the end of treatment are shown in green, and all other virtual hosts are shown in blue. As in our previous work (62, 66), we compare CFU prior to treatment with analogous published CFU levels in NHP lungs and granulomas (black dots) (68–71).

HostSim simulations capture a spectrum of host outcomes before, during, and after treatment. In Fig. 2, we show dynamics of simulated Mtb bacterial trajectories over time during and after treatment, simulating 8 weeks of HRZE. To simulate relapse, we first capture the spectrum of treatment outcomes: cure (both MDxC and true sterilization) and treatment failure. To assess for cure, we implement virtual diagnostic tests by mimicking those performed *in vivo*, typically by comparing replicating or total Mtb counts against an established LOD (see Materials and Methods).

#### HostSim captures misdiagnosed cure rates reflective of regimen-specific relapse rates

To validate that our model captures MDxC, we simulate multiple short-course (8-week) antibiotic regimens and ensure that the relative MDxC rates reflect relative relapse rates of the well-studied regimens HRZE, RMZE, BPaL, and BPaLM (12, 32, 34, 72–74). We simulate MDxC by subtracting the proportion of virtual hosts or granulomas that truly sterilize (CFU = 0) from those with CFU below a set LOD; this difference isolates hosts that would likely be misdiagnosed as cured, or granulomas that would be assessed as sterile. Although actual relapse studies use a variety of diagnostic tests, we compare true virtual host sterilization against Virtual Mtb plate results, which mimic highly accurate experimental lung and granuloma homogenate cultures capable of detecting non-replicating bacteria (see Materials and Methods and Table SA1). Specifically, we assume that whole-host homogenates have a LOD of 50 CFU, and granuloma-scale LOD is 10 CFU (see Materials and Methods and references 62, 75). To determine whether pre-treatment CFU has an impact on MDxC rates, we delineate between hosts and granulomas that are high/low CFU prior to treatment, as in our previous work (62). For this, we say that a host that is initially high-CFU (total >10,000 CFU pre-treatment) and initially low-CFU (total <10,000 CFU pre-treatment), and a granuloma that is initially high-CFU (>1,000 CFU pre-treatment) and initially low-CFU (<1,000 CFU pre-treatment). It should be noted that high CFU hosts may also have some low CFU granulomas but must have at least one high-CFU granuloma.

Our simulations show regimen-dependent MDxC rates that reflect the relative risk of relapse in other experimental and clinical studies. Our initial observations are not surprising in that most short-course mono-treatments for initially low-CFU hosts result in large rates of MDxC and have little impact on initially high-CFU hosts. Notably, HRZE has a high MDxC rate, particularly in high-CFU patients (42% sterile vs 76% cured), when compared with regimens, including moxifloxacin or bedaquiline (e.g., BPaL with 74% sterile, 80% cured, and RMZE with 80% sterile and 96% cured). This suggests that short-course treatment of high CFU-burden patients with only 8 weeks of HRZE will have a higher risk of MDxC, consistent with both (i) the high (~40%) relapse rates found for short-course HRZE treatment in a 2016–2018 clinical study (12, 72–74) and with (ii) short-course mouse studies directly showing higher relapse rates in HRZ and HRZE than in BPaL, BPaLM, or RMZE (32, 34).

**Fig 3:**
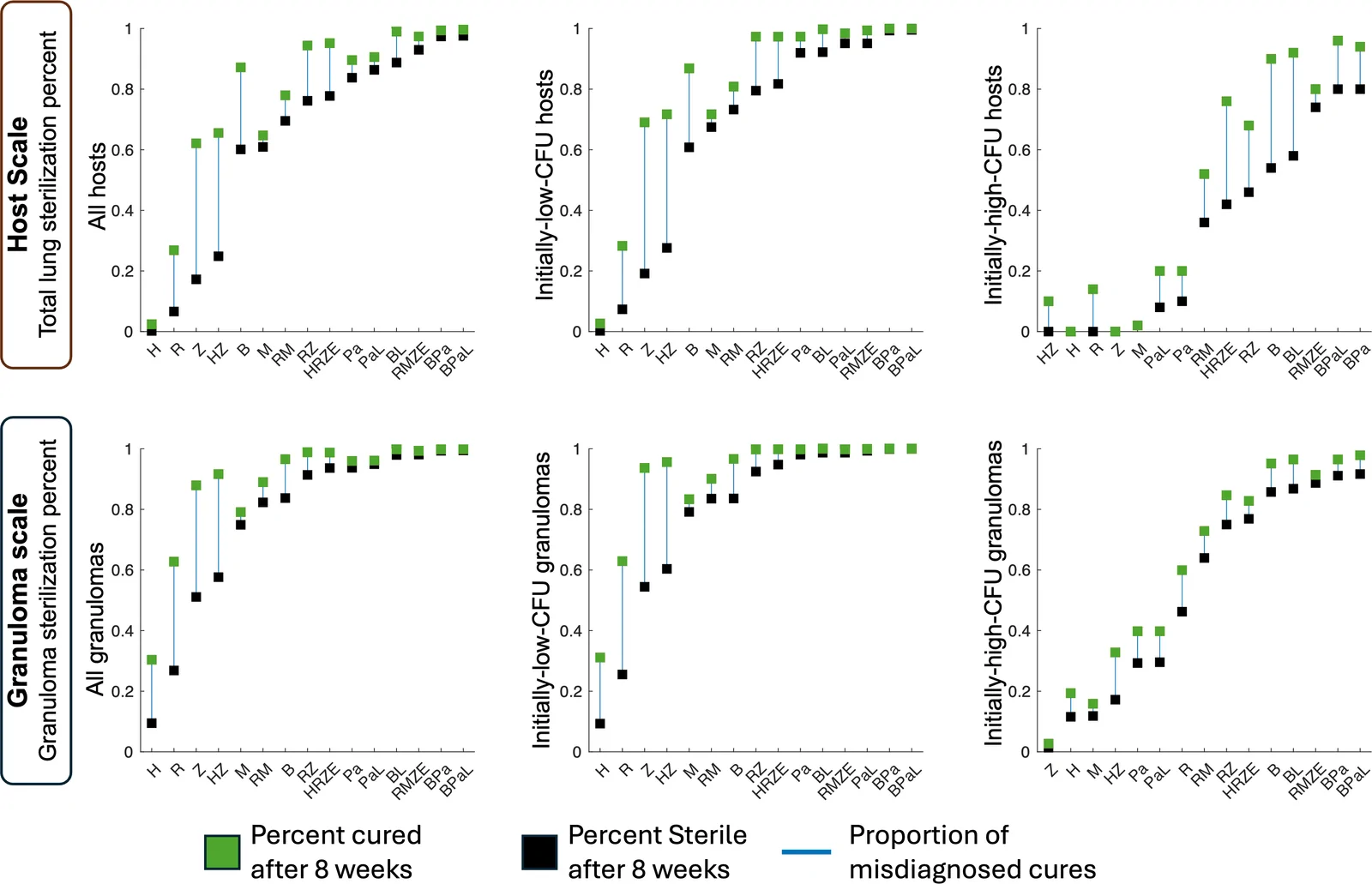
Misdiagnosed cure (MDxC) rates are specific to spatial scale and antibiotic regimen. We show cure and sterilization fractions of virtual hosts and granulomas after short-course antibiotic regimens. We administer a drug regimen for 8 weeks (regimen group i; see Materials and Methods) to each of 500 virtual hosts in a virtual cohort (shown in top row) and a total of 6,500 primary granulomas (shown in the bottom row). For each regimen, the percentages for sterilization (black boxes) and lack of detectable infection (green boxes) after 8 weeks of treatment are shown. The left column shows treatment results for each of these virtual hosts and granulomas, whereas the center (right) columns show results for only hosts and granulomas that were low-CFU (high-CFU) pre-treatment. We notice that higher-CFU granulomas and hosts are more likely to have subclinical infection (i.e., non-sterile but with CFU < 10 [granuloma] or 50 [host]).

Regardless of regimen, both the scale we measure (granuloma or whole host) and virtual pre-treatment CFU levels (high or low CFU) influence MDxC levels. Rates of MDxC are increased for initially high-CFU hosts, as compared with initially low-CFU hosts, particularly after multi-drug regimens (Fig. 3). This positive-control case serves as model validation, reaffirming that hosts with severe infection are those most likely to experience cure-screened relapse. This pattern holds at both granuloma and whole-host scales. Finally, we observe that large granuloma-scale MDxC rates amplify into even larger rates at the host scale, as previously predicted (76).

#### Most cure misdiagnosis stems from incomplete sterilization of caseum

In our simulation studies of virtual hosts with TB, treated for 2 months that experience MDxC after short-course treatment (Fig. 3), we observe that viable Mtb are found within replicative niches only in those virtual hosts/granulomas that had high-CFU prior to treatment, and this was regardless of regimen used. After HRZE treatment, 7/500 virtual hosts have MDxC, and six of those seven are initially high-CFU. Within the MDxC initially low-CFU host, nearly all CFU levels were caseum-bound and non-replicating. One of the six initially high-CFU hosts with MDxC held intracellular Mtb (within macrophages), while others held Mtb within caseum. Pooling HRZE-treated granulomas from all hosts, we observe 13 virtual granulomas had CFU below LOD—two initially low-CFU and eleven initially high-CFU. In both initially low-CFU granulomas with MDxC, all Mtb are trapped within caseum. Of the eleven high-CFU granulomas, eight harbor Mtb solely within caseum while the other three retain Mtb within macrophages at treatment completion. We have the ability to treat the same cohort with different regimens to compare outcomes and after treating the same virtual cohort with a different second-line regimen, BPaL (32, 77), only one initially high-CFU host carries replicating CFU below LOD at treatment completion, with all bacteria held entirely within caseum of an initially low-CFU granuloma at treatment completion. Similar trends followed for other simulated regimens.

Overall, our simulations suggest that relapse studies will likely differentially capture relapse processes—either reservoir- and threshold-driven relapse—that are based on CFU levels of each host prior to treatment. Using HostSim to identify MDxC after short-course treatment (and assuming that the diagnostic test can detect Mtb within caseum), we predict that hosts with low CFU levels prior to treatment likely harbor Mtb trapped within caseum; and, conversely, we predict MDxC hosts harboring replicating Mtb likely had high CFU levels prior to treatment. This suggests that (i) if clinical relapse occurs in initially low-CFU hosts, it will likely be reservoir-driven; and (ii) if a host experiences threshold-driven relapse, it likely had high CFU levels prior to treatment.

### Validating a relapse model by recreating experimental animal relapse studies

With the ability to capture a variety of host states that lead to misdiagnosed cure, we extend and calibrate HostSim during treatment to capture recurrent TB well beyond the time of treatment completion, i.e., to identify if and when relapse occurs. Given that we capture MDxC both within replicative and non-replicative niches, we represent mechanisms that can allow for either threshold- or reservoir-driven relapse. For reservoir-driven relapse, we allow virtual non-replicating Mtb to be stochastically transported out of caseum over time into the cellular region of the granuloma (a role hypothesized to be played by neutrophils [78]), allowing a possibility for caseum-trapped Mtb to become replicative and lead to relapse and/or initiate nascent granuloma foundation (78). Given the efficacy of clinically administered regimens, there are sparse relapse data to calibrate a mechanism for stochastic release of Mtb. However, we assume this mechanism is also responsible for reactivation of LTBI, and so we calibrate rates of caseum-transport by recapitulating reactivation of LTBI during anti-TNF treatment such as etanercept or infliximab (79, 80) (see Materials and Methods). The threshold relapse mechanism is implicitly captured by our model, as Mtb replication is always allowed during or after treatment, and this remains true for numbers of replicating Mtb at the end of treatment (if it is a MDxC).

We define virtual relapse per-experimental study design and classify as either reservoir- or threshold-driven. To prepare each virtual host for relapse assessment, we (i) treat the virtual cohort with different antibiotic regimens, (ii) if specified by the study design, screen hosts for cure upon treatment completion, and (iii) simulate their infection progression without treatment for a specific follow-up period of time. At some future time post-treatment completion, we run a follow-up diagnostic test on each host to determine relapse.

We classify simulations that result in relapse as threshold-driven if there is at least one replicating Mtb at treatment completion, and reservoir-driven otherwise (any remaining Mtb are trapped within caseum).

#### Recreating a radiographic study of relapse in primates

To validate our relapse model, we replicate the design of a recent relapse study performed in NHPs that employed simian immunodeficiency virus (SIV) (29). There, NHPs were treated with short-course antibiotic regimens for active TB only: monkeys (8 weeks of isoniazid and rifampicin, i.e., HR). After a 1-month rest, they were then infected with SIV to induce relapse (5). Relapse was primarily measured via observation of newly seeded granulomas using radiolabeled PET/CT scans; the study reported that 8/12 NHPs relapsed. To replicate this experiment using HostSim, we represent HIV-1 co-infection as initiating a linear decline of CD4^+^ T cells that we calibrated to T-cell blood concentrations reported from the 1990s (81, 82) (and measured prior to any treatment studies, see File SA3).

Standard relapse clinical studies only admit humans with specific pre-treatment disease states (e.g., active TB), so we similarly predict virtual cohort disease states and use them to allow for direct comparison to this study. We classify virtual hosts as having active TB if they have continued active bacterial growth, which we characterize by a species-specific fold increase in CFU between treatment completion and follow-up (Materials and Methods). To ensure results are robust to different characterizations of active TB in our virtual patients, we perform a separate analysis considering the case where virtual hosts maintain high-CFU granulomas (CFU > 1,000 for over a month) as having active TB.

After recreating the conditions of an NHP study (29), our simulations well-reproduce the NHP relapse rates. We examine relapse rates using virtual disease state classifications within five sub-cohorts of our original *n* = 500 virtual hosts (Table 1). As in the original study, we simulate hosts with 2 months of HR treatment and assess MDxC upon treatment completion using a Virtual Clinic score, a virtual diagnostic test we use as a proxy for clinical evaluation based on appearance of new granulomas and increasing Mtb counts (see Materials and Methods). After 4 weeks of drug rest, we simulate virtual HIV infection for a further 8 weeks. At that follow-up time, we assess each virtual host in the full cohort for relapse using a Virtual PET/CT method (see Materials and Methods) to mimic PET/CT-based tests used to determine relapse in the NHP study (29) (Table 1). Using both of our NHP-like active-TB virtual relapse sub-cohorts results in ~50% relapse, close to the 8/12 NHPs from their study data (29).

**TABLE 1:** Predicted radiographic relapse rates by sub-cohort^*b*^

Sub-cohort disease state classification	Does {CFU > 1,000} also mean active?	# relapse out of # treated (% relapsed at 8 weeks)	Proportion of R vs T relapse (no screening), %P | %T	# cure-screened relapse out of # cured in SC (% relapsed at 8 weeks)	Proportion of R vs T relapse (cure-screened), %P | %T
Active (NHP-like)	X	**50/100 (50%)**	16% R | 84% T	20/68 [29%]	40% R | 60% T
Active (NHP-like)	✓	**56/109 (51%)**	20% R | 80% T	24/75 (32%)	46% R | 54% T
Active (human-like)	X	42/47 (89%)	5% R | 95% T	12/15 (80%)	17% R | 83% T
Active (human-like)	✓	52/63 (82%)	13% R | 87% T	20/29 (69%)	35% R | 65% T
All^*a*^ (active +subclinical)	N/A	82/483 (17%)	34% R | 66% T	49/447 (11%)	56% R | 44% T

^*a*^ Note that this this may include treatment of virtual hosts with subclinical or LTBI and may bear closer resemblance to reactivation than clinical relapse.

^*b*^ Bolded cells indicate simulated results that are the most directly comparable to those from a recent NHP relapse experiment (29). Columns 1 and 2 describe how each virtual sub-cohort is defined. Columns 3 and 4 of each row describe the frequency of relapse without cure-screening, and whether these relapses are reservoir-driven (R) or threshold-driven (T). Columns 5 and 6 perform repeat analysis on the same sub-cohort but describe only cure-screened relapse.

**Fig 4:**
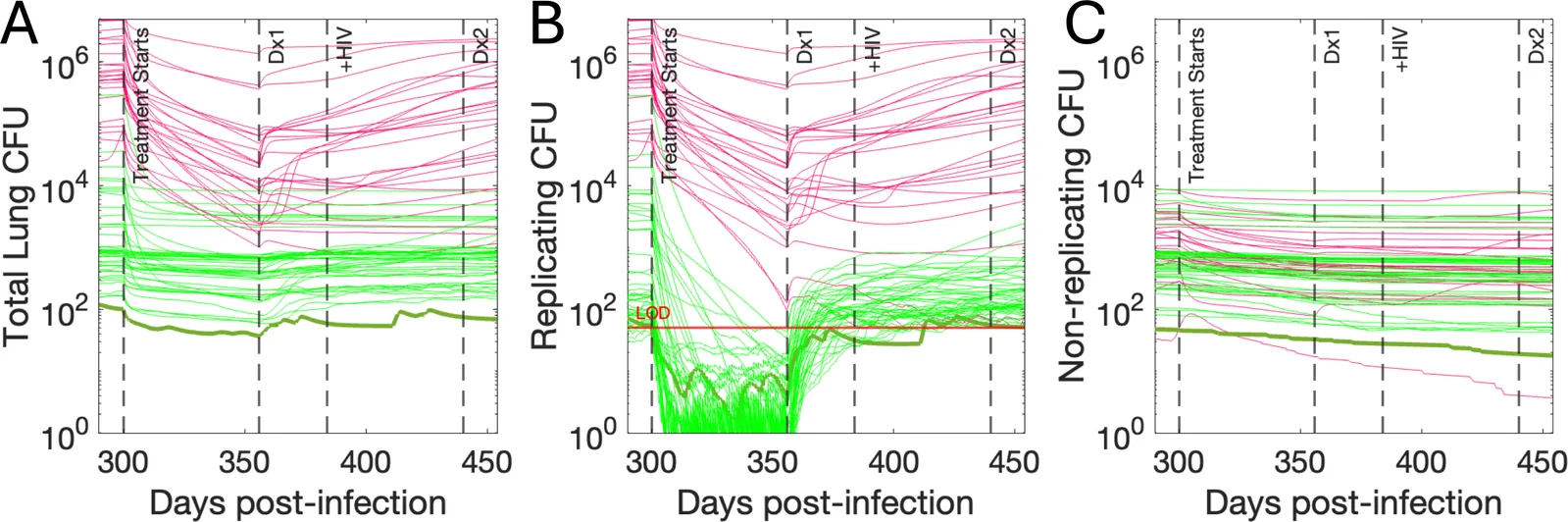
Relapse of virtual hosts after short course of HR and HIV-1 co-infection as in reference 29. Virtual host CFU levels over time for whole-lung total CFU (**A**), replicating (intracellular + replicating extracellular) Mtb only (**B**), and non-replicating Mtb only (**C**). Each line corresponds to a trajectory from one host that was categorized as having active TB prior to receiving HR (using NHP-like classification) then relapsing after the end of treatment. Key time points in days post-infection are shown, indicating the start of treatment, time of cure-treatment (Dx1), onset of HIV, and relapse follow-up time (Dx2). Cure-screened relapses are colored green, while those that would be excluded by cure screening are colored red. The Virtual Clinic score assumes that non-replicating bacteria are not detectable, but the LOD of 50 is shown for replicating CFU. We have highlighted one representative virtual host (dark green) that, despite qualifying as relapse by this study design, may closely resemble subclinical disease or latent TB infection.

We find that the majority of cure-screened relapse appears as reservoir-driven relapse, which mostly exhibits slowly increasing levels of intracellular bacteria (Table 1). Relapse frequency per sub-cohort decreases by 6%–20% when associated with cure screening (as in clinical trials). Simulated relapses occurring after MDxC relapse rates tend to be slower, reservoir-driven (green trajectories in Fig. 4), and feature a low (stochastically oscillating) level of Mtb as bacteria move between niches too fine-grained for experimental detection (50, 83). More than half of simulated relapses subsequent to treatment failure (i.e., those that would have been excluded via cure screening) result from incomplete sterilization of macrophages (Fig. 4, red curves), indicating threshold-driven relapse. Many of these threshold-driven relapses occurred with Mtb still harbored within caseum (red lines in Fig. 4C). Many threshold-driven relapses result in rapid bacterial regrowth in 1–2-week periods that restore CFU to pre-treatment levels, while others exhibit either slow or no regrowth of Mtb populations.

#### Relapse rates from caseum-forming experimental animal models most closely match simulated rates

Since our virtual relapse studies reproduce the experimental NHP relapse study, we survey similarities between HostSim and other relapse models by simulating several experimental animal RMM relapse studies. We chose these studies principally by whether sufficient details were included to perform analogous simulations, and the RMM studies tested many more regimens than those that we recreate here. For applicable comparison, we compare *in vivo* relapse rates to the frequency of relapse in virtual hosts with NHP-like and mouse-like active-TB (see Materials and Methods). The NHP experimental study that we replicate uses the same simulations that we describe in ”HostSim simulates Mtb-immune interactions within heterogeneous primate-like virtual hosts.” To replicate experimental RMM studies, we allow the full virtual cohort of *n* = 500 to develop mature Mtb infection. We then administer a comparative antibiotic regimen, simulate for three months follow-up time with no treatment before relapse assessment. To compare HostSim with the RMM, we develop a test we call “Virtual lung homogenate” (see Materials and Methods). All homogenate-based virtual diagnostic tests assume a 10-CFU LOD that can detect non-replicating Mtb, as true experimental LOD is challenging to estimate. For this reason, we only claim qualitative comparisons.

We find that our simulations recapitulate both RMM and NHP experimental study trends, though relapse rates are closer to rates measured from animals whose granulomas contain caseum (NHPs and C3HeB/FeJ mice) than other mice (Table 2). As discussed in “HostSim simulates Mtb-immune interactions within heterogeneous primate-like virtual hosts,” we captured NHP relapse rates after HR + SIV using Virtual PET/CT, though all virtual hosts had at least one granuloma with >10 CFU by follow-up. While HostSim recapitulates relapse rates of several experimental RMMs (treated with RMZ and HRZ), we found that HostSim predicts higher relapse rates than RMMs for three regimens (HRZE, RMZE, BPaMZ). For HRZE and RMZE, the relapse rates were higher in HostSim than in C3HeB/FeJ mice, which in turn had higher relapse rates than other RMMs. The relapse rate following BPaMZ treatment was substantially lower in RMMs compared with HostSim, likely due to the limited ability of bedaquiline and pretomanid to penetrate into caseum (18, 84), which is absent in the RMM.

**TABLE 2:** Comparison between animal relapse studies and analogous simulated relapse rates^*b*^

Replicated *in vivo* animal species studied for relapse	Treatment regimen	*In vivo* relapse %	Virtual relapse % out of analogous sub-cohort
NHP (29)	HR with comorbid SIV	67%	51% (56/109) (Virtual PET/CT)
		72%^*a*^	100% (109/109) (Virtual granuloma homogenate)
Mouse (41)	2RMZ + 2RM	42%	50% (52/104)
Mouse (33)	2HRZ + 1 HR	87%	74% (78/105)
C3HeB/FeJ mouse (34)	2HRZE + 2 HR	13-27%	53% (55/104)
Mouse (34)	2HRZE + 2 HR	0-7%	
C3HeB/FeJ mouse (34)	2RMZE + 1RM	20-60%	41% (43/104)
Mouse (34)	2RMZE + 1RM	0-20%	
Mouse (32)	1BPaMZ + 1BPaM	7%	48% (50/104)

^*a*^ This work did not measure relapse with CFU; this percentage reflects what percentage of NHPs had detectable lung CFU at time of assessment out of all NHPs whose CFU could be counted (see original study [29] Fig. 3C]).

^*b*^ The first two columns show the reference animal relapse study and treatment regimen (see Materials and Methods for duration and dosage). The third and fourth columns show the corresponding *in vivo* and simulated relapse rates, respectively.

### Translating HostSim relapse to clinical studies

We apply antibiotic treatment to HostSim to recreate human clinical relapse studies and to distinguish frequency of reservoir- and threshold-driven relapse in the human context. We show that in the clinical case, cure-screened associated relapse is likely reservoir driven.

#### HostSim captures qualitative trends of human clinical studies studying post-treatment relapse

To ensure that HostSim can bridge both experimental animal and clinical human relapse, we recreate several human clinical relapse studies. For each, we allow the full virtual cohort of *n* = 500 virtual hosts to develop a mature Mtb infection, separately administer multiple indicated antibiotic regimens, then simulate hosts post-treatment for the indicated follow-up time before testing to assess cure or relapse. To capture the effect of study inclusion criteria, we examine relapse in virtual sub-cohorts based on human-like active-TB classification for fair comparison. Virtual hosts are screened for cure at treatment completion (except where noted) and assessed for relapse at follow-up time using virtual diagnostic tests analogous to the *in vivo* study (see Materials and Methods).

Treatment of HostSim captures trends of human relapse studies (Table 3). Simulations show a decrease in relapse rates when using more potent and longer regimens, though simulated relapse rates are slightly higher than in clinical data sets. When simulating clinical studies, we find that HostSim provides an upper bound for human relapse likelihood, capturing more aggressive Mtb infection than the human data. Both real and simulated relapse rates are far higher in associated studies where cure-screening is not performed. Together, the results from this and the previous section show that a single model can mechanistically recapitulate relapse from multiple experimental species and study designs, and that those reported relapse rates likely are associated with several aspects of study design, notably including follow-up time and cure screening.

**TABLE 3:** Comparison between human clinical relapse studies and analogous simulated relapse rates^*d*^

Replicated *in vivo* diagnostic test used to study human relapse	Treatment regimen	Follow-up time post treatment-completion	*In vivo* relapse %	Virtual relapse % out of analogous sub-cohort	Notes
Sputum culture conversion (72–74)	2HRZE + 4HR	14 days^*a*^	60%	88% (55/63)	Analogous simulations do not screen for cure.
		28 days^*a*^	40%	67% (42/63)	
		60 days^*a*^	10%	22% (14/63)	
PET/CT inflammation recurrence (42)	2HRZE + 4HR	6 months	12%^*b*^	43% (22/51)	Granuloma-scale test resolution measure via PET/CT
Rapid test (85)	2HRZE + 4 HR^*c*^	2 years	2.2%	16% (60/378)	Clinical study admitted patients with any positive culture.

^*a*^ This did not measure “relapse” but rather was “still culture-positive during continuing treatment.” This was still discussed as potential for relapse in literature (12).

^*b*^ This is a simplified percentage of 12/99 hosts with recurrent disease post-treatment; actual relationship between PET/CT inflammation and Mtb sterilization is discussed in the original study (42).

^*c*^ Actual regimens unknown; we assume that these followed the standard of care.

^*d*^ The first three columns describe the reference human relapse study, treatment regimen (see Materials and Methods for duration and dosage), and follow-up assessment times used to determine relapse. In the fourth and fifth columns, we show the corresponding *in vivo* and simulated relapse rates, respectively.

#### Screening for cure at treatment-end associates with subsequent relapse in simulations

**Fig 5:**
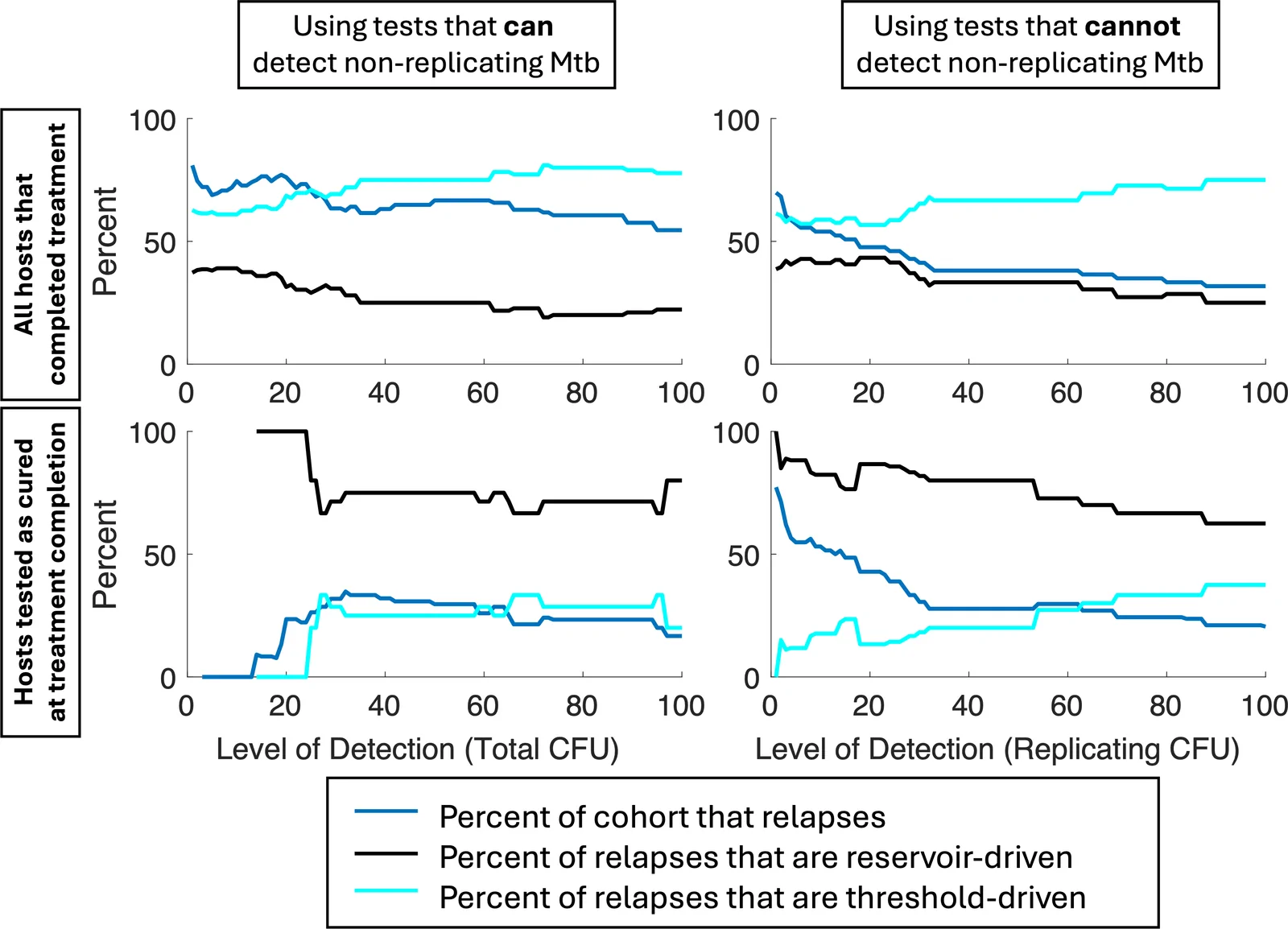
Reservoir versus threshold relapse rates by test LOD. Each of our plots show how simulated relapse rates and the relative frequency of their driving mechanisms are impacted by LOD of the diagnostic test used to assess cure and/or relapse. These analyses used the Virtual Mtb plate test to mimic sputum culture conversion tests 1 year after active TB hosts complete 2 months of virtual HRZE treatment (see Materials and Methods). We repeated each analysis assuming the virtual Mtb plate either can (left column) or cannot (right column) detect non-replicating bacteria. We also repeated each analysis varying whether relapse is defined of all hosts that complete treatment (top row) or only those that are misdiagnosed as “cured” upon treatment completion (bottom row).

In a simulated relapse study, we show that cure-screened relapse is most likely associated with reservoir-driven, regardless of diagnostic test accuracy. Specifically, we examine simulated rates of relapse one year after administering a 2-month short-course treatment of HRZE to 500 virtual hosts. We test for Mtb both at time of treatment completion and one year later by using Virtual Mtb plate (see Materials and Methods), varying the LOD of both initial and follow-up tests from 0 to 100 CFU. We also vary whether or not virtual diagnostics can detect non-replicating CFU. Extremely sensitive tests are unlikely to classify patients as cured, especially if they detect non-replicating bacteria (Fig. 5, bottom row). As tests become less sensitive, more virtual hosts diagnose as cured with higher levels of MDxC. Fig. 5 shows that in simulations, more than half of cure-screened relapses are associated with reservoir-driven relapse, regardless of LOD or test ability to detect non-replicating Mtb. All relapses are more frequently threshold-driven (>50%) if the diagnostic tests do not detect non-replicating bacteria. We observe the same outcome when simulating RMZE (File SA6), indicating that this trend maintains for regimens including drugs with more in-caseum bactericidal activity than isoniazid (here, moxifloxacin [86]).

## DISCUSSION

**Fig 6:**
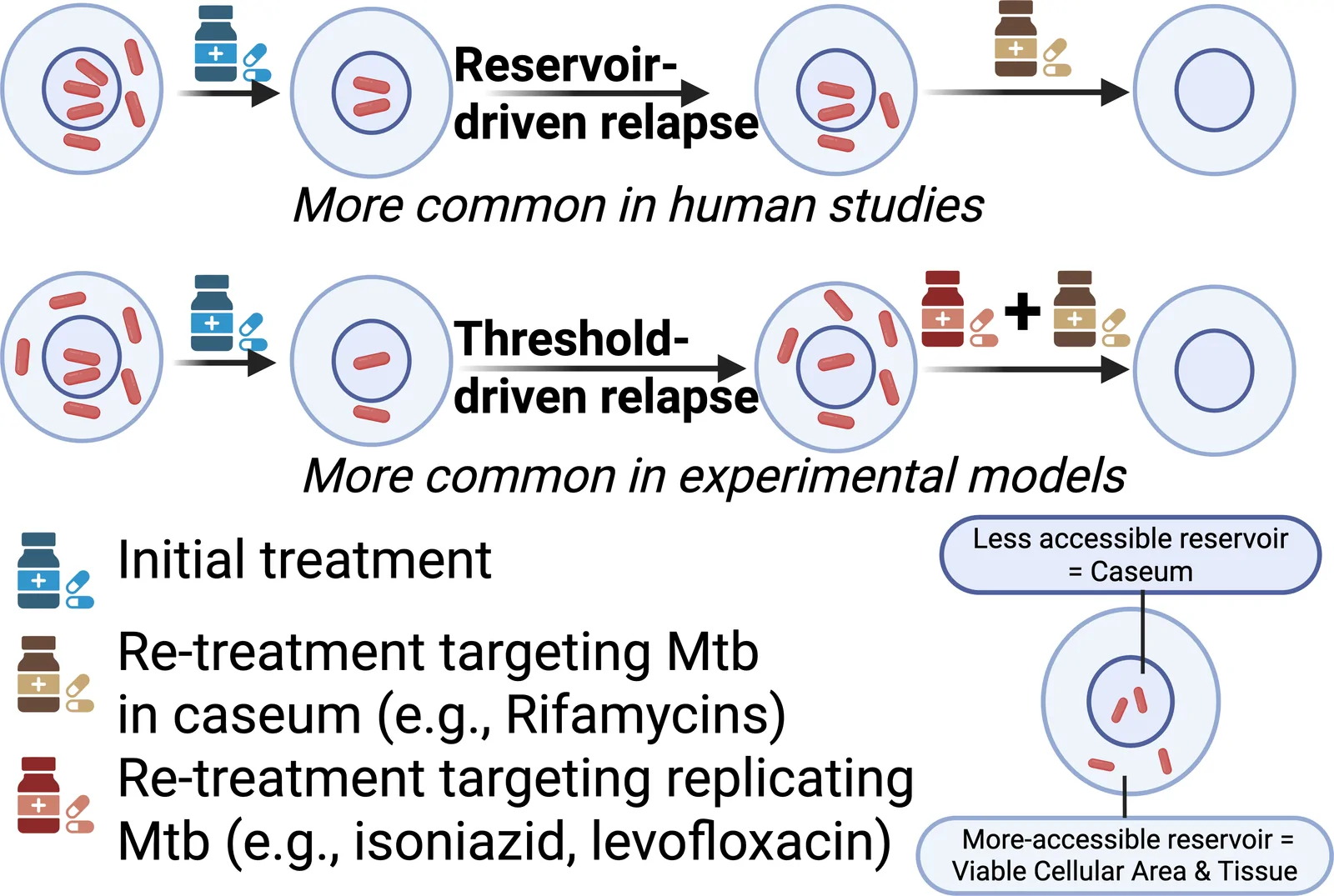
Re-treatment of multiple types of tuberculosis relapse. In simulations, we found that both reservoir-driven relapse (top row) and threshold-driven relapse (bottom row) were at play. Simulations of relapse that followed clinical study design, characterized by cure screening and longer antibiotic regimens, more often resulted in reservoir-driven. By contrast, simulations that followed animal-like simulation study design were associated with threshold-driven. These different types of relapses likely require different types of re-treatment, focusing on non-replicating mtb or complete sterilization of replicating Mtb, respectively. (Created in BioRender [C. Michael, 2026, https://BioRender.com/mjepmxz].)

Understanding how to safely shorten antibiotic treatment for TB is a critical step towards eradicating the world’s leading cause of death by single infectious agent. Aside from development of resistance, the main threat of shortening treatment regimen administration is the occurrence of relapse, where treated infections later recur. Experimental studies on relapse are often used to study TB, and results may or may not translate directly to a clinical picture. Additionally, neither experimental nor clinical relapse can conclusively determine which of two hypothesized mechanisms of relapse drive what is observed in the clinic: reservoir-driven relapse (caused by non-replicating Mtb) and/or threshold-driven relapse (caused by incomplete sterilization of intracellular-Mtb) (12). Moreover, it is not clear whether these mechanisms equally underpin clinical relapse or *in vivo* experimental models. Understanding relapse is critical as current guidelines for re-treatment of drug-susceptible TB are thin and based only on observation (87), leaving us little mechanistic understanding as we try to shorten treatment. Additionally, how experimental animal studies of relapse translate to clinical predictions needs more clarity. To address these issues regarding clinical relapse, we created a computational model that can reproduce both clinical and experimental relapse studies. Our goals were to better understand mechanisms of relapse, identify ways to better re-treat patients who undergo relapse, and provide a bridge between relapse seen in experimental animal models and the human clinical studies. Fig. 6 provides a high-level overview of our combined findings.

Understanding relapse requires separation of two often-conflated concepts: (i) the mechanisms behind the recurrence of Mtb infection and (ii) understanding that study design choices may cause some infection recurrences to be associated with relapse rather than treatment-failure. Our simulations suggest that many Mtb-infected hosts with recurrent infection would be classified as “treatment failure” rather than relapse, had cure-screening been performed upon treatment completion. Cure-screening itself does not impact whether Mtb infection will recur after treatment—surviving Mtb will replicate whether we observe them or not—but may impact how that treatment is classified. Moreover, relapses that occurred despite cure-screening were associated with reservoir-driven relapse—i.e., relapse attributed to Mtb survival within a non-replicative reservoir inaccessible to tests (Fig. 6). Reservoir-driven relapses take a longer time in simulations, as non-replicating Mtb need to be transported out of that reservoir (e.g., by neutrophils) before it can begin to replicate (Fig. 1B). By contrast, Mtb-infected hosts that were presumed cured upon treatment completion without a test and then later returned with recurrent TB (i.e., all recurrent infections) were associated with threshold-driven relapse (i.e., had low levels of Mtb surviving in a replicative reservoir). The association between cure-screening and reservoir-driven relapse was robust for all of the virtual diagnostic tests we examine, including varying whether the test could detect non-replicating CFU and a LOD varied between 1 and 100 CFU (Fig. 5). Exceptions become more likely in patients that begin treatment with higher bacterial burdens; thus, future studies may find it fruitful to report relapse events stratified by pre-treatment CFU (e.g., high burden vs low burden of disease) if possible. Finally, note that these results are not discussing the probability of relapse; indeed, predicting relapse from pre-treatment features is challenging (29). Rather, our simulated results speak to the relative likelihood of mechanisms underpinning relapse when it occurs.

Current re-treatment guidelines for drug-susceptible TB are to re-administer standard treatment (87), though our simulations suggest that how relapse was determined may inform re-treatment. In humans or animal models that form Mtb-controlling granulomas, follow-up studies may find that effective re-treatment involves using antibiotic regimens that effectively penetrate into caseum and kill Mtb trapped there in a non-replicating state—e.g., rifamycins, such as rifapentine, rifabutin, rifalazil (for pre-clinical work), and to a lesser extent moxifloxacin (86, 88, 89). If our model assumption is indeed correct that reservoir-driven relapse is characterized by a slow, continuous migration of Mtb from caseum, re-treatment regimens may be well-supplemented by antibiotics that excel at killing non-caseum-bound bacteria, e.g., bedaquiline or moxifloxacin (90, 91). Unscreened patients may benefit from re-treatment that aggressively targets replicating Mtb first, using antibiotics with high EBA scores like isoniazid, moxifloxacin, or levofloxacin (61), then re-screen and treat as in the reservoir-driven relapse case.

Our computational model bridges relapse studies in both human and animal contexts. We simulated reasonable rates of misdiagnosed cure after a variety of short-course antibiotic treatment regimens, focusing on antibiotics presently used in standard-of-care treatments such as HRZE (9, 19) (see Materials and Methods). Simulations suggest that several regimens, including HRZE and most mono-treatments, are far more likely to result in MDxC, where non-sterile infection falls below a LOD at the time of treatment completion, but is not cleared (Fig. 3). To capture mechanisms of how MDxC leads to relapse, we also incorporated a mechanism allowing non-replicating Mtb to be transported from caseum, allowing for new granuloma reseeding as we have done previously (78). With it, we were able to simulate a recent short-course treatment relapse study in NHPs (29).

Notably, we find that reported relapse rates are considerably impacted by study-specific relapse definition—specifically, whether it is based on (i) cure status upon treatment completion (not standardized between clinical and experimental studies) and (ii) whether subclinical patients are admitted to relapse studies (Tables 1 and 3). This remains true when recreating several other relapse studies in humans, mice, and NHPs. When analyzing data sets as closely as possible to their *in vivo* counterparts, we recapitulate qualitative trends of relapse (e.g., longer treatment duration leads to lower rates of relapse). However, we also find a paucity of calibration-relevant measurements in the literature, such as LOD of many diagnostic tests used. Our simulated relapse rates are higher than those reported in literature (Table 2). Higher relapse rates are reasonable since our virtual diagnostic tests are optimistically sensitive, whereas true sensitivity based on Mtb in sputum or BALF is likely lower; in the absence of literature connecting granuloma and sputum or BALF CFU, we assume one replicating CFU in the lungs corresponds to one CFU in a sputum or BAL sample. For RMMs, our relapse rates are closer to those observed in caseum-forming C3HeB/FeJ mice than non-caseum-forming BALB/c mice. The low rates of relapse among non-C3HeB/FeJ mice reinforce that without caseum, RMMs exclusively capture threshold-driven relapse. Our simulations show that threshold-driven relapse is unlikely to occur for lower-burden hosts who are treated for longer periods of time, especially in populations screened for cure.

Calibrating and validating a relapse model pose delicate problems, as calibration targets (such as reactivation rates) and validation targets (such as MDxC trends and NHP relapse rates) do not come from a single or full population of relapsing Mtb-infected individuals. Typically, to validate a computational model of, say, relapse, we would generate several virtual hosts that mechanistically recapitulate a relapse-like phenomenon. The multitude of measurements and proxies for relapse, gathered by study design, do not collectively paint a picture of a single “type of relaps"” *a priori*. Indeed, our results tell us that a host’s species, state prior to treatment, and the proxy chosen for experimental relapse may more selectively detect threshold than reservoir relapse. Likewise, whether the host was cure-screened, and the method of that screening may also inform which phenomena we associate with relapse versus treatment failure. Each of these factors must be carefully considered when translating the conclusions of any relapse model (including HostSim) back to the clinical context.

We noticed that some measures of relapse that employ extremely sensitive tests may capture perseverance of subclinical disease with Mtb levels below LOD, rather than symptomatic clinical relapse. Proxies for relapse from animal models (e.g., Mtb-positivity of tissue homogenates) may implicitly classify as relapse even if only small amounts of Mtb are present, which may more closely resemble delayed, bacteriologically detectable asymptomatic infection after a MDxC (see dark green line in Fig. 4). Still, information is valuable as recurrent subclinical disease is plausibly a silent contributor toward drug resistance with regimen-specific risks. An absence of data surrounding how diagnostic test sensitivity relates to symptoms leaves little guidance for further simulations, as simulation results depend on assumed LODs.

We have made several important simplifications to reduce model complexity. We do not model drug resistance, though drug resistance is believed to correlate with relapse rates (16). Lymph nodes are known to be important reservoirs of Mtb during TB disease and relapse, so we will combine our recent model of lymph node infection into HostSim in future work to see how this impacts our predictions (6, 21, 23, 29). Moreover, we calibrate our reservoir-like mechanism using reactivation rates subsequent to TNFα depletion (see Materials and Methods), which may ignore subtleties of TB disease during immunosuppression, evident in how TNFα-induced reactivation of TB still often test skin-test negative, and may have other qualitatively distinct immune factors (12). Another simplifying assumption in HostSim is that non-replicating Mtb all reside within caseum (Fig. 1A); in reality, some non-immune cell types like fibroblasts, type II pneumocytes, and endothelial cells can also harbor non-replicating Mtb (92); Mtb may also transition toward non-replicating metabolic states when stressed by factors like hypoxia or antibiotics (93). Our simulations do not capture lung cavitation, and the animal models we used for calibration did not exhibit significant cavitary disease. Lung cavitation is a known risk factor for relapse (94, 95) that bears distinctions from non-cavitating disease dynamics, including differences in oxygen concentration and recommended treatment, results in extremely high levels of CFU in lung tissue and sputum (94, 96, 97). This all suggests that lung cavities are more-accessible reservoirs. It may be that cavities are challenging to completely clear with antibiotics (threshold-driven relapse) or that the cavities are easily re-seeded from non-replicative reservoirs (reservoir-driven relapse). Without mechanism and data to establish this connection, our simulation cannot inform a recommended re-treatment from a principled standpoint. Finally, the potential for diagnostic tests to yield false-positive results follows from test-specific causes that are beyond the scope of this work. For example, simulating false-positive results from immune assays likely requires detailed representations of markers used in that assay, whereas predicting false-positive results from culture-based tests would depend on detailed representation of BALF sampling or laboratory contamination.

There are gaps in literature precluding any comprehensive model of relapse. Virtual diagnostics rely on a coarse-grain representation of patient states—e.g., predicting symptoms via the fold-increase of CFU over 200 days, or assuming direct proportionality between CFU in BALF, sputum, and lung tissue. Such abstractions are necessary as biological studies relating host infection state appear noisy, and it is not mechanistically clear why some hosts with active TB are culture-negative while some subclinical hosts are culture-positive (4, 98). Our simulations cannot mechanistically distinguish between LTBI and subclinical disease as we lack the mechanistic detail connecting lung state. to these clinical markers. However, HostSim (as many model frameworks) is modular, allowing us to refine components as data sets become available to form a more comprehensive symptom and diagnostic model of TB to predict relapse. One major benefit of being able to predict within-host Mtb and diagnostic-relevant inflammatory responses from patient-specific data sets is the potential to create a TB treatment digital twin, a personalized model that uses patient data to forecast specific patient outcomes during treatment. Digital twins have successfully been created for a variety of complex diseases but require a method to use limited patient-specific data sets to inform model states (99–102). Once it is possible to predict infection state from patient data sets, we would be able to create a TB relapse digital twin that could provide case-personalized decision support.

In conclusion, simulations based on a combination of human and NHP data sets can plausibly recreate observed relapse rates, but those relapse rates are a union of both reservoir- and threshold-driven relapse. In regimens that effectively penetrate caseum, simulations well-match murine relapse rates, and do not otherwise (though are closer to caseum-forming C3HeB/FeJ mice in this case). On the other hand, it is challenging to link observed human relapse without leaning on Mtb burden as a proxy until we know more about how continuously Mtb sheds from caseum into BALF, or how this relates to clinical symptoms. It appears that, at least while assuming caseum-bound Mtb are not clinically detectable, screening for cure within individual hosts as part of relapse study design essentially removes from clinical studies the very threshold-driven relapse events best modeled in mice. While both types of relapse are relevant at bedside, in clinical trials, and in experiments; separating studies of relapse risk by-mechanism may shed light on overall treatment efficacy, particularly for regimens that are caseum-limited.

## MATERIALS AND METHODS

Recently, we developed a detailed and complex *in silico* whole-host model of pulmonary Mtb infection and treatment, HostSim (62, 66, 67, 103). Model details are in “Overview of HostSim” and on the online technical description of HostSim at http://malthus.micro.med.umich.edu/lab/supplements/Host-Sim-3/. HostSim contains a whole-host PK/PD model, which we used to perform virtual pre-clinical trials to characterize bactericidal activity of various antibiotic regimens (62).

For all analyses, we generate and calibrated a set of *n* = 500 virtual hosts, each with 13 primary granulomas. Parameter values defining each host were sampled using the Laten hypercube sampling method from calibrated and validated parameter ranges, as previously described (62). Our untreated virtual cohort has 89.2% virtual hosts with LTBI, 0.8% sterilizing, and 10% virtual hosts with active TB disease at 300 days post-infection.

### Overview of HostSim

Briefly, HostSim is a multi-scale hybrid computational model of pulmonary TB, including multiple lung granuloma agents within a single virtual host lung that is coordinated with both a blood compartment and an uninfected virtual lymph node system (62, 66). As pulmonary TB is typically contained to these three compartments, we refer to this as a “whole host” for TB. Each virtual granuloma is defined by a set of ordinary differential equations that describe interactions between host immune cells (CD4+ T cells, CD8+ T cells, and macrophages) communicating, polarizing, and differentiating in response to cytokine signals (IFN-γ, TNFα, IL-4, IL-10, and IL-12). These immune cells respond to three distinct Mtb subpopulations: intracellular (within macrophages), extracellular within the granuloma, and non-replicating. We model all non-replicating Mtb as being trapped within caseum. As Mtb are killed, antigen accumulates and traffics to the lung draining lymph node system, which is represented as another set of ODEs. The lymph node ODE tracks priming of Mtb-specific CD4+ and CD8+ immune cells that migrate to the host’s blood, which is the third and final set of ODEs in HostSim. CD4 + and CD8+ Mtb-specific and nonspecific T cells may then be recruited to the virtual lung granulomas based on the granuloma state. This model also has stochastic elements such as granuloma dissemination (i.e., seeding of new granulomas) and the reservoir-driven mechanism of Mtb transport from caseum to macrophages.

Each ODE term and inter-physiological compartmental transition term describe key dynamics of pulmonary TB, including both untreated infection progression and the PK/PD governing treatment efficacy. The terms in HostSim include (i) metabolic differences between replicating Mtb (internalized by macrophages or not) and non-replicating Mtb, (ii) dynamic priming of T cells and the (de)activation of monocyte-derived macrophages, (iii) the dynamic accumulation of caseum based on macrophage necrosis, (iv) antibiotic penetration into caseum, and (v) synergistic/antagonistic drug-drug impacts on PD. Details of parameter ranges and ODEs are in File SB.

### Classifying virtual host state

To systematically examine case-specific patient outcomes with or without treatment, we need to determine how those outcomes are measured.

#### Classifying virtual host subclinical versus active TB by species

Estimating which virtual hosts suffer active disease is a nuanced challenge, as there is no currently known mechanism connecting CFU (lung or BALF) to symptoms. As an estimate, if CFU levels reach a species-specific threshold for fold increase over when we would have expected hosts with LTBI to stabilize (~200 days), then we label the hosts as having active TB. We also predict hosts with large-CFU levels (>10,000 for over 30-day timeframe) also experience active TB, as we have in previous work (62). Hosts with CFU levels below a given LOD are listed as “sterilizing” (as we assume clearance cannot be distinguished from paucibacillary infection). All other virtual hosts are considered to be subclinical or LTBI. See File SA4 for more details.

#### Virtual diagnostic tests

Clinically or experimentally, disease state is assessed using one or more diagnostics (Table SA1). To assess virtual host outcomes, we mimic those detection methods *in silico* using virtual diagnostics (Table 4). These are the building blocks of testing for more complex outcomes like relapse or reactivation. By tuning each of the virtual diagnostic tests in Table 4, we are able to mimic most of the virtual diagnostic tests used to assess host disease state *in vivo* (Table SA1).

**TABLE 4:** Virtual diagnosis overview. Each virtual diagnostic test has free parameters adjustable to mimic one or more real diagnostics

Diagnostic test name	Brief description	Free parameters in their calculation
Virtual Mtb plate	Evaluating for presence of Mtb derived from a patient sample. Patient sample can be derived from a lung homogenate culture, smear, etc. Descriptions of how we configure Virtual Mtb plate to mimic various tests are in Materials and Methods. Binary—returns 1 (not cured) or 0 (cured).	– LOD – Ratio of CFU in sample to CFU in lung (or granuloma) – Whether non-replicating Mtb may be present in virtual sample – Whether or not recently dead Mtb may be counted towards CFU levels
Virtual PET/CT	Examining numbers of metabolically active immune cells in granulomas allows for the prediction of 18F-fluorodeoxyglucose (FDG) avidity (output of PET/CT scans (104)). Sufficiently increasing FDG avidity indicates a positive diagnosis. Binary—returns 1 (positive increase) or 0 (no change).	– LOD – Fold-increase of FDG avidity required for a positive diagnosis – Predicted relative contribution of metabolically active immune cells toward FDG avidity (Materials and Methods).
Virtual clinic score	Implemented as a combination of the above diagnostics. We assume that if a virtual host’s CFU increase is uncontrolled or if a granuloma disseminates, they will have symptoms and receive a clinical score. Binary—returns 1 (either or both of the above are positive, likely symptomatic), or 0 (neither of the above are positive, likely asymptomatic).	– All of the parameters of Virtual Mtb Plate and Virtual PET/CT

A virtual diagnostic test takes a virtual host at a given time and assesses their infection state. All host-scale tests assume that pulmonary TB infection states can be precisely measured by looking at the lung state, but this may be expanded in future studies to include more detailed information regarding the role of lymph node infection (23).

##### Virtual Mtb plate

This virtual test recreates various culture, smear, or NAAT-based tests (Table 5) to assess a host as either cured or not cured at a given time. Virtual Mtb plates sum together CFU counts of relevant Mtb bacilli and compare them against a LOD. The test returns “not cured” if the sum of Mtb exceeds the LOD, and it returns “cured” otherwise. The exact definition for which Mtb is relevant is configured by checking a number of biological assumptions; in principle, we can calibrate these further once more biology is known.

**TABLE 5:** Choices and assumptions that we make to configure virtual Mtb plates into various clinical diagnostics *in silico*^*c*^

Replicated test	Q1 (Scale)	Q2 (What is the LOD?)	Q3 (Are non-replicating Mtb relevant?)	Q4 (Are recently dead Mtb relevant?)
Sputum or BAL culture	Host	50 CFU^*a*^	No^*a*^	No
Acid-fast Mtb smear	Host	5000 CFU	No	Yes, 10 days^*a*^
Granuloma tissue homogenate Mtb culture	Granuloma	5 CFU	Yes	No
NAAT-based rapid test	Host	16 CFU	No^*a*^	No^*a*,*b*^
Lung homogenate Mtb culture	Host	10 CFU	Yes	No

^*a*^ Assume direct proportionality between lung-tissue CFU counts and sputum/BAL CFU counts.

^*b*^ This assumes that the nucleic acid being tested decays rapidly.

^*c*^ The configuration-based questions are as follows. (1) Does the diagnostic look at the entire host (H) or a specific granuloma tissue (G)? (2) What is the LOD of the test? (3) Are non-replicating or caseum-bound bacteria detectable with this test? (4) Are dead Mtb able to be detected in this test? If so, how long can bacilli be detected for after they have been killed?.

##### Virtual PET/CT

Combined positron emission tomography (PET) and computed tomography (CT) is a radiographic measure of inflammation by measuring the metabolism of a radiolabeled sugar. A previous NHP study used PET/CT to detect relapse by the identification of new granulomas in NHPs (29). PET/CT scans are available for humans, though such scans are for research and are not standard clinical practice (12). Due to the similarity between human and NHP granulomas (e.g., typically controlling Mtb within discrete caseum-forming granulomas), we assume that a 20% increase of FDG avidity is indicative of sufficient bacterial regrowth that would be noticed and reported as relapse by most researchers and/or radiologists. We do not compare simulated PET/CT to mouse data, as the diffuse Mtb lesions in RMMs result in substantially different data sets than those we simulate (105).

As previously (103), we measure virtual FDG avidity as a weighted sum of metabolically active immune cells, including activated macrophages and T cells within each granuloma. In summary, FDG avidity is calculated as


$$\text{Virtual FDG avidity}=w_{1}\text{MR}+w_{2}\text{MI}+w_{3}\text{MA}+w_{4}\text{T0}+w_{5}\text{TE}+w_{6}\text{TEM}$$

where $\left\langle w_{i}\right\rangle =\left\langle 0,5,6,2,4,3\right\rangle$ are the relative weights of resting, infected, and activated macrophages; and primed, effector, and effector memory cell populations within a granuloma. Resting macrophages have a contribution of 0 as we assume they are at the same level of background activity as the uninvolved lung tissue.

The Virtual PET/CT test classifies hosts as relapsed if:

1. Any new granulomas disseminate (File SA5).
2. There is >20% increase of virtual FDG avidity between time of treatment completion and time of assessment for any granuloma within the host.

##### Virtual clinic score

Virtual clinic score is a combination of the above tests as a proxy for clinical evaluation. We assume that a host will present with symptoms if new granulomas continue to form, and that a clinician has access to a rapid test (e.g., Xpert [106]). A host is determined to be cured if none of the following return “not cured”:

1. A Virtual Mtb plate with LOD = 16 CFU (as described in Table 5), assuming that non-replicating bacteria cannot be detected via a rapid test such as Xpert (106) (see Table SA1).
2. If any new granulomas have formed within the host since the last virtual diagnostic, even below LOD—we assume that this is indicative of symptom recurrence as is assumed in (29).

### Mechanism for reservoir-driven relapse: stochastic transport of Mtb from caseum

To simulate both reservoir- and threshold-driven relapse, we include reservoir and threshold mechanisms in HostSim. Simulations allow an activated immune system (such as neutrophils) to transport Mtb from caseum, transitioning them to an intracellular state as suggested by our previous work (78). We assume that the reservoir mechanism is involved in both relapse and reactivation. Subclinical hosts may go years without reactivation—only 10% of subclinical infection hosts with no comorbidities reactivate at any point in their life (4, 107)—so we calibrate our reactivation mechanism for studying relapse model in the context of reactivation induced by TNFα depletion. Details are in Table SA2.

### Simulated antibiotic regimens

Relapse has been studied in the context of shortening standard antibiotic regimens. These typically include a combination of isoniazid (INH; H), rifampicin (RIF; R), ethambutol (ETH; E), and pyrazinamide (PZA; Z), and together are the historic standard treatment regimen HRZE (9). Second-line regimens for RIF-resistant TB include a combination of bedaquiline (BDQ; B), pretomanid (PTM; Pa), linezolid (LZD; L), and (sometimes) moxifloxacin (MXF; M), called BPaL(M) (7, 37). The standard treatment is 6–9 months of HRZE, or 2 months of HRZE and 4 months of HR (108). Several studies have tested for sputum culture conversion after only 2 months of antibiotic treatment (38).

We simulated two families of antibiotic regimens, summarized in Table 6. To determine variability in the level of non-detectable bacteria across multiple regimens, we reproduce a family of regimens including H, R, Z, E, B, Pa, L, and M tested in our previous work (62) (group i). We also simulate scenarios wherein virtual hosts are treated with multi-phase regimens (group ii), such as the WHO standard of care (Regimen 2HRZE4HR) that discontinues use of PZA and EMB after 2 months. After virtual treatments end, we continue simulations without treatment to examine relapse events, though regimens may be assessed at more than one follow-up time (Table 3).

**TABLE 6:** Regimens from previous studies recreated using HostSim*^d^*

Regimen name	INH	RIF	PZA	EMB	BDQ	PTM	LZD	MXF
Regimen set i—marmoset studies from a previous *in silico*/NHP study (109) (set reproduced from our previous HostSim study (62).								
RMZE	–	10	25	20	–	–	–	7
BPa	–	–	–	–	20	20	–	–
BPaL	–	–	–	–	20	20	90	–
BL	–	–	–	–	20	–	90	–
RM	–	10	–	–	–	–	–	7
HRZE	5	10	25	20	–	–	–	–
PaL	–	–	–	–	–	20	90	–
Bedaquiline	–	–	–	–	20	–	–	–
Pretomanid	–	–	–	–	–	20	–	–
RZ	–	10	25	–	–	–	–	–
HZ	5	–	25	–	–	–	–	–
Moxifloxacin	–	–	–	–	–	–	–	7
Pyrazinamide	–	–	25	–	–	–	–	–
Rifampicin	–	10	–	–	–	–	–	–
Isoniazid	5	–	–	–	–	–	–	–
Regimen set ii—multi-phase regimens used in standard of care and/or experimental relapse studies.								
2HR + SIV^*b*^	6	10	–	–	–	–	–	–
2HRZE4HR	6	10	25^*a*^	20^*a*^	–	–	–	–
2RMZ2RM^*c*^ (41)	–	10	25^*a*^	–	–	–	–	7
2HRZE2HR^*c*^ (34)	5	10	25^*a*^	20^*a*^	–	–	–	–
2RMZE1RM^*c*^ (34)	–	10	25^*a*^	20^*a*^	–	–	–	7
2HRZ1HR^*c*^ (33)	6	10	25^*a*^	–	–	–	–	–
1BPaMZ1BPaM^*c*^ (32)	–	–	25^*a*^	–	20	20	–	7

^*a*^ Antibiotic halted after initial phase.

^*b*^ Specific timing of virtual HRZE and virtual SIV are given in the results text, and are adapted from an NHP model (29).

^*c*^ Dose adjusted to the human equivalent standard dose.

^*d*^ This table is partly based on Table 5 from our previous work (62). Columns labeled by antibiotic name (e.g., INH) indicate the human-equivalent dosage of that antibiotic in human-equivalent mg/kg, administered daily. Values of “–” indicate that the antibiotic was not present in the regimen. Each regimen in set (i) lasts 6 months (180 days). Regimens in set (ii) are split into phases, with each phase duration and the drugs administered during that phase indicated by regimen name—e.g., 2HR1R indicates 2 months of INH + RIF followed by 1 month of RIF monotreatment.

## SUPPLEMENTAL MATERIAL

Supplemental material mbio.01977-26-s0001.pdf Files SA and SB.

ASM does not own the copyrights to Supplemental Material that may be linked to, or accessed through, an article. The authors have granted ASM a non-exclusive, world-wide license to publish the Supplemental Material files. Please contact the corresponding author directly for reuse.

## References

[1] World Health Organization. 2024. Global tuberculosis report 2024. World Health Organization, Geneva.

[2] CDC. 2025. Provisional 2025 tuberculosis data, United States. Tuberculosis data. Available from: https://www.cdc.gov/tb-data/aboutprovisionaldata/2025-provisional-data.html

[3] Cadena AM, Fortune SM, Flynn JL. 2017. Heterogeneity in tuberculosis. Nat Rev Immunol 17:691–702. doi:10.1038/nri.2017.69 PMC6247113 28736436

[4] Lin PL, Flynn JL. 2018. The end of the binary era: revisiting the spectrum of tuberculosis. J Immunol 201:2541–2548. doi:10.4049/jimmunol.1800993 PMC6217958 30348659

[5] Lin PL, Maiello P, Gideon HP, Coleman MT, Cadena AM, Rodgers MA, Gregg R, O’Malley M, Tomko J, Fillmore D, Frye LJ, Rutledge T, DiFazio RM, Janssen C, Klein E, Andersen PL, Fortune SM, Flynn JL. 2016. PET CT identifies reactivation risk in cynomolgus macaques with latent *M. tuberculosis*. PLoS Pathog 12:e1005739. doi:10.1371/journal.ppat.1005739 PMC4933353 27379816

[6] Dutta NK, Karakousis PC. 2014. Latent tuberculosis infection: myths, models, and molecular mechanisms. Microbiol Mol Biol Rev 78:343–371. doi:10.1128/MMBR.00010-14 PMC4187682 25184558

[7] Dheda K, Gumbo T, Gandhi NR, Murray M, Theron G, Udwadia Z, Migliori GB, Warren R. 2014. Global control of tuberculosis: from extensively drug-resistant to untreatable tuberculosis. Lancet Respir Med 2:321–338. doi:10.1016/S2213-2600(14)70031-1 PMC5526327 24717628

[8] Saukkonen JJ, Duarte R, Munsiff SS, Winston CA, Mammen MJ, Abubakar I, Acuña-Villaorduña C, Barry PM, Bastos ML, Carr W, et al.. 2025. Updates on the treatment of drug-susceptible and drug-resistant tuberculosis: an official ATS/CDC/ERS/IDSA clinical practice guideline. Am J Respir Crit Care Med 211:15–33. doi:10.1164/rccm.202410-2096ST PMC11755361 40693952

[9] World Health Organization. 2022. WHO consolidated guidelines on tuberculosis. Module 4: treatment - drug-resistant tuberculosis treatment, 2022 update. World Health Organization, Geneva. 36630546

[10] Global TB Programme. 2018. Latent tuberculosis infection: updated and consolidated guidelines for programmatic management. World Health Organization, Global TB Programme, Geneva, Switzerland. 30277688

[11] World Health Organization. 2022. Implementing the end TB strategy: the essentials, 2022 update

[12] Goletti D, Lindestam Arlehamn CS, Scriba TJ, Anthony R, Cirillo DM, Alonzi T, Denkinger CM, Cobelens F. 2018. Can we predict tuberculosis cure? What tools are available? Eur Respir J 52:1801089. doi:10.1183/13993003.01089-2018 30361242

[13] Frank DJ, Horne DJ, Dutta NK, Shaku MT, Madensein R, Hawn TR, Steyn AJC, Karakousis PC, Kana BD, Meintjes G, Laughon B, Tanvir Z. 2019. Remembering the host in tuberculosis drug development. J Infect Dis 219:1518–1524. doi:10.1093/infdis/jiy712 PMC6562157 30590592

[14] Danchik C, Wang S, Karakousis PC. 2021. Targeting the *Mycobacterium tuberculosis* stringent response as a strategy for shortening tuberculosis treatment. Front Microbiol 12:744167. doi:10.3389/fmicb.2021.744167 PMC8529327 34690990

[15] Karakousis PC, Mooney G. 2025. Respiratory isolation for tuberculosis: a historical perspective. J Infect Dis 231:3–9. doi:10.1093/infdis/jiae477 PMC12187122 39373220

[16] Colangeli R, Jedrey H, Kim S, Connell R, Ma S, Chippada Venkata UD, Chakravorty S, Gupta A, Sizemore EE, Diem L, Sherman DR, Okwera A, Dietze R, Boom WH, Johnson JL, Mac Kenzie WR, Alland D, DMID 01-009/Tuberculosis Trials Consortium Study 22 Teams. 2018. Bacterial factors that predict relapse after tuberculosis therapy. N Engl J Med 379:823–833. doi:10.1056/NEJMoa1715849 PMC6317071 30157391

[17] Ramakrishnan L. 2012. Revisiting the role of the granuloma in tuberculosis. Nat Rev Immunol 12:352–366. doi:10.1038/nri3211 22517424

[18] Sarathy JP, Liang H-PH, Weiner D, Gonzales J, Via LE, Dartois V. 2017. An *in vitro* caseum binding assay that predicts drug penetration in tuberculosis lesions. J Vis Exp e55559:55559. doi:10.3791/55559 PMC5607931 28518128

[19] Budak M, Cicchese JM, Maiello P, Borish HJ, White AG, Chishti HB, Tomko J, Frye LJ, Fillmore D, Kracinovsky K, Sakal J, Scanga CA, Lin PL, Dartois V, Linderman JJ, Flynn JL, Kirschner DE. 2023. Optimizing tuberculosis treatment efficacy: comparing the standard regimen with moxifloxacin-containing regimens. PLoS Comput Biol 19:e1010823. doi:10.1371/journal.pcbi.1010823 PMC10306236 37319311

[20] Zhan L, Tang J, Sun M, Qin C. 2017. Animal models for tuberculosis in translational and precision medicine. Front Microbiol 8:717. doi:10.3389/fmicb.2017.00717 PMC5415616 28522990

[21] Ganchua SKC, White AG, Klein EC, Flynn JL. 2020. Lymph nodes-the neglected battlefield in tuberculosis. PLoS Pathog 16:e1008632. doi:10.1371/journal.ppat.1008632 PMC7425845 32790739

[22] Lin PL, Ford CB, Coleman MT, Myers AJ, Gawande R, Ioerger T, Sacchettini J, Fortune SM, Flynn JL. 2014. Sterilization of granulomas is common in active and latent tuberculosis despite within-host variability in bacterial killing. Nat Med 20:75–79. doi:10.1038/nm.3412 PMC3947310 24336248

[23] Krupinsky KC, Michael CT, Nanda P, Mattila JT, Kirschner D. 2025. Distinguishing multiple roles of T cell and macrophage involvement in determining lymph node fates during *Mycobacterium tuberculosis* infection. PLoS Comput Biol 21:e1013033. doi:10.1371/journal.pcbi.1013033 PMC12084042 40334195

[24] Choudhury A, Shukla J, Mahajan G, Jha DK, Gupta P, Sharma V. 2021. Hepatic tuberculosis: myriad of hues. GERMS 11:310–313. doi:10.18683/germs.2021.1266 PMC8373414 34422701

[25] Dutta NK, Klinkenberg LG, Vazquez M-J, Segura-Carro D, Colmenarejo G, Ramon F, Rodriguez-Miquel B, Mata-Cantero L, Porras-De Francisco E, Chuang Y-M, Rubin H, Lee JJ, Eoh H, Bader JS, Perez-Herran E, Mendoza-Losana A, Karakousis PC. 2019. Inhibiting the stringent response blocks *Mycobacterium tuberculosis* entry into quiescence and reduces persistence. Sci Adv 5:eaav2104. doi:10.1126/sciadv.aav2104 PMC6426458 30906866

[26] Balaban NQ, Helaine S, Lewis K, Ackermann M, Aldridge B, Andersson DI, Brynildsen MP, Bumann D, Camilli A, Collins JJ, Dehio C, Fortune S, Ghigo J-M, Hardt W-D, Harms A, Heinemann M, Hung DT, Jenal U, Levin BR, Michiels J, Storz G, Tan M-W, Tenson T, Van Melderen L, Zinkernagel A. 2019. Definitions and guidelines for research on antibiotic persistence. Nat Rev Microbiol 17:441–448. doi:10.1038/s41579-019-0196-3 PMC7136161 30980069

[27] ASPE. 2014. Examination of clinical trial costs and barriers for drug development. ASPE. Available from: http://aspe.hhs.gov/reports/examination-clinical-trial-costs-barriers-drug-development-0

[28] Flynn JL, Capuano SV, Croix D, Pawar S, Myers A, Zinovik A, Klein E. 2003. Non-human primates: a model for tuberculosis research. Tuberculosis (Edinburgh) 83:116–118. doi:10.1016/s1472-9792(02)00059-8 12758200

[29] Maiello P, Diedrich C, Rutledge T, Rodgers M, Kracinovsky K, Borish HJ, White A, Hopkins F, Chao MC, Klein E, Fortune S, Flynn JL, Lin PL. 2025. Characterizing PET CT patterns and bacterial dissemination features of tuberculosis relapse in the macaque model. Infect Immun 93:e00177-25. doi:10.1128/iai.00177-25 PMC12341373 40548727

[30] Capuano SV 3rd, Croix DA, Pawar S, Zinovik A, Myers A, Lin PL, Bissel S, Fuhrman C, Klein E, Flynn JL. 2003. Experimental *Mycobacterium tuberculosis* infection of cynomolgus macaques closely resembles the various manifestations of human *M. tuberculosis* infection. Infect Immun 71:5831–5844. doi:10.1128/IAI.71.10.5831-5844.2003 PMC201048 14500505

[31] Xu J, Li S-Y, Almeida DV, Tasneen R, Barnes-Boyle K, Converse PJ, Upton AM, Mdluli K, Fotouhi N, Nuermberger EL. 2019. Contribution of pretomanid to novel regimens containing bedaquiline with either linezolid or moxifloxacin and pyrazinamide in murine models of tuberculosis. Antimicrob Agents Chemother 63:e00021-19. doi:10.1128/AAC.00021-19 PMC6496099 30833432

[32] Li S-Y, Tasneen R, Tyagi S, Soni H, Converse PJ, Mdluli K, Nuermberger EL. 2017. bactericidal and sterilizing activity of a novel regimen with bedaquiline, pretomanid, moxifloxacin, and pyrazinamide in a murine model of tuberculosis. Antimicrob Agents Chemother 61:e00913-17. doi:10.1128/AAC.00913-17 PMC5571308 28630203

[33] Tasneen R, Betoudji F, Tyagi S, Li S-Y, Williams K, Converse PJ, Dartois V, Yang T, Mendel CM, Mdluli KE, Nuermberger EL. 2016. Contribution of oxazolidinones to the efficacy of novel regimens containing bedaquiline and pretomanid in a mouse model of tuberculosis. Antimicrob Agents Chemother 60:270–277. doi:10.1128/AAC.01691-15 PMC4704221 26503656

[34] Li S-Y, Irwin SM, Converse PJ, Mdluli KE, Lenaerts AJ, Nuermberger EL. 2015. Evaluation of moxifloxacin-containing regimens in pathologically distinct murine tuberculosis models. Antimicrob Agents Chemother 59:4026–4030. doi:10.1128/AAC.00105-15 PMC4468727 25918146

[35] Lambert M-L, Hasker E, Van Deun A, Roberfroid D, Boelaert M, Van der Stuyft P. 2003. Recurrence in tuberculosis: relapse or reinfection? Lancet Infect Dis 3:282–287. doi:10.1016/s1473-3099(03)00607-8 12726976

[36] Conradie F, Bagdasaryan TR, Borisov S, Howell P, Mikiashvili L, Ngubane N, Samoilova A, Skornykova S, Tudor E, Variava E, et al.. 2022. Bedaquiline-pretomanid-linezolid regimens for drug-resistant tuberculosis. N Engl J Med 387:810–823. doi:10.1056/NEJMoa2119430 PMC9490302 36053506

[37] Nyang’wa B-T, Berry C, Kazounis E, Motta I, Parpieva N, Tigay Z, Solodovnikova V, Liverko I, Moodliar R, Dodd M, Ngubane N, Rassool M, McHugh TD, Spigelman M, Moore DAJ, Ritmeijer K, du Cros P, Fielding K, TB-PRACTECAL Study Collaborators. 2022. A 24-week, all-oral regimen for rifampin-resistant tuberculosis. N Engl J Med 387:2331–2343. doi:10.1056/NEJMoa2117166

[38] Lanoix J-P, Chaisson RE, Nuermberger EL. 2016. Shortening tuberculosis treatment with fluoroquinolones: lost in translation? Clin Infect Dis 62:484–490. doi:10.1093/cid/civ911 PMC5006403 26527614

[39] Benator D, Bhattacharya M, Bozeman L, Burman W, Cantazaro A, Chaisson R, Gordin F, Horsburgh CR, Horton J, Khan A, Lahart C, Metchock B, Pachucki C, Stanton L, Vernon A, Villarino ME, Wang YC, Weiner M, Weis S, Tuberculosis Trials Consortium. 2002. Rifapentine and isoniazid once a week versus rifampicin and isoniazid twice a week for treatment of drug-susceptible pulmonary tuberculosis in HIV-negative patients: a randomised clinical trial. Lancet 360:528–534. doi:10.1016/s0140-6736(02)09742-8 12241657

[40] Zeruma DD, Kote M, Zerihun E, Gedabo G, Wogayehu B. 2025. Time to a tuberculosis treatment cure and its predictors among tuberculosis patients at public health facilities in Arba minch town, South Ethiopia: A retrospective cohort study. BMC Infect Dis 25:856. doi:10.1186/s12879-025-11224-7 PMC12220455 40597695

[41] Ibrahim M, Truffot-Pernot C, Andries K, Jarlier V, Veziris N. 2009. Sterilizing activity of R207910 (TMC207)-containing regimens in the murine model of tuberculosis. Am J Respir Crit Care Med 180:553–557. doi:10.1164/rccm.200807-1152OC 19590024

[42] Malherbe ST, Shenai S, Ronacher K, Loxton AG, Dolganov G, Kriel M, Van T, Chen RY, Warwick J, Via LE, et al.. 2016. Persisting positron emission tomography lesion activity and *Mycobacterium tuberculosis* mRNA after tuberculosis cure. Nat Med 22:1094–1100. doi:10.1038/nm.4177 PMC5053881 27595324

[43] Narayanan PR. 2002. Shortening short course chemotherapy: a randomised clinical trial for treatment of smear positive pulmonary tuberculosis with regimens using ofloxacin in the intensive phase. Ind J Tub 49:27–38.

[44] Carvalho ACC, Amorim G, Melo MGM, Silveira AKA, Vargas PHL, Moreira ASR, Rocha MS, Souza AB, Arriaga MB, Araújo-Pereira M, Figueiredo MC, Durovni B, Lapa-E-Silva JR, Cavalcante S, Rolla VC, Sterling TR, Cordeiro-Santos M, Andrade BB, Silva EC, Kritski AL, RePORT Brazil consortium. 2021. Pre-treatment neutrophil count as a predictor of antituberculosis therapy outcomes: a multicenter prospective cohort study. Front Immunol 12:661934. doi:10.3389/fimmu.2021.661934 PMC8284392 34276654

[45] Kendall EA, Shrestha S, Dowdy DW. 2021. The epidemiological importance of subclinical tuberculosis. a critical reappraisal. Am J Respir Crit Care Med 203:168–174. doi:10.1164/rccm.202006-2394PP PMC7874405 33197210

[46] Lin Philana Ling, Rodgers M, Smith L, Bigbee M, Myers A, Bigbee C, Chiosea I, Capuano SV, Fuhrman C, Klein E, Flynn JL. 2009. Quantitative comparison of active and latent tuberculosis in the cynomolgus macaque model. Infect Immun 77:4631–4642. doi:10.1128/IAI.00592-09 PMC2747916 19620341

[47] Coleman MT, Maiello P, Tomko J, Frye LJ, Fillmore D, Janssen C, Klein E, Lin PL. 2014. Early Changes by (18)fluorodeoxyglucose positron emission tomography coregistered with computed tomography predict outcome after *Mycobacterium tuberculosis* infection in cynomolgus macaques. Infect Immun 82:2400–2404. doi:10.1128/IAI.01599-13 PMC4019174 24664509

[48] Lin P. L., Flynn JL. 2010. Understanding latent tuberculosis: a moving target. J Immunol 185:15–22. doi:10.4049/jimmunol.0903856 PMC3311959 20562268

[49] Berry MPR, Graham CM, McNab FW, Xu Z, Bloch SAA, Oni T, Wilkinson KA, Banchereau R, Skinner J, Wilkinson RJ, Quinn C, Blankenship D, Dhawan R, Cush JJ, Mejias A, Ramilo O, Kon OM, Pascual V, Banchereau J, Chaussabel D, O’Garra A. 2010. An interferon-inducible neutrophil-driven blood transcriptional signature in human tuberculosis. Nature 466:973–977. doi:10.1038/nature09247 PMC3492754 20725040

[50] Wigginton JE, Kirschner D. 2001. A model to predict cell-mediated immune regulatory mechanisms during human infection with *Mycobacterium tuberculosis*. J Immunol 166:1951–1967. doi:10.4049/jimmunol.166.3.1951 11160244

[51] Selvavinayagam ST, Anusree A, Yong YK, Sankar S, Frederick A, Rajeshkumar M, Kumar MS, Sampath P, Sankar G, Roy CL, Karishma SJ, Murugesan A, Balakrishnan P, Govindaraj S, Byrareddy SN, Velu V, Shankar EM, Larsson M, Kannan M, Raju S. 2025. Clinical laboratory analytes and platelet-associated parameters as surrogate markers of subclinical inflammation in latent tuberculosis infection. Front Immunol 16:1662454. doi:10.3389/fimmu.2025.1662454 PMC12484228 41041335

[52] U.S. Department of Health and Human Services, Food and Drug Administration, Center for Drug Evaluation and Research. 2005. Guidance for industry: estimating the maximum safe starting dose in initial clinical trials for therapeutics in adult healthy volunteers

[53] Mordenti J. 1986. Man versus beast: pharmacokinetic scaling in mammals. J Pharm Sci 75:1028–1040. doi:10.1002/jps.2600751104 3820096

[54] Kramnik I, Beamer G. 2016. Mouse models of human TB pathology: roles in the analysis of necrosis and the development of host-directed therapies. Semin Immunopathol 38:221–237. doi:10.1007/s00281-015-0538-9 PMC4779126 26542392

[55] Lanoix J-P, Lenaerts AJ, Nuermberger EL. 2015. Heterogeneous disease progression and treatment response in a C3HeB/FeJ mouse model of tuberculosis. Dis Model Mech 8:603–610. doi:10.1242/dmm.019513 PMC4457036 26035868

[56] Plumlee CR, Duffy FJ, Gern BH, Delahaye JL, Cohen SB, Stoltzfus CR, Rustad TR, Hansen SG, Axthelm MK, Picker LJ, Aitchison JD, Sherman DR, Ganusov VV, Gerner MY, Zak DE, Urdahl KB. 2021. Ultra-low dose aerosol infection of mice with *Mycobacterium tuberculosis* more closely models human tuberculosis. Cell Host Microbe 29:68–82. doi:10.1016/j.chom.2020.10.003 PMC7854984 33142108

[57] Scanga CA, Flynn JL. 2014. Modeling tuberculosis in nonhuman primates. Cold Spring Harb Perspect Med 4:a018564. doi:10.1101/cshperspect.a018564 PMC4292094 25213189

[58] Berg A, Clary J, Hanna D, Nuermberger E, Lenaerts A, Ammerman N, Ramey M, Hartley D, Hermann D. 2022. Model-based meta-analysis of relapsing mouse model studies from the critical path to tuberculosis drug regimens initiative database. Antimicrob Agents Chemother 66:e01793-21. doi:10.1128/AAC.01793-21 PMC8923195 35099274

[59] Via LE, Schimel D, Weiner DM, Dartois V, Dayao E, Cai Y, Yoon Y-S, Dreher MR, Kastenmayer RJ, Laymon CM, Carny JE, Flynn JL, Herscovitch P, Barry CE 3rd. 2012. F]2-fluoro-deoxy-D-glucose positron emission tomography and computed tomography. Antimicrob Agents Chemother 56:4391–4402. doi:10.1128/AAC.00531-12 PMC3421588 22687508

[60] Lin PL, Dartois V, Johnston PJ, Janssen C, Via L, Goodwin MB, Klein E, Barry CE III, Flynn JL. 2012. Metronidazole prevents reactivation of latent Mycobacterium tuberculosis infection in macaques. Proc Natl Acad Sci USA 109:14188–14193. doi:10.1073/pnas.1121497109 PMC3435210 22826237

[61] Bonnett LJ, Ken-Dror G, Koh GCKW, Davies GR. 2017. Comparing the efficacy of drug regimens for pulmonary tuberculosis: meta-analysis of endpoints in early-phase clinical trials. Clin Infect Dis 65:46–54. doi:10.1093/cid/cix247 PMC5850317 28402396

[62] Michael CT, Budak M, Maiello P, Kracinovsky K, Rodgers M, Tomko J, Lin PL, Flynn J, Linderman JJ, Kirschner D. 2025. Rankings of tuberculosis antibiotic treatment regimens are sensitive to spatial scale, detection limit, and initial host bacterial burden. J Theor Biol 611:112176. doi:10.1016/j.jtbi.2025.112176 PMC12364387 40460906

[63] Cicchese JM, Sambarey A, Kirschner D, Linderman JJ, Chandrasekaran S. 2021. A multi-scale pipeline linking drug transcriptomics with pharmacokinetics predicts *in vivo* interactions of tuberculosis drugs. Sci Rep 11:5643. doi:10.1038/s41598-021-84827-0 PMC7971003 33707554

[64] Marx FM, Dunbar R, Enarson DA, Williams BG, Warren RM, van der Spuy GD, van Helden PD, Beyers N. 2014. The temporal dynamics of relapse and reinfection tuberculosis after successful treatment: a retrospective cohort study. Clin Infect Dis 58:1676–1683. doi:10.1093/cid/ciu186 24647020

[65] Shen X, Yang C, Wu J, Lin S, Gao X, Wu Z, Tian J, Gan M, Luo T, Wang L, Yu C, Mei J, Pan Q, DeRiemer K, Yuan Z, Gao Q. 2017. Recurrent tuberculosis in an urban area in China: relapse or exogenous reinfection? Tuberculosis (Edinburgh) 103:97–104. doi:10.1016/j.tube.2017.01.007 PMC5638046 28237039

[66] Joslyn LR, Linderman JJ, Kirschner DE. 2022. A virtual host model of Mycobacterium tuberculosis infection identifies early immune events as predictive of infection outcomes. J Theor Biol 539:111042. doi:10.1016/j.jtbi.2022.111042 PMC9169921 35114195

[67] Joslyn L, Linderman JJ, Kirschner D. 2020. Concomitant immunity is mediated by effector memory T cells in tuberculosis. Sci Rep. doi:10.1038/s41598-022-24516-8

[68] Gideon HP, Phuah J, Myers AJ, Bryson BD, Rodgers MA, Coleman MT, Maiello P, Rutledge T, Marino S, Fortune SM, Kirschner DE, Lin PL, Flynn JL. 2015. Variability in tuberculosis granuloma T cell responses exists, but a balance of pro- and anti-inflammatory cytokines is associated with sterilization. PLoS Pathog 11:e1004603. doi:10.1371/journal.ppat.1004603 PMC4303275 25611466

[69] Marino S, Gideon HP, Gong C, Mankad S, McCrone JT, Lin PL, Linderman JJ, Flynn JL, Kirschner DE. 2016. Computational and empirical studies predict *Mycobacterium tuberculosis*-specific T cells as a biomarker for infection outcome. PLoS Comput Biol 12:e1004804. doi:10.1371/journal.pcbi.1004804 PMC4827839 27065304

[70] Cadena AM, Hopkins FF, Maiello P, Carey AF, Wong EA, Martin CJ, Gideon HP, DiFazio RM, Andersen P, Lin PL, Fortune SM, Flynn JL. 2018. Concurrent infection with Mycobacterium tuberculosis confers robust protection against secondary infection in macaques. PLoS Pathog 14:e1007305. doi:10.1371/journal.ppat.1007305 PMC6200282 30312351

[71] Darrah PA, DiFazio RM, Maiello P, Gideon HP, Myers AJ, Rodgers MA, Hackney JA, Lindenstrom T, Evans T, Scanga CA, Prikhodko V, Andersen P, Lin PL, Laddy D, Roederer M, Seder RA, Flynn JL. 2019. Boosting BCG with proteins or rAd5 does not enhance protection against tuberculosis in rhesus macaques. NPJ Vaccines 4:21. doi:10.1038/s41541-019-0113-9 PMC6538611 31149352

[72] Fox KA, Kirwan DE, Whittington AM, Krishnan N, Robertson BD, Gilman RH, López JW, Singh S, Porter JC, Friedland JS. 2018. Platelets regulate pulmonary inflammation and tissue destruction in tuberculosis. Am J Respir Crit Care Med 198:245–255. doi:10.1164/rccm.201710-2102OC PMC6058979 29420060

[73] Ministerio de Salud. 2013. Resolución ministerial N.° 715-2013-MINSA. Available from: https://www.gob.pe/institucion/minsa/normas-legales/198713-715-2013-minsa

[74] Proaño A, Bravard MA, López JW, Lee GO, Bui D, Datta S, Comina G, Zimic M, Coronel J, Caviedes L, Cabrera JL, Salas A, Ticona E, Vu NM, Kirwan DE, Loader M-CI, Friedland JS, Moore DAJ, Evans CA, Tracey BH, Gilman RH, Tuberculosis Working Group in Peru. 2017. Dynamics of cough frequency in adults undergoing treatment for pulmonary tuberculosis. Clin Infect Dis 64:1174–1181. doi:10.1093/cid/cix039 PMC5399950 28329268

[75] Budak M, Moraes MP, Greenstein T, Maiello P, Borish HJ, Chishti HB, Kracinovsky K, Rodgers M, Tomko J, Lin PL, Flynn JL, Aldridge BB, Kirschner D. 2025. GEODE: an *in silico* tool that translates *in vitro* to *in vivo* predictions of tuberculosis antibiotic combination efficacy. Front Pharmacol 16. doi:10.3389/fphar.2025.1639673 PMC12575316 41181601

[76] Pienaar E, Dartois V, Linderman JJ, Kirschner DE. 2015. In silico evaluation and exploration of antibiotic tuberculosis treatment regimens. BMC Syst Biol 9:79. doi:10.1186/s12918-015-0221-8 PMC4650854 26578235

[77] Conradie F, Diacon AH, Ngubane N, Howell P, Everitt D, Crook AM, Mendel CM, Egizi E, Moreira J, Timm J, McHugh TD, Wills GH, Bateson A, Hunt R, Van Niekerk C, Li M, Olugbosi M, Spigelman M, Nix-TB Trial Team. 2020. Treatment of highly drug-resistant pulmonary tuberculosis. N Engl J Med 382:893–902. doi:10.1056/NEJMoa1901814 PMC6955640 32130813

[78] Hult C, Mattila JT, Gideon HP, Linderman JJ, Kirschner DE. 2021. Neutrophil dynamics affect *Mycobacterium tuberculosis* granuloma outcomes and dissemination. Front Immunol 12:712457. doi:10.3389/fimmu.2021.712457 PMC8525425 34675916

[79] Fallahi-Sichani M, Schaller MA, Kirschner DE, Kunkel SL, Linderman JJ. 2010. Identification of key processes that control tumor necrosis factor availability in a tuberculosis granuloma. PLoS Comput Biol 6:e1000778. doi:10.1371/journal.pcbi.1000778 PMC2865521 20463877

[80] Fallahi-Sichani M, Flynn JL, Linderman JJ, Kirschner DE. 2012. Differential risk of tuberculosis reactivation among anti-TNF therapies is due to drug binding kinetics and permeability. J Immunol 188:3169–3178. doi:10.4049/jimmunol.1103298 PMC3311778 22379032

[81] Pennisi E, Cohen J. 1996. Eradicating HIV from a patient: not just a dream? Science 272:1884–1884. doi:10.1126/science.272.5270.1884 8658157

[82] Kirschner DE, Webb GF. 1998. Immunotherapy of HIV-1 infection. J Biol Syst 06:71–83. doi:10.1142/S0218339098000091

[83] Sud D, Bigbee C, Flynn JL, Kirschner DE. 2006. Contribution of CD8+ T cells to control of *Mycobacterium tuberculosis* infection. J Immunol 176:4296–4314. doi:10.4049/jimmunol.176.7.4296 16547267

[84] Sarathy JP, Zuccotto F, Hsinpin H, Sandberg L, Via LE, Marriner GA, Masquelin T, Wyatt P, Ray P, Dartois V. 2016. Prediction of drug penetration in tuberculosis lesions. ACS Infect Dis 2:552–563. doi:10.1021/acsinfecdis.6b00051 PMC5028112 27626295

[85] Jafari C, Olaru ID, Daduna F, Lange C, Kalsdorf B. 2022. Rapid diagnosis of recurrent paucibacillary tuberculosis. Pathog Immun 7:189–202. doi:10.20411/pai.v7i2.565 PMC10189871 37207169

[86] Sarathy JP, Via LE, Weiner D, Blanc L, Boshoff H, Eugenin EA, Barry CE 3rd, Dartois VA. 2018. Extreme drug tolerance of mycobacterium tuberculosis in caseum. Antimicrob Agents Chemother 62:e02266-17. doi:10.1128/AAC.02266-17 PMC5786764 29203492

[87] WHO. 2017. Guidelines for treatment of drug-susceptible tuberculosis and patient care, 2017 update. Available from: https://www.who.int/publications/i/item/9789241550000

[88] Prideaux B, Via LE, Zimmerman MD, Eum S, Sarathy J, O’Brien P, Chen C, Kaya F, Weiner DM, Chen P-Y, Song T, Lee M, Shim TS, Cho JS, Kim W, Cho SN, Olivier KN, Barry CE 3rd, Dartois V. 2015. The association between sterilizing activity and drug distribution into tuberculosis lesions. Nat Med 21:1223–1227. doi:10.1038/nm.3937 PMC4598290 26343800

[89] Zhang Y, Wu R, Sun M, Li X, Fang R, Xing J, Li Z, Wen Y, Song N. 2025. Progress of anti-tuberculosis drug targets and novel therapeutic strategies. Front Microbiol 16. doi:10.3389/fmicb.2025.1637254 PMC12446298 40980328

[90] Dhillon J, Andries K, Phillips PPJ, Mitchison DA. 2010. Bactericidal activity of the diarylquinoline TMC207 against *Mycobacterium tuberculosis* outside and within cells. Tuberculosis (Edinburgh) 90:301–305. doi:10.1016/j.tube.2010.07.004 20732832

[91] Shandil RK, Jayaram R, Kaur P, Gaonkar S, Suresh BL, Mahesh BN, Jayashree R, Nandi V, Bharath S, Balasubramanian V. 2007. Moxifloxacin, ofloxacin, sparfloxacin, and ciprofloxacin against *Mycobacterium tuberculosis*: evaluation of *in vitro* and pharmacodynamic indices that best predict *in vivo* efficacy. Antimicrob Agents Chemother 51:576–582. doi:10.1128/AAC.00414-06 PMC1797767 17145798

[92] Agarwal P, Pandey P, Sarkar J, Krishnan MY. 2016. *Mycobacterium tuberculosis* can gain access to adipose depots of mice infected via the intra-nasal route and to lungs of mice with an infected subcutaneous fat implant. Microb Pathog 93:32–37. doi:10.1016/j.micpath.2016.01.004 26792675

[93] Ehrt S, Schnappinger D, Rhee KY. 2018. Metabolic principles of persistence and pathogenicity in *Mycobacterium tuberculosis*. Nat Rev Microbiol 16:496–507. doi:10.1038/s41579-018-0013-4 PMC6045436 29691481

[94] Urbanowski ME, Ordonez AA, Ruiz-Bedoya CA, Jain SK, Bishai WR. 2020. Cavitary tuberculosis: the gateway of disease transmission. Lancet Infect Dis 20:e117–e128. doi:10.1016/S1473-3099(20)30148-1 PMC7357333 32482293

[95] Guerra-Assunção JA, Houben RMGJ, Crampin AC, Mzembe T, Mallard K, Coll F, Khan P, Banda L, Chiwaya A, Pereira RPA, McNerney R, Harris D, Parkhill J, Clark TG, Glynn JR. 2015. Recurrence due to relapse or reinfection with *Mycobacterium tuberculosis*: a whole-genome sequencing approach in a large, population-based cohort with a high HIV infection prevalence and active follow-up. J Infect Dis 211:1154–1163. doi:10.1093/infdis/jiu574 PMC4354982 25336729

[96] Saeed W. 2012. Cavitating pulmonary tuberculosis: a global challenge. Clin Med 12:40–41. doi:10.7861/clinmedicine.12-1-40 PMC4953415 22372219

[97] Palaci M, Dietze R, Hadad DJ, Ribeiro FKC, Peres RL, Vinhas SA, Maciel ELN, do Valle Dettoni V, Horter L, Boom WH, Johnson JL, Eisenach KD. 2007. Cavitary disease and quantitative sputum bacillary load in cases of pulmonary tuberculosis. J Clin Microbiol 45:4064–4066. doi:10.1128/JCM.01780-07 PMC2168542 17928422

[98] Blankley S, Graham CM, Turner J, Berry MPR, Bloom CI, Xu Z, Pascual V, Banchereau J, Chaussabel D, Breen R, Santis G, Blankenship DM, Lipman M, O’Garra A. 2016. The transcriptional signature of active tuberculosis reflects symptom status in extra-pulmonary and pulmonary tuberculosis. PLoS One 11:e0162220. doi:10.1371/journal.pone.0162220 PMC5051928 27706152

[99] Fonseca LL, Böttcher L, Mehrad B, Laubenbacher RC. 2025. Optimal control of agent-based models via surrogate modeling. PLoS Comput Biol 21:e1012138. doi:10.1371/journal.pcbi.1012138 PMC11790234 39808665

[100] Knapp AC, Cruz DA, Mehrad B, Laubenbacher RC. 2024. Personalizing computational models to construct medical digital twins. bioRxiv. doi:10.1101/2024.05.31.596692 PMC12212996 40592464

[101] An G, Cockrell C. 2022. Drug development digital twins for drug discovery, testing and repurposing: a schema for requirements and development. Front Syst Biol 2:928387. doi:10.3389/fsysb.2022.928387 PMC9351294 35935475

[102] Nanda P, Budak M, Michael CT, Krupinsky K, Kirschner DE. 2023. Development and analysis of multiscale models for tuberculosis: from molecules to populations. bioRxiv. doi:10.1101/2023.11.13.566861

[103] Michael CT, Almohri SA, Linderman JJ, Kirschner DE. 2024. A framework for multi-scale intervention modeling: virtual cohorts, virtual clinical trials, and model-to-model comparisons. Front Syst Biol 3. doi:10.3389/fsysb.2023.1283341 PMC11415237 39310676

[104] Lin PL, Coleman T, Carney JPJ, Lopresti BJ, Tomko J, Fillmore D, Dartois V, Scanga C, Frye LJ, Janssen C, Klein E, Barry CE III, Flynn JL. 2013. Radiologic responses in cynomolgus macaques for assessing tuberculosis chemotherapy regimens. Antimicrob Agents Chemother 57:4237–4244. doi:10.1128/AAC.00277-13 PMC3754323 23796926

[105] Foss CA, Ordonez AA, Naik R, Das D, Hall A, Wu Y, Dannals RF, Jain SK, Pomper MG, Horti AG. 2022. PET/CT imaging of CSF1R in a mouse model of tuberculosis. Eur J Nucl Med Mol Imaging 49:4088–4096. doi:10.1007/s00259-022-05862-1 PMC9922090 35713665

[106] Steingart KR, Schiller I, Horne DJ, Pai M, Boehme CC, Dendukuri N. 2014. Xpert MTB/RIF assay for pulmonary tuberculosis and rifampicin resistance in adults. Cochrane Database System Rev 2014:CD009593. doi:10.1002/14651858.CD009593.pub3 PMC4470349 24448973

[107] Flynn JL, Chan J. 2001. Tuberculosis: latency and reactivation. Infect Immun 69:4195–4201. doi:10.1128/IAI.69.7.4195-4201.2001 PMC98451 11401954

[108] Nahid P, Dorman SE, Alipanah N, Barry PM, Brozek JL, Cattamanchi A, Chaisson LH, Chaisson RE, Daley CL, Grzemska M, et al.. 2016. Executive Summary: Official American Thoracic Society/Centers for Disease Control and Prevention/Infectious Diseases Society of America clinical practice guidelines: treatment of drug-susceptible tuberculosis. Clin Infect Dis 63:853–867. doi:10.1093/cid/ciw566 PMC6366011 27621353

[109] Budak M, Via LE, Weiner DM, Barry CE III, Nanda P, Michael G, Mdluli K, Kirschner D. 2024. A systematic efficacy analysis of tuberculosis treatment with BPaL ‐containing regimens using a multiscale modeling approach. CPT Pharmacom Syst Pharma 13:673–685. doi:10.1002/psp4.13117 PMC11015080 38404200

