# Supplementary material for: Experimental versus clinical studies capture distinct types of relapse in tuberculosis
Source: mBio. 2026 Sep 18;17(10):e01977-26. doi: 10.1128/mbio.01977-26 (PMC13712832; doi:10.1128/mbio.01977-26)
Supplement: Supplementary file 1 [file mbio.01977-26-s0001.pdf]

File SA: Extended background and details of new model components. This supplement contains additional details and context necessary to perform this work (SA1) Extended TB background, including details of existing diagnostic tests. (SA2) Formulation and calibration of our submodel of stochastic release of Mtb from caseum. (SA3) Details of virtual HIV-1/SIV co-infection. (SA4) Methodology for performing species-specific disease state classification. (SA5) Updates to our model of Mtb dissemination. (SA6) Repeated reservoir-threshold analysis using RMZE.

SA1 - Extended Tuberculosis background

SA1.1 - Granulomas

To understand observed variability in responses to TB treatment, it is necessary to consider the immunology and heterogeneity of *lung granulomas* that arise during Mtb infection. Granulomas are spatially-complex structures that both physically and immunologically quarantine Mtb[1]. Most often, granulomas, feature an outer region containing multiple innate and adaptive immune cells and bacteria and a necrotic, hypoxic region filled with *caseum* - an aggregate of dead cells, cellular debris, and non-replicating Mtb[1]. Both granulomas and caseum within them are a primary pharmacokinetic and pharmacodynamic barrier to TB treatment[2], as antibiotics do not uniformly penetrate granulomas or into caseum and are not uniformly effective at killing Mtb across metabolic states. Finally, wide variations of individual granuloma outcomes are observed within individual Mtb-infected hosts[3].

SA1.2 - Combined positron emission tomography (PET) and computed tomography (CT)

PET/CT measures the metabolism of the radiolabeled 18F-fluorodeoxyglucose (FDG) as a measure of inflammation[4,5]. FDG avidity, measured from PET/CT, is known to correlate with CFU levels in NHPs[6]. It was found that many clinically-cured patients have ongoing inflammation and detectable Mtb mRNA post-treatment[7], yet inflammation reduction has been noted during antibiotic treatment in mice[8,9] and NHPs[10].

SA1.3 - Diagnostic tests for clinical TB

Multiple diagnostic tests have been used to diagnose TB, assess for subclinical disease, and experimentally assess animals or *ex vivo* tissues for Mtb. In Table SA1 we provide an overview of many common methods, based in part on a previous review[9].

Table SA1: Common methods for assessing *M. tuberculosis* infection and/or TB disease *in vivo*, based in part on [9].

Diagnostic	Advantages	Drawbacks	CFU Level of Detection (LOD)	References
Solid or liquid culture of BAL	Gold standard for positive diagnosis when repeated	Can take weeks to months, requires 2+ tests. Poor predictor of relapse during early infection	1 CFU within BALF or sputum (ideally)	9,11,12
WHO-approved rapid diagnostics (e.g., Xpert ultra)	Fast, accurate	Expensive, can bear false positive after previous infection	16 CFU/mL (BALF or sputum)	13,14
Acid-fast sputum or BALF smear	Fast, inexpensive	Yields false positives after previous infection, false-positive rate may depend on treatment regimen	~5,000 CFU/mL (BALF or sputum)	9,15,16
Whole-lung or tissue homogenate	Highly accurate, detects infections during active TB or subclinical disease	Animal model only (Performed post-necropsy)	~5CFU/sample	17(NHP); 18,19(Mouse)
Clinical radiographic tests (Chest X rays / Chest CT)	Non-invasive, fast, widely available, used to detect evidence of progressive TB	Does not quantify or assess inflammation levels; unknown precise Mtb LOD; poor specificity; reader inconsistency.	Varies	20,21
Other radiographic tests (e.g., checking for dissemination using a 18F-fluorodeoxyglucose radio-tagged PET/CT probe)	Non-invasive, spatially resolves individual granuloma disseminations, correlates with Mtb burden, longitudinal data available	Measures gross fibrosis, cavitation, or inflammation; typically associated with disease severity or bacterial burden but can be host response, which may persist post-cure; unknown precise Mtb LOD	- Not a direct measure of CFU, but FDG avidity from PET/CT is proportional to CFU[6].<br><br>(Returns a measure of inflammation in host)	6,17 (NHP); 4,20(Human)
Immune assays	Some, like tuberculin skin tests (TSTs) or interferon gamma release assays (IGRAs), only measure Mtb infection. Standard	Unclear as to how long inflammation lasts after Mtb are cleared, not always able to distinguish	- Not based on CFU<br><br>(Returns a positive/negative based on sufficient presence/absence of TB-associated immune markers)	22–25

	diagnostic tests include TSTs and QuantiFERON-TB Gold In-Tube (an IGRA).	active TB from subclinial disease<br>Low quality evidence for the ability to detect subclinical disease		
Recording clinical TB indicators: New prolonged cough (≥14 days), weight loss, etc.	Noninvasive. Motivates the patient / care team to test for TB that may be otherwise missed	Insufficient to diagnose TB by themselves	Unclear connection to CFU<br><br>(Gives clinical picture not linked to infection measures)	[26]

SA2 - Detailed Reservoir Mechanism of Relapse

SA2.1 - Reservoir Model of relapse description

The ODE components of our hybrid ODE/agent-based model yield continuous trajectories of cell populations, including both caseum-bound bacteria B_N and intracellular bacteria B_I. We capture reservoir-driven relapse by using a stochastic impulse into our system, called *release events*. When a release event occurs, we move one unit from non-replicating bacteria trapped within caseum, B_N and add one unit to the intracellular bacteria population outside of caseum, B_I. We remove one unit from resting macrophages and add one to the number of infected macrophages, representing macrophages internalizing that single, trafficked bacterium. We allow for a release event to occur at most once-per-day occurring within each granuloma using the following probability:

$$Prob(\text{Release Event}) = \frac{{B_N}^2}{{B_N}^2 + {C_{N,\text{release}}}^2} \cdot \left(1 - \frac{[\sum \text{T}]^2}{[\sum \text{T}]^2 + {C_{T,\text{release}}}^2}\right)$$

where B_N is the population size of non-replicating bacteria trapped in caseum, $\sum T$ is the total number of Interleukin-10 producing T-cell species in our simulation (as reductions of such T cells are associated with higher rates of reactivation[27] and other presentations of Mtb dissemination[28]). The parameters $C_{N,\text{release}}$ and $C_{T,\text{release}}$ are phenomenological parameters to calibrate.

SA2.2 - Calibration of stochastic release mechanism using anti-TNFα immunosuppression

Reactivation occurs both in humans and NHPs and is inducible by TNFα-neutralizing and anti-TNFα therapy[29–36]. Anti-TNF medications such as infliximab, etanercept, and adalimumab either bind TNF or inhibit its production. Not all mechanisms of TNF depletion have the same impact on TB outcomes *in vivo*; reactivation of subclinical TB is roughly five times more likely when taking infliximab (a monoclonal TNF antibody) versus etanercept (a fusion protein of TNF)[36–39]. In *HostSim*, we coarse-grain these mechanisms together, and achieve the drug effects by zeroing-out all production terms to TNF—most similar to the action of monoclonal antibodies such as infliximab.

Previous studies have observed 70-80% reactivation of subclinical TB in NHPs when TNFα is neutralized by either Adalimumab (8 weeks) or p55-TNFRI (19 weeks)[31,32], though this reactivation can be slowed by a combination treatment of Isoniazid and Rifampicin or with Metronidazole[31]. Approximately half of a cohort of NHPs with depleted TNF showed new granulomas within 8 weeks[30]. In humans, the precise likelihood of reactivation with TNF suppression is difficult to estimate because the size of the non-reactivating population is unknown. However, a hidden Markov model in 2004 estimated that approximately 20% of humans with subclinical disease would reactivate each month[33]. We also know that out of the patients who experience relapse consequent to treatment with Infliximab, around 44% relapse within 90 days of receiving treatment[40], although other anti-TNF drugs such as Etanercept and Adalimumab report lower levels of reactivation[36,38–40].

Beyond CFU, dissemination has also been noted in TNF-driven reactivation. In NHP experiments, reactive disease has been measured by the presence of new disseminations[30] and, in that study, 50% of NHPs with subclinical TB and had anti-TNF treatment, experienced reactive disease. Elsewhere, it was reported more than half of NHPs treated with TNF-neutralizing drugs exhibited signs of reactivation[32], and another study showed up to 70% reactivation[31,32]. We calibrate our model to have more than half of TB-controlling hosts result in relapse upon depletion of TNF for eight weeks.

We calibrate our virtual stochastic release mechanism (i.e., $C_{N,\text{release}}$ and $C_{T,\text{release}}$) by recreating these expected trends in TNF-depletion studies, notably to have reactivation rates between 70% to 80%. As a coarse-grained model for predicting symptomatic TB, we assume that fold-increase of CFU between 2x and 10x in lung tissue

is indicative of symptoms. We simulate Mtb infection progression in a cohort of 500 virtual hosts for 365 days. At day 365, we virtually suppress TNF in all hosts by setting TNF production terms to 0 for a period of 8 weeks. We then measured the portion of previously-subclinical hosts that became clinically-infected. We manually calibrated our parameters to $C_{N,\text{release}} = 10,\ C_{T,\text{release}} = 300$, which results in 74.4% (88.8%) of subclinical hosts experiencing at least a 10-fold (2-fold) increase of CFU or new disseminations.

SA2.2 - Further validation with virtual IFNγ depletion

Another immune cytokine that serves as a validation target is IFNγ, a pro-inflammatory cytokine that is produced by T cells and is a key player of one pathway by which the immune system regulates PD-1 / PD-L1[41]. The authors found few studies of IFNγ depletion experiments during Mtb treatment, an experiment in the 90s showed that IFNγ knockout mice suffer rapid and fatal Mtb infections[42], although more specialized mouse models may further verify these results (e.g., the C3H/HeJ and C3HeB/FeJ "Kramnik" mice that form necrotic lesions[18]). There have been case studies of humans being treated for cancer with PD-1 inhibitors that subsequently developed active tuberculosis[43]. The IFNγ blocker Emapalumab (AKA Gamifant), used to treat hemophagocytic lymphohistiocytosis, requires screening for subclinical TB infection and prophylactic treatment for subclinical TB[44].

For further validation, we performed a virtual IFNγ depletion experiment similar to our TNFα depletion experiment above, assuming that all new IFNγ production is halted. After eight weeks of IFNγ depletion, we find that 50% of virtual hosts experience at least a 2-fold increase of CFU or new disseminations, and 0.5% of subclinical hosts experience at least a 10-fold increase of CFU or new disseminations.

SA3 - Virtual HIV-1/SIV infection

Coinfection with both HIV and TB is one of the most well-known and dangerous disease outcomes[22,45]. Infection with HIV causes, among other effects, a near-complete depletion of CD4+ effector T cells[46]. HIV infects and kills different immune subpopulations preferentially - notably the CCR5+ memory cell population[46]. We assume HIV-1 and SIV virtual T-cell depletion behaves similarly[47–50]. Note that (1) HIV-1 and SIV are not identical - e.g., SIV rarely causing AIDS-like symptoms in natural hosts[47]. and (2) there is evidence that SIV-induced relapse is not identical to CD4+ T-cell depletion-induced relapse[48].

In *HostSim,* we capture co-infection with HIV via a killing term to all memory and effector cell populations. Specifically, we represent virtual HIV infection at time t as an additional death term that is 0 prior to t and at the time of infection, and then linearly increases to a value of k_{HIV} at time $t + t_{onset}$. That is, for all CD4+ effector and memory T-cell populations, we impose

$$\frac{d}{dt}T = \cdots - \underbrace{(\Psi \cdot k_{HIV}T)}_{\text{HIV loss}}$$

where Ψ is a continuous and piecewise linear scaling term that is 0.25 prior to t, 1 after $t + t_{onset}$, and linearly connected between those two times. We virtually infect our hosts with HIV twelve months after the end of TB treatment and assume an onset time of $t_{onset} = 2$ years. The HIV loss term introduces a new steady-state that the system reaches on a time-scale faster than the linear decrease itself, meaning that by slowly adjusting Ψ, we also slowly adjust the T-cell concentration as in Figure SA1. A roughly linear drop of between 20-50% over two years was reported in the 1990s[51], and we achieve this after adapting published parameters of an older model[52] $k_{HIV} = 0.0082$.

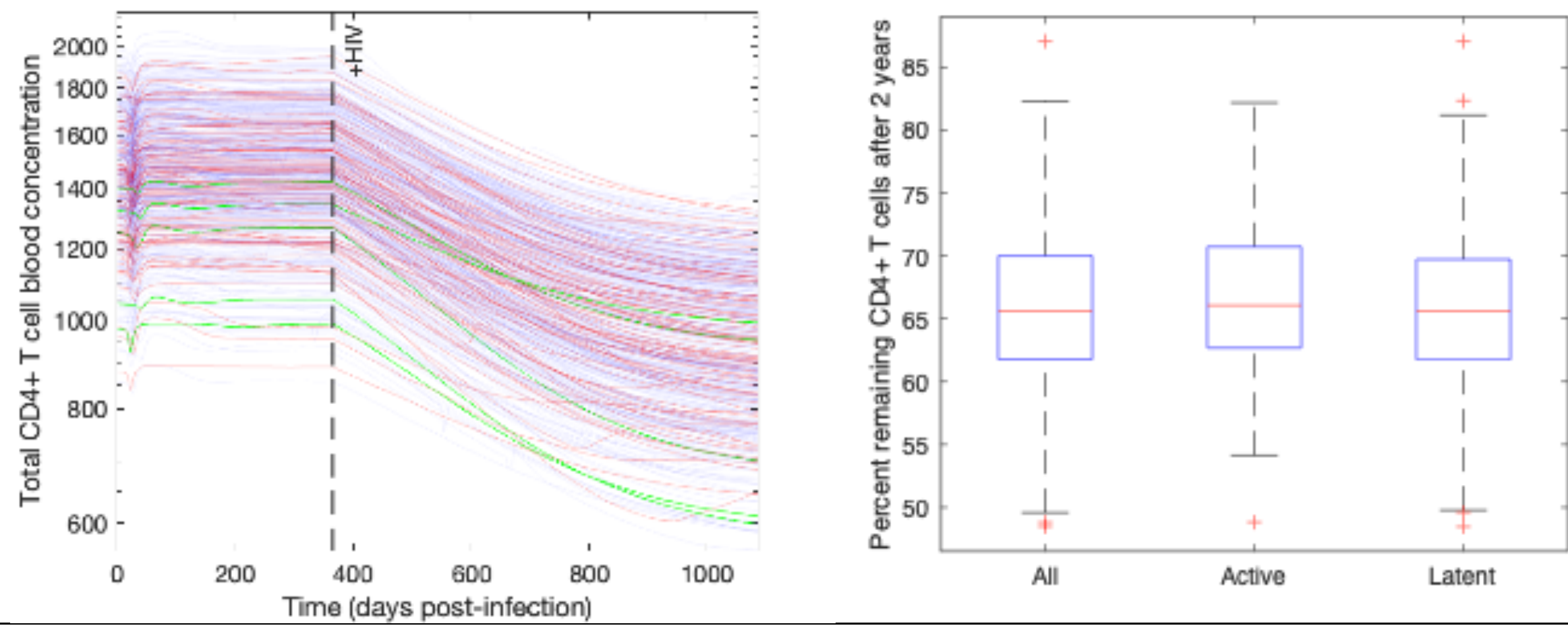

Figure SA1: Simulated CD4+ T-cell depletion during virtual HIV/SIV infection. (Left) Blood concentration of CD4+ T cells during the first 2 years of virtual HIV infection. (Right) Percent remaining CD4+ T cells in the virtual blood compartment after 2 years of simulated HIV infection, showing a trajectory similar to that in literature[51].

SA4 - Bacterial loads versus disease state prediction method

As described in the main text, we coarse-grain our prediction of virtual host disease state by the fold-increase of CFU in that virtual host. Each virtual host has a predicted CFU trajectory based on model parameters. Parameters are in **File SB**, and discussed in detail in our previous work[53] as well as our website at
http://malthus.micro.med.umich.edu/lab/supplements/Host-Sim-3/.

Without an accurate symptom-prediction model, we use estimate a virtual host's disease state based purely on lung bacterial load. If CFU levels increase by R-fold levels between days 100 (post-transient-peak) and 300 (mature infection), then we define uncontrolled infection as symptomatic disease. In *HostSim*, we use $R = 10$ to capture the 90% subclinical / 10% active TB distributions observed in human studies for decades[23,30,54,55]. However, lowering $R = 1.25$ redefines the classification, bringing the percentage active TB to 25%, closer to the 1:1 observed in cynomolgus macaques[56]. Figure SA2 shows CFU counts for a single set of 500 virtual hosts, illustrating which trajectories are classified as active with NHP-like or human-like classification of active TB disease. We assume $R = 1.01$ for mice, as we assume that without confining Mtb to granulomas, any increase of bacterial burden over time is indicative of poor control.

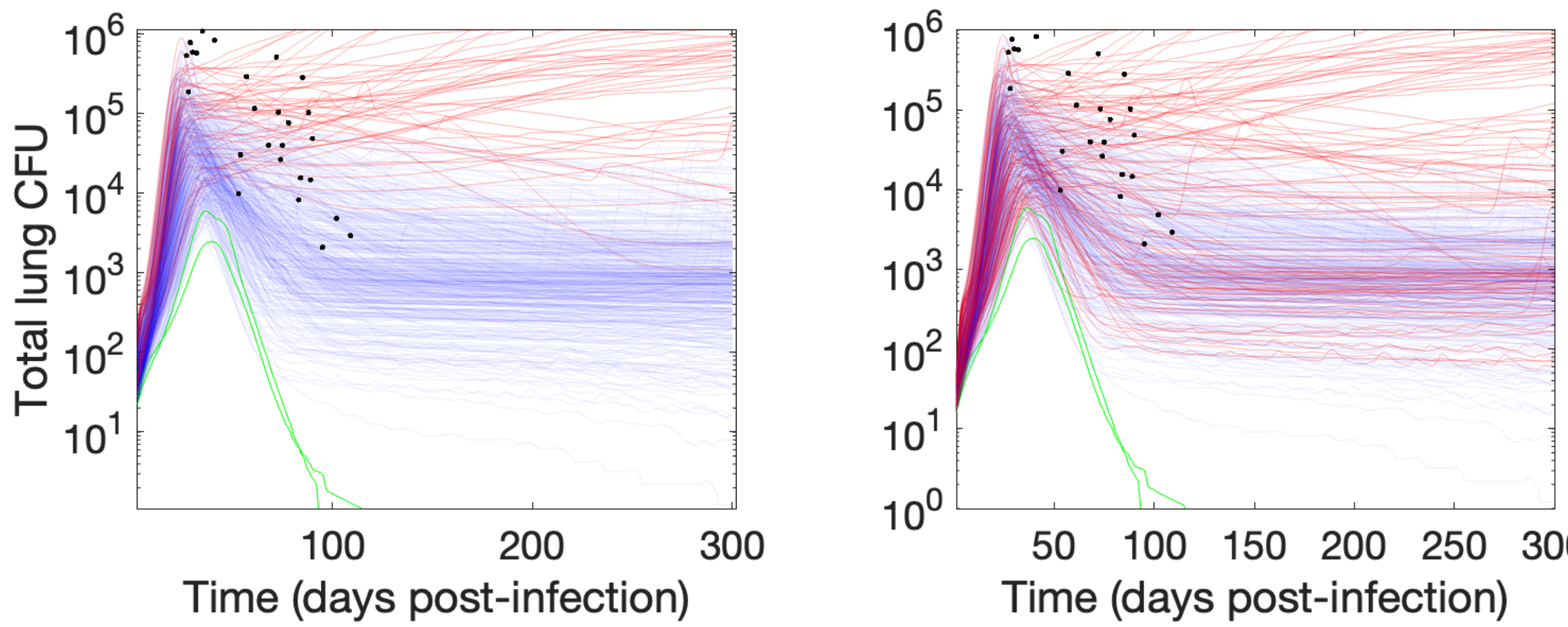

Figure SA2: Simulated CFU levels colored by predicted virtual host state. Both panels show the same CFU trajectories of the same 500 virtual hosts based on human-like active-TB classification ($R = 10$, left) or NHP-like active-TB classification ($R = 1.25$, right). When hosts are classified at 300 days post-infection, we predict either 10% active hosts (human-like) or 25% active hosts (NHP-like).

SA5 - Dissemination

If Mtb disseminate, they can found new granulomas elsewhere in the lung[1]. This has been used as an indicator of progressive infection, and we assume that within-lung dissemination occurs in *HostSim*. We represent these as discrete events wherein a new granuloma is created within a virtual host, as in previous work with *HostSim*[53,57,58]. Disseminations in *HostSim* can be local (spatially proximal to the original infection site) or non-local (distal, possibly in other lung lobes). There are two mechanisms that contribute to a per-day probability of granuloma dissemination in *HostSim*.

SA4.1 - CFU-threshold based dissemination

Granulomas that maintaining very high CFU levels are likely to disseminate. We assume that granulomas with CFU > $\approx 10^6$ are not well controlled and likely to progress in infection. This is measured as a per-day probability of a granuloma disseminating as

$$P(dissemination) = \lambda \cdot \frac{\text{CFU}}{\text{CFU} + 10^5},$$

where $\lambda \in [0.02,0.04]$ for local dissemination and $\lambda \in [0.002,0.004]$ for non-local dissemination, as calibrated previously[53].

SA4.2 - Uncontrolled growth of Mtb

We assume that continued increase of CFU over time after the granuloma is matured (e.g., a constant increase from 100CFU to 500CFU in a years-old granuloma) is a marker for risk of dissemination. Our base estimates root from the following observations.

First is that hosts with subclinical TB do not disseminate frequently. As part of the dissemination of Mtb during subclinical disease, we assume that we should expect perhaps 1 dissemination over many years. Secondly, we estimate that if CFU in a granuloma increases by >5% CFU per day, approximately 1 dissemination occurs in a month. Third, we assume that immunosuppression induces more dissemination.

We compute the probability of dissemination from this mechanism from a Weibull distribution, where

$$P(dissemination) = 1 - \exp\left(-\left(\Delta t/(L)\right)^{K(q)}\right)$$

where

$$q = \frac{(\text{previous CFU})}{(\text{current CFU})}$$

and

$$K(q) = K_{Stable} + (K_{Unstable} - K_{Stable}) \cdot \frac{(q'-1)^2}{(q'-1)^2 + 0.02^2};\ q' = \max(1, q),$$

and the parameters are $K_{stable} = 4.38$ and $K_{unstable} = 0.75$ for local dissemination, and $K_{stable} = 4.74$ and $K_{unstable} = 1.11$ for non-local dissemination. We chose the Weibull scale parameter L, to a characteristic time-scale for dissemination. We estimated $L = 90$ days as (i) dissemination events are a marker for reactivation[30], and (ii) between 50% and 75% of infliximab-induced reactivation occurs within 90 days, observed in both humans[33] and simulations[40]. For local dissemination, we compute these parameters directly from the system of equations emergent from our assumptions: $E\left(P(Dissemination\ in\ 1\ year\ with\ q = 1)\right) \approx 10^{-6}$ and $E\left(P(Dissemination\ in\ 30\ days\ with\ q = 1/1.05)\right) \approx 1$. Values for non-local dissemination are calculated with one-fifth the expected probability.

SA6 - Repeated reservoir-threshold analysis using RMZE

We examine simulated rates of relapse 1 year after administering a 2-month short-course treatment of RMZE to 500 virtual hosts. We test for Mtb both at time of treatment completion and one year later by using *Virtual Mtb plate* (Methods), varying the LOD of both initial and follow-up tests from 0 to 100 CFU. We also vary whether or not these diagnostics can detect non-replicating CFU. Qualitative results are identical to those shown in HRZE (Figure SA3), with the exception of a single instance of threshold-driven relapse occurring at lower LOD in virtual tests that cannot detect non-replicating bacteria.

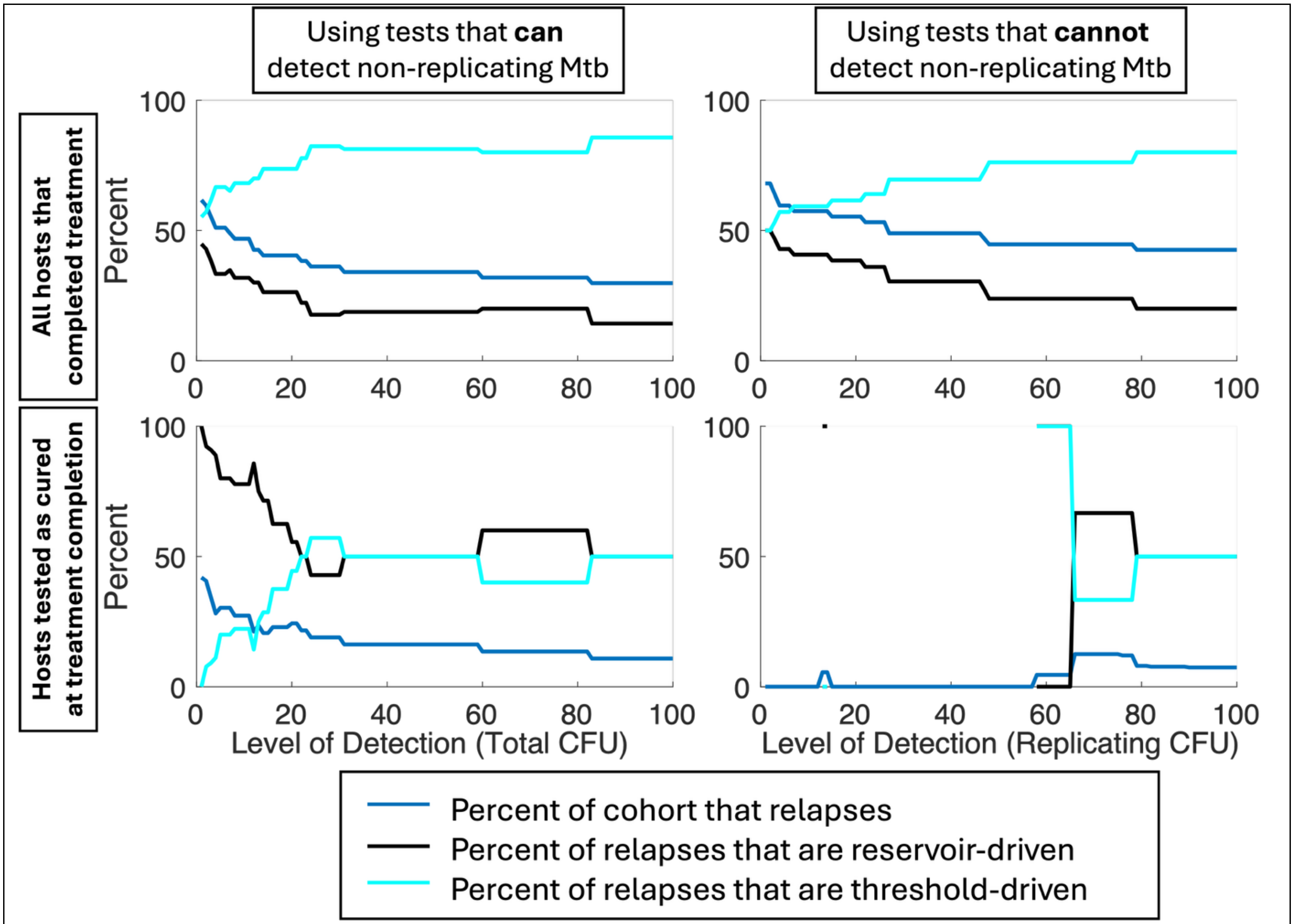

Figure SA3: Reservoir versus threshold relapse rates by test LOD. Each of our plots show how simulated relapse rates and the relative frequency of their driving mechanisms are impacted by LOD of the diagnostic test used to assess cure and/or relapse. These analyses used the *Virtual Mtb plate* test to mimic sputum culture conversion tests one year after active TB hosts complete 2 months of virtual RMZE treatment (Methods). We repeated each analysis assuming the *Virtual Mtb plate* either can (Left column) or cannot (Right column) detect non-replicating bacteria. We also repeated each analysis varying whether relapse is defined of all hosts that complete treatment (Top row) or only those that are misdiagnosed as "cured" upon treatment completion (Bottom row).

File SA References

1. Ramakrishnan, L. Revisiting the role of the granuloma in tuberculosis. *Nat Rev Immunol* **12**, 352–66 (2012).
2. Sarathy, J. P. *et al.* Prediction of Drug Penetration in Tuberculosis Lesions. *ACS Infect Dis* **2**, 552–63 (2016).
3. Lin, P. L. *et al.* Sterilization of granulomas is common in active and latent tuberculosis despite within-host variability in bacterial killing. *Nat Med* **20**, 75–9 (2014).
4. Chen, R. Y. *et al.* PET/CT imaging correlates with treatment outcome in patients with multidrug-resistant tuberculosis. *Sci. Transl. Med.* **6**, 265ra166-265ra166 (2014).
5. Lin, P. L. *et al.* Radiologic responses in cynomolgous macaques for assessing tuberculosis chemotherapy regimens. *Antimicrob Agents Chemother* https://doi.org/10.1128/AAC.00277-13 (2013) doi:10.1128/AAC.00277-13.
6. Maiello, P. *et al.* Rhesus Macaques Are More Susceptible to Progressive Tuberculosis than Cynomolgus Macaques: a Quantitative Comparison. *Infect Immun* **86**, (2018).
7. Malherbe, S. T. *et al.* Persisting positron emission tomography lesion activity and Mycobacterium tuberculosis mRNA after tuberculosis cure. *Nat. Med.* **22**, 1094–1100 (2016).
8. Ordonez, A. A. *et al.* Radioiodinated DPA-713 Imaging Correlates with Bactericidal Activity of Tuberculosis Treatments in Mice. *Antimicrob. Agents Chemother.* **59**, 642–649 (2015).
9. Goletti, D. *et al.* Can we predict tuberculosis cure? What tools are available? *Eur. Respir. J.* **52**, (2018).
10. Budak, M. *et al.* Optimizing tuberculosis treatment efficacy: Comparing the standard regimen with Moxifloxacin-containing regimens. *PLoS Comput. Biol.* **19**, e1010823 (2023).
11. Johnson, J. L. *et al.* Shortening treatment in adults with noncavitary tuberculosis and 2-month culture conversion. *Am J Respir Crit Care Med* **180**, 558–63 (2009).
12. Lu, P. *et al.* Time to sputum culture conversion and treatment outcome of patients with multidrug-resistant tuberculosis: a prospective cohort study from urban China. *Eur. Respir. J.* **49**, (2017).
13. Dorman, S. E. *et al.* Xpert MTB/RIF Ultra for detection of Mycobacterium tuberculosis and rifampicin resistance: a prospective multicentre diagnostic accuracy study. *Lancet Infect. Dis.* **18**, 76–84 (2018).
14. Steingart, K. R. *et al.* Xpert® MTB/RIF assay for pulmonary tuberculosis and rifampicin resistance in adults - Steingart, KR - 2014 | Cochrane Library. https://www.cochranelibrary.com/cdsr/doi/10.1002/14651858.CD009593.pub3/information.
15. 9.3 Sputum smear and culture | TB Knowledge Sharing. https://tbksp.who.int/en/node/1970.
16. Chen, W.-C., Chang, C.-C. & Lin, Y. E. Pulmonary Tuberculosis Diagnosis Using an Intelligent Microscopy Scanner and Image Recognition Model for Improved Acid-Fast Bacilli Detection in Smears. *Microorganisms* **12**, 1734 (2024).
17. Maiello, P. *et al.* Characterizing PET CT patterns and bacterial dissemination features of tuberculosis relapse in the macaque model. *Infect. Immun.* **93**, e00177-25 (2025).
18. Kramnik, I. & Beamer, G. Mouse models of human TB pathology: roles in the analysis of necrosis and the development of host-directed therapies. *Semin. Immunopathol.* **38**, 221–237 (2016).
19. Ibrahim, M., Truffot-Pernot, C., Andries, K., Jarlier, V. & Veziris, N. Sterilizing Activity of R207910 (TMC207)-containing Regimens in the Murine Model of Tuberculosis. *Am. J. Respir. Crit. Care Med.* **180**, 553–557 (2009).
20. Skoura, E., Zumla, A. & Bomanji, J. Imaging in tuberculosis. *Int. J. Infect. Dis.* **32**, 87–93 (2015).
21. Pinto, L. M. *et al.* Scoring systems using chest radiographic features for the diagnosis of pulmonary tuberculosis in adults: a systematic review. *Eur. Respir. J.* **42**, 480–494 (2013).
22. *Latent Tuberculosis Infection: Updated and Consolidated Guidelines for Programmatic Management*. (World Health Organization, Global TB Programme, Geneva, Switzerland, 2018).
23. Selvavinayagam, S. T. *et al.* Clinical laboratory analytes and platelet-associated parameters as surrogate markers of subclinical inflammation in latent tuberculosis infection. *Front. Immunol.* **16**, (2025).
24. Goletti, D. *et al.* Accuracy of Immunodiagnostic Tests for Active Tuberculosis Using Single and Combined Results: A Multicenter TBNET-Study. *PLOS ONE* **3**, e3417 (2008).

25. Zhang, H. *et al.* Reversion of QuantiFERON-TB Gold In-Tube test in individuals with and without prophylactic treatment for latent tuberculosis infection: A systematic review and meta-analysis. *J. Infect.* **77**, 276–282 (2018).
26. *WHO Consolidated Guidelines on Tuberculosis. Module 4: Treatment - Drug-Resistant Tuberculosis Treatment, 2022 Update*. (World Health Organization, Geneva, 2022).
27. Lin, P. L. *et al.* CD4 T cell depletion exacerbates acute Mycobacterium tuberculosis while reactivation of latent infection is dependent on severity of tissue depletion in cynomolgus macaques. *AIDS Res Hum Retroviruses* **28**, 1693–702 (2012).
28. Winchell, C. G. *et al.* CD8+ lymphocytes are critical for early control of tuberculosis in macaques. *J Exp Med* **220**, (2023).
29. Flynn, J. L. & Chan, J. Tuberculosis: latency and reactivation. *Infect Immun* **69**, 4195–201 (2001).
30. Lin, P. L. *et al.* PET CT Identifies Reactivation Risk in Cynomolgus Macaques with Latent M. tuberculosis. *PLoS Pathog* **12**, e1005739 (2016).
31. Lin, P. L. *et al.* Metronidazole prevents reactivation of latent Mycobacterium tuberculosis infection in macaques. *Proc Natl Acad Sci U A* **109**, 14188–93 (2012).
32. Lin, P. L. *et al.* Tumor necrosis factor neutralization results in disseminated disease in acute and latent Mycobacterium tuberculosis infection with normal granuloma structure in a cynomolgus macaque model. *Arthritis Rheum* **62**, 340–50 (2010).
33. Wallis, R. S., Broder, M. S., Wong, J. Y., Hanson, M. E. & Beenhouwer, D. O. Granulomatous infectious diseases associated with tumor necrosis factor antagonists. *Clin Infect Dis* **38**, 1261–5 (2004).
34. Tubach, F. *et al.* Risk of tuberculosis is higher with anti–tumor necrosis factor monoclonal antibody therapy than with soluble tumor necrosis factor receptor therapy: The three-year prospective french research axed on tolerance of biotherapies registry. *Arthritis Rheum.* **60**, 1884–1894 (2009).
35. Fallahi-Sichani, M., Schaller, M. A., Kirschner, D. E., Kunkel, S. L. & Linderman, J. J. Identification of key processes that control tumor necrosis factor availability in a tuberculosis granuloma. *PLoS Comput Biol* **6**, e1000778 (2010).
36. Fallahi-Sichani, M., Flynn, J. L., Linderman, J. J. & Kirschner, D. E. Differential risk of tuberculosis reactivation among anti-TNF therapies is due to drug binding kinetics and permeability. *J Immunol* **188**, 3169–78 (2012).
37. Robert, M. & Miossec, P. Reactivation of latent tuberculosis with TNF inhibitors: critical role of the beta 2 chain of the IL-12 receptor. *Cell. Mol. Immunol.* **18**, 1644–1651 (2021).
38. Wallis, R. S. Infectious complications of tumor necrosis factor blockade. *Curr. Opin. Infect. Dis.* **22**, 403 (2009).
39. Dixon, W. G. *et al.* Drug-specific risk of tuberculosis in patients with rheumatoid arthritis treated with anti-TNF therapy: results from the British Society for Rheumatology Biologics Register (BSRBR). *Ann. Rheum. Dis.* **69**, 522–528 (2010).
40. Wallis, R. S. Mathematical modeling of the cause of tuberculosis during tumor necrosis factor blockade. *Arthritis Rheum* **58**, 947–52 (2008).
41. Han, Y., Liu, D. & Li, L. PD-1/PD-L1 pathway: current researches in cancer. *Am. J. Cancer Res.* **10**, 727–742 (2020).
42. Flynn, J. L. *et al.* An essential role for interferon gamma in resistance to Mycobacterium tuberculosis infection. *J Exp Med* **178**, 2249–54 (1993).
43. Anastasopoulou, A., Ziogas, D. C., Samarkos, M., Kirkwood, J. M. & Gogas, H. Reactivation of tuberculosis in cancer patients following administration of immune checkpoint inhibitors: current evidence and clinical practice recommendations. *J. Immunother. Cancer* **7**, 239 (2019).
44. Gamifant-clinical-overview.pdf. https://gamifant.com/pdf/clinical-overview.pdf.
45. Yilma, A., Bailey, H., Karakousis, P. C. & Karanika, S. HIV/Tuberculosis Coinfection in Pregnancy and the Postpartum Period. *J. Clin. Med.* **12**, 6302 (2023).

46. Le Hingrat, Q., Sereti, I., Landay, A. L., Pandrea, I. & Apetrei, C. The Hitchhiker Guide to CD4+ T-Cell Depletion in Lentiviral Infection. A Critical Review of the Dynamics of the CD4+ T Cells in SIV and HIV Infection. *Front. Immunol.* **12**, (2021).
47. Raehtz, K., Pandrea, I. & Apetrei, C. The well-tempered SIV infection: Pathogenesis of SIV infection in natural hosts in the wild, with emphasis on virus transmission and early events post-infection that may contribute to protection from disease progression. *Infect. Genet. Evol.* **46**, 308–323 (2016).
48. Diedrich, C. R. *et al.* SIV and Mycobacterium tuberculosis synergy within the granuloma accelerates the reactivation pattern of latent tuberculosis. *PLOS Pathog.* **16**, e1008413 (2020).
49. Diedrich, C. R. *et al.* Reactivation of latent tuberculosis in cynomolgus macaques infected with SIV is associated with early peripheral T cell depletion and not virus load. *PLoS One* **5**, e9611 (2010).
50. Burdo, T. H., Walker, J. & Williams, K. C. Macrophage Polarization in AIDS: Dynamic Interface between Anti-Viral and Anti-Inflammatory Macrophages during Acute and Chronic Infection. *J. Clin. Cell. Immunol.* **6**, 333 (2015).
51. Kirschner, D. & Webb, G. F. Immunotherapy of HIV-1 Infection. (1997).
52. Kirschner, D. Dynamics of co-infection with M. Tuberculosis and HIV-1. *Theor Popul Biol* **55**, 94–109 (1999).
53. Michael, C. T. *et al.* Rankings of tuberculosis antibiotic treatment regimens are sensitive to spatial scale, detection limit, and initial host bacterial burden. *J. Theor. Biol.* **611**, 112176 (2025).
54. Cadena, A. M., Fortune, S. M. & Flynn, J. L. Heterogeneity in tuberculosis. *Nat. Rev. Immunol.* **17**, 691–702 (2017).
55. Lin, P. L. & Flynn, J. L. The End of the Binary Era: Revisiting the Spectrum of Tuberculosis. *J Immunol* **201**, 2541–2548 (2018).
56. Capuano, S. V. *et al.* Experimental Mycobacterium tuberculosis infection of cynomolgus macaques closely resembles the various manifestations of human M. tuberculosis infection. *Infect Immun* **71**, 5831–44 (2003).
57. Joslyn, L. R., Linderman, J. J. & Kirschner, D. E. A virtual host model of Mycobacterium tuberculosis infection identifies early immune events as predictive of infection outcomes. *J. Theor. Biol.* **539**, 111042 (2022).
58. Michael, C. T., Almohri, S. A., Linderman, J. J. & Kirschner, D. E. A framework for multi-scale intervention modeling: virtual cohorts, virtual clinical trials, and model-to-model comparisons. *Front. Syst. Biol.* **3**, (2024).

File SB: HostSim technical description. This supplement contains all of the model equations and parameter values used for the current version of HostSim. This includes (SB1) our granuloma ODE, (SB2) our lymph node and blood coupled ODE, (SB3) our method for coupling granulomas with lymph nodes and blood, (SB4) how we implement Mtb dissemination and formation of new granulomas, (SB5) our model of antibiotic treatment, and (SB6) our parameter values.

Overview

This document contains the equations for each compartment in *HostSim*, how they are coupled, parameter ranges, and initial conditions. *HostSim* was originally published by Louis Joslyn, Jennifer Linderman, and Denise Kirschner in the article "A virtual host model of Mycobacterium tuberculosis infection identifies early immune events as predictive of infection outcomes" in 2022 in the *Journal of Theoretical Biology*.

This technical description contains reproductions of the algorithms, equations, and supplemental materials presented in previous publications, including:

- L. Joslyn et al. "A virtual host model of Mycobacterium tuberculosis infection identifies early immune events as predictive of infection outcomes". *The Journal of Theoretical Biology.* 2022.
- C.T. Michael et al. "A framework for multi-scale intervention modeling: virtual cohorts, virtual clinical trials, and model-to-model comparisons" in *Frontiers in Systems Biology*. 2024.
- C.T. Michael et al. "Rankings of tuberculosis antibiotic treatment regimens are sensitive to spatial scale, detection limit, and initial host bacterial burden". *The Journal of Theoretical Biology.* 2025.

We have separated this document into sections for readability. In Section B1, we detail all of the equations in each granuloma compartment. In Section B2, we detail the equations in the lymph node and blood compartments. In Section B3, we present the method by which all of our compartments are coupled. In Section B4, we describe how we model dissemination - that is, how we represent one granuloma causing another to form. In Section B5, we include the entirety of our drug computation process. In Section B6, we present tables for the parameter ranges and initial condition values for our granuloma, lymph node and blood, and pharmacokinetic model components. In each of these sections, we included both a symbolic variable for each variable (e.g., M_R or ξ_2) and a corresponding non-symbolic variable (e.g., MR or xi2) for convenience of navigating this document via text search.

SB1: Granuloma equations

Each granuloma has its own distinctly-parameterized instance of the equations presented in the subsections below. Each host has a "host-scale granuloma parameter base" selected by the LHS sampling scheme using the ranges presented in Section B5.1. To capture intra-host variability, individual granulomas within a host are given parameter values normally distributed around this base host value. Terms indicated by $Pulled\left[\cdot\right]_g$ involve quantities pulled from the lymph node and blood equations, and is described in Section B3. Table SB1 provides an index of all state variables in granulomas.

Table SB1: **Granuloma equation state variables.**

State Variable	Non-Symbolic	Units	Description
M_R	MR	count	Resting macrophages
M_I	MI	count	Infected macrophages
M_A	MA	count	Activated macrophages
B_I	BI	count	Intracellular Mtb
B_E	BE	count	Extracellular Mtb
B_N	BN	count	Non-replicating Mtb
Ca	CA	mass of $M\phi$	Necrotic tissue mass
Ag	AG	mass Ag/Mtb	Antigen mass within granulomas
T_0^4	T40	count	Mtb-specific primed CD4$^+$ T-cells
T_1^4	Th1	count	Mtb-specific Th1 CD4$^+$ T-cells
T_2^4	Th2	count	Mtb-specific Th2 CD4$^+$ T-cells
T_{Non}^4	CD4Non	count	Nonspecific CD4$^+$ T-cells
T_{EM}^4	EMCD4	count	Mtb-specific CD4$^+$ effector memory T-cells
T_0^8	T80	count	Mtb-specific primed CD8$^+$ T-cells
T_C	TC	count	Mtb-specific cytotoxic CD8$^+$ T-cells
T^8	T8	count	Mtb-specific effector CD8$^+$ T-cells
T_{EM}^8	EMCD8	count	Mtb-specific CD8$^+$ effector memory T-cells
T_{Non}^8	CD8Non	count	Nonspecific CD8$^+$ T-cells
F_α	TNF	pg/mL	TNF-α concentration
I_γ	IG	pg/mL	IFN-γ concentration
I_{12}	I12	pg/mL	Interleukin 12 concentration
I_{10}	I10	pg/mL	Interleukin 10 concentration
I_4	I4	pg/mL	Interleukin 4 concentration

SB1.1 Macrophage equations

M_R - MR - Resting macrophage count

$$\frac{d}{dt}(M_R) = \underbrace{\alpha_{4a}\left(M_A + w_2 M_I\right)}_{\text{Macrophage-driven recruitment}} + \underbrace{Sr_{4b}\left(\frac{F_\alpha}{F_\alpha + f_8 I_{10} + s_{4b}}\right)}_{\text{TNF driven recruitment}} + \underbrace{\psi_C \psi_S \left(\sum_{j=1}^{n} R_{IC/EC\text{ for drug j}}^{FIC}\right)^{1/FIC} k_I(C_{eff}) M_I}_{\text{Drug-induced reversion from } M_I}$$
$$- \underbrace{k_2 M_R \left(\frac{B_E}{B_E + c_9}\right)}_{\text{Infection of } M_R} - \underbrace{k_3 M_R \left(\frac{B_E + wB_I + \beta F_\alpha}{B_E + wB_I + \beta F_\alpha + c_8}\right)\left(\frac{I_\gamma}{I_\gamma + f_1 I_4 + f_7 I_{10} + s_1}\right)}_{\text{Activation of macrophages}} - \underbrace{\mu_{M_R} M_R}_{\text{Natural death}}$$

M_I - MI - Infected macrophages

$$\frac{d}{dt}(M_I) = \underbrace{k_2 M_R \left(\frac{B_E}{B_E + c_9}\right)}_{\text{Infection of } M_R} - \underbrace{k_{17} M_I \left(\frac{B_I^2}{B_I^2 + (NM_I)^2}\right)}_{\text{Bursting of } M_I} - \underbrace{k_{14a} M_I \left(\frac{\left(\frac{T_C + w_3 T_1^4}{M_I}\right)}{\left(\frac{T_C + w_3 T_1^4}{M_I}\right) + c_4}\right)}_{\text{T-cell driven apoptosis of } M_I}$$

$$- \underbrace{k_{14b} M_I \left(\frac{F_\alpha}{F_\alpha + f_9 I_{10} + s_{4b}}\right)}_{\text{TNF driven apoptosis of } M_I} - \underbrace{k_{52} M_I \left(\frac{\left(\frac{T_C\left(\frac{T_1^4}{T_1^4 + C_{T_1^4}}\right) + w_1 T_1^4}{M_I}\right)}{\left(\frac{T_C\left(\frac{T_1^4}{T_1^4 + C_{T_1^4}}\right) + w_1 T_1^4}{M_I}\right) + c_{52}}\right)}_{\text{Cytotoxic T-cell driven } M_I \text{ apoptosis}}$$

$$- \underbrace{\psi_C \left(\sum_{j=1}^{n} R_{IC/EC \text{ for drug j}}^{FIC}\right)^{1/FIC} k_I(C_{eff}) M_I}_{\text{Drug-induced clearance}} - \underbrace{\mu_{M_I} M_I}_{\text{Natural death}}$$

M_A - MA - Activated macrophages

$$\frac{d}{dt}(M_A) = \underbrace{k_3 M_R \left(\frac{B_E + wB_I + \beta F_\alpha}{B_E + wB_I + \beta F_\alpha + c_8}\right)\left(\frac{I_\gamma}{I_\gamma + f_1 I_4 + f_7 I_{10} + s_1}\right)}_{\text{Activation of resting macrophages}} - \underbrace{k_4 M_A \left(\frac{I_{10}}{I_{10} + s_8}\right)}_{\text{Deactivation by } I_{10}} - \underbrace{\mu_{M_A} M_A}_{\text{Natural death}}$$

SB1.2 Mtb and caseum equations

B_I - BI - Intracellular bacterium count

$$\frac{d}{dt}(B_I) = \underbrace{\alpha_{19} \frac{B_I}{M_I} M_I \left(1 - \frac{(B_I/M_I)}{N}\right)}_{\text{Replication}} + \underbrace{k_2 \frac{N}{2} M_R \left(\frac{B_E}{B_E + c_9}\right)}_{\text{Internalization of } B_E} - \underbrace{k_{17} N M_I \left(\frac{B_I^2}{B_I^2 - (NM_I)^2}\right)}_{\text{Bursting of } M_I}$$

$$- \underbrace{k_{14a} \frac{B_I}{M_I} M_I \left(\frac{\left(\frac{T_C + w_3 T_1^4}{M_I}\right)}{\left(\frac{T_C + w_3 T_1^4}{M_I}\right) + c_4}\right)}_{\text{T-cell driven } M_I \text{ apoptosis}} - \underbrace{k_{14b} \frac{B_I}{M_I} M_I \left(\frac{F_\alpha}{F_\alpha + f_9 I_{10} + s_{4b}}\right)}_{\text{TNF driven apoptosis}}$$

$$- \underbrace{k_{52} \frac{B_I}{M_I} M_I \left(\frac{\left(\frac{T_C\left(\frac{T_1^4}{T_1^4 + C_{T_1^4}}\right) + w_1 T_1^4}{M_I}\right)}{\left(\frac{T_C\left(\frac{T_1^4}{T_1^4 + C_{T_1^4}}\right) + w_1 T_1^4}{M_I}\right) + c_{52}}\right)}_{\text{Cytotoxic T-cell driven} M_I \text{ apoptosis}} - \underbrace{\mu_{B_I} B_I}_{\text{Natural death}} - \underbrace{\mu_{M_I} \frac{B_I}{M_I} M_I}_{\text{Natural death of } M_I} - \underbrace{k_I(C_{eff}) B_I}_{\substack{\text{Drug killing of } B_I \text{ based} \\ \text{on effective killing rate}}}$$

B_E - BE - Extacellular bacterium count

$$\frac{d}{dt}(B_E) = \underbrace{\alpha_{20} B_E \left(1 - \frac{B_E}{10^6}\right)}_{\text{Replication}} + \underbrace{(1 - C_N)}_{\substack{\text{Fraction outside}\\ \text{of caseum}}} \left[\underbrace{k_{17} N M_I \left(\frac{B_I^2}{B_I^2 + (N M_I)^2} \right)}_{\text{Macrophage bursting}} + \underbrace{k_{14a} N_{fracc} \frac{B_I}{M_I} M_I \left(\frac{\left(\frac{T_C + w_3 T_1^4}{M_I}\right)}{\left(\frac{T_C + w_3 T_1^4}{M_I}\right) + c_4} \right)}_{\text{T-cell driven apoptosis}} \right.$$

$$\left. + \underbrace{k_{14b} N_{fraca} \frac{B_I}{M_I} M_I \left(\frac{F_\alpha}{F_\alpha + f_9 I_{10} + s_{4b}} \right)}_{\text{TNF driven apoptosis}} + \underbrace{\mu_{M_I} N_{fracd} \frac{B_I}{M_I} M_I}_{\text{Natural death of } M_I} \right] + \underbrace{k_{Rev} N_{Ca} \left(M_A + M_I \right) Ca \cdot B_N}_{\text{Revealing of } B_N}$$

$$- \underbrace{k_2 \frac{N}{2} M_R \left(\frac{B_E}{B_E + c_9} \right)}_{\text{Internalization of } B_E} - \underbrace{k_{15} M_A B_E}_{M_A \text{ killing of } B_E} - \underbrace{k_{18} M_R B_E}_{M_R \text{ killing of } B_E} - \underbrace{\mu_{B_E} B_E}_{\text{Natural death}} - \underbrace{k_E(C_{eff}) B_E}_{\substack{\text{Drug killing of } B_E \text{ based}\\ \text{on effective killing rate}}}$$

B_N - BN - Nonreplicating bacterium count

$$\frac{d}{dt}\left(B_N\right) = \underbrace{C_N}_{\substack{\text{Fraction inside}\\ \text{of caseum}}} \left\{ \underbrace{k_{17} M_I \left(\frac{B_I^2}{B_I^2 + (N M_I)^2} \right)}_{\text{Macrophage bursting}} + \underbrace{k_{14a} N_{fracc} \frac{B_I}{M_I} M_I \left(\frac{\left(\frac{T_C + w_3 T_1^4}{M_I}\right)}{\left(\frac{T_C + w_3 T_1^4}{M_I}\right) + c_4} \right)}_{\text{T-cell driven apoptosis}} \right.$$

$$\left. + \underbrace{k_{14b} N_{fraca} \frac{B_I}{M_I} M_I \left(\frac{F_\alpha}{F_\alpha + f_9 I_{10} + s_{4b}} \right)}_{\text{TNF driven apoptosis}} + \underbrace{\mu_{M_I} N_{fracd} \frac{B_I}{M_I} M_I}_{\text{Natural death of } M_I} \right\}$$

$$- \underbrace{\mu_{B_N} B_N}_{\text{Natural death}} - \underbrace{B_N k_{Rev} N_{Ca} Ca \cdot M_A}_{\text{Revealing of } B_N} - \underbrace{k_N(C_{eff}) B_N}_{\substack{\text{Drug killing of } B_N \text{ based}\\ \text{on effective killing rate}}}$$

Ca - CA - Caseum

$$N_f \frac{d}{dt}\left(Ca\right) = \underbrace{k_{17} M_I \left(\frac{B_I^2}{B_I^2 + (N M_I)^2} \right)}_{\text{Bursting of } M_I} + \underbrace{k_{14a} M_I \left(\frac{\left(\frac{T_C + w_3 T_1}{M_I}\right)}{\left(\frac{T_C + w_3 T_1}{M_I}\right) + c_4} \right)}_{\text{T-cell mediated apoptosis of } M_I} + \underbrace{k_{14b} M_I \left(\frac{F_\alpha}{F_\alpha + f_9 I_{10} + s_{4b}} \right)}_{\text{TNF-mediated apoptosis of } M_I}$$

$$+ \underbrace{k_{52} \frac{B_I}{M_I} M_I \left(\frac{\left(\frac{T_C \left(\frac{T_1^4}{T_1^4 + C_{T_1}^4} \right) + w_1 T_1^4}{M_I} \right)}{\left(\frac{T_C \left(\frac{T_1^4}{T_1^4 + C_{T_1}^4} \right) + w_1 T_1^4}{M_I} \right) + c_{52}} \right)}_{\text{Cytotoxic T-cell mediated apoptosis of } M_I} + \underbrace{\psi_C \left(1 - \psi_S\right) \left(\sum_{j=1}^{n} R_{IC/EC \text{ for drug j}}^{FIC} \right)^{1/FIC} k_I(C_{eff}) M_I}_{M_I \text{ killed during drug clearance}}$$

$$+ \underbrace{\mu_{M_I} M_I + \mu_{M_A} M_A + \mu_{M_R} M_R \left(\frac{F_\alpha}{F_\alpha + f_9 I_{10} + s_{4b}} \right)}_{\text{Natural death of all macrophage populations}} - \underbrace{N_{Ca} Ca M_A}_{\text{Neutrophil-driven caseum clearance}}$$

Ag - AG - Mtb antigen

$$\frac{d}{dt}(Ag) = \underbrace{k_{15}M_AB_E}_{M_A \text{ killing of } B_E} + \underbrace{k_{18}M_RB_E}_{M_R \text{ killing of } B_E} + \underbrace{\mu_{B_E}B_E}_{\text{Natural death of } B_E}$$
$$+ \underbrace{k_I(C_{eff})B_I}_{\substack{\text{Drug killing of } B_I \text{ based}\\ \text{on effective killing rate}}} + \underbrace{k_E(C_{eff})B_E}_{\substack{\text{Drug killing of } B_E \text{ based}\\ \text{on effective killing rate}}} + \underbrace{k_N(C_{eff})B_N}_{\substack{\text{Drug killing of } B_N \text{ based}\\ \text{on effective killing rate}}}$$
$$+ \underbrace{k_{14a}\frac{B_I}{M_I}M_I\left(\frac{\left(\frac{T_C+w_3T_1^4}{M_I}\right)}{\left(\frac{T_C+w_3T_1^4}{M_I}\right)+c_4}\right)}_{\text{T-cell driven } M_I \text{ apoptosis}} + \underbrace{k_{14b}\frac{B_I}{M_I}M_I\left(\frac{F_\alpha}{F_\alpha+f_9I_{10}+s_{4b}}\right)}_{\text{TNF driven aptosis}}$$
$$+ \underbrace{k_{52}\frac{B_I}{M_I}M_I\left(\frac{\left(\frac{T_C\left(\frac{T_1^4}{T_1^4+C_{T_1^4}}\right)+w_1T_1^4}{M_I}\right)}{\left(\frac{T_C\left(\frac{T_1^4}{T_1^4+C_{T_1^4}}\right)+w_1T_1^4}{M_I}\right)+c_{52}}\right)}_{B_I \text{ death by cytotoxic T-cell driven } M_I \text{ apoptosis}} + \underbrace{\mu_{B_I}B_I}_{\text{Natural Death of } B_I}$$
$$+ \underbrace{\mu_{M_I}\frac{B_I}{M_I}M_I}_{B_I \text{ death by natural death of } M_I} - \underbrace{\mu_{Ag}Ag}_{\text{Antigen clearance}}$$

SB1.3 CD4+ T-cells

T_0^4 - T40 - Primed CD4+ T-cell count

$$\frac{d}{dt}\left(T_0^4\right) = Pulled\left[T_0^4\right]_g + \underbrace{\alpha_2T_0^4\left(\frac{M_A}{M_A+c_{15}}\right)}_{\text{Proliferation}} - \underbrace{k_6I_{12}T_0^4\left(\frac{I_\gamma}{I_\gamma+f_1I_4+f_7I_{10}+s_1}\right)}_{\text{Differentiation to } T_1^4}$$
$$- \underbrace{k_7T_0^4\left(\frac{I_4}{I_4+f_2I_\gamma+s_2}\right)}_{\text{Differentiation to } T_2^4} - \underbrace{\mu_{T_0}T_0}_{\text{Natural death}}$$

T_1^4 - Th1 - Effector CD4+ T-cell count

$$\frac{d}{dt}\left(T_1^4\right) = \underbrace{k_6I_{12}T_0^4\left(\frac{I_\gamma}{I_\gamma+f_1I_4+f_7I_{10}+s_1}\right)}_{\text{Differentiation from } T_0^4} + \underbrace{k_{31}T_{EM}^4M_I\left(\frac{M_I}{M_I+3}\right)}_{\text{Differentiation from } T_{EM}^4}$$
$$- \underbrace{\mu_{T_\gamma}\left(\frac{I_\gamma}{I_\gamma+c}\right)T_1^4M_A}_{\text{IFN}\gamma \text{ apoptosis of } T_0^1} - \underbrace{\mu_{T_1^4}T_1^4}_{\text{Natural death}}$$

T_2^4 - Th2 - Effector Th2 CD4+ T-cell count

$$\frac{d}{dt}\left(T_2^4\right) = \underbrace{k_7T_0^4\left(\frac{I_4}{I_4+f_2I_\gamma+s_2}\right)}_{\text{Differentiation from } T_0^4} + \underbrace{k_{32}T_{EM}^4M_A}_{\text{Differentiation from } T_{EM}^4} - \underbrace{\mu_{T_2^4}T_2^4}_{\text{Natural death}}$$

T_{EM}^4 - EMCD4 - Effector memory CD4+ T-cell count

$$\frac{d}{dt}\left(T^4_{EM}\right) = Pulled\left[T^4_{EM}\right]_g - \underbrace{k_{31}T^4_{EM}M_I\left(\frac{M_I}{M_I+3}\right)}_{\text{Differentiation to } T^4_1} - \underbrace{k_{32}T^4_{EM}M_A}_{\text{Differentiation to } T^4_2} - \underbrace{\mu_{T^4_{EM}}T^4_{EM}}_{\text{Natural death}}$$

T^4_{Non} - CD4Non - Non-cognate CD4+ T-cell count

$$\frac{d}{dt}\left(T^4_{Non}\right) = Pulled\left[T^4_{Non}\right]_g - \underbrace{\mu_{T^4_{Non}}T^4_{Non}}_{\text{Natural death}}$$

SB1.4 CD8+ T-cells

T^8_0 - T80 - Primed CD8+ T-cell count:

$$\begin{aligned}\frac{d}{dt}\left(T^8_0\right) =& Pulled\left[T^8_0\right]_g + \underbrace{\alpha_2 T^8_0\left(\frac{M_A}{M_A+c_{15}}\right)}_{\text{Proliforation}} \\ &- \underbrace{k_6 I_{12} T^8_0\left(\frac{I_\gamma}{I_\gamma+f_1I_4+f_7I_{10}+s_1}\right)}_{\text{Differentiation to } T^8 \text{ and } T_C} - \underbrace{\mu_{T_0}T^8_0}_{\text{Natural death}}\end{aligned}$$

T^8 - T8 - Effector CD8+ T-cell count

$$\frac{d}{dt}\left(T^8\right) = \underbrace{(m)k_6 I_{12} T^8_0\left(\frac{I_\gamma}{I_\gamma+f_1I_4+f_7I_{10}+s_1}\right)}_{\text{Differentiation from } T^8_0} + \underbrace{k_{34}T^8_{EM}M_I\left(\frac{M_I}{M_I+3}\right)}_{\text{Differentiation from } T^8_{EM}} - \underbrace{\mu_{T_C\gamma}\left(\frac{I_\gamma}{I_\gamma+c_c}\right)T^8M_A}_{\text{IFN}\gamma\text{ apoptosis}} - \underbrace{\mu_{T_C}T_8}_{\text{Natural death}}$$

T_C - TC - Cytotoxic CD8+ T-cell count

$$\frac{d}{dt}\left(T_C\right) = \underbrace{(1-m)k_6 I_{12} T^8_0\left(\frac{I_\gamma}{I_\gamma+f_1I_4+f_7I_{10}+s_1}\right)}_{\text{Differentiation from } T^8_0} + \underbrace{k_{33}T^8_{EM}M_I\left(\frac{M_I}{M_I+3}\right)}_{\text{Differentiation from } T^8_{EM}} - \underbrace{\mu_{T_C\gamma}\left(\frac{I_\gamma}{I_\gamma+c_c}\right)T_CM_A}_{\text{IFN}\gamma\text{ apoptosis}} + \underbrace{\mu_{T_C}T_C}_{\text{Natural death}}$$

T^8_{EM}- EMCD8 - Effector Memory CD8+ T-cell count

$$\frac{d}{dt}\left(T^8_{EM}\right) = Pulled\left[T^8_{EM}\right]_g - \underbrace{k_{33}T^8_{EM}M_I\left(\frac{M_I}{M_I+3}\right)}_{\text{Differentiation to } T_C} - \underbrace{k_{34}T^8_{EM}M_I\left(\frac{M_I}{M_I+3}\right)}_{\text{Differentiation to } T^8} - \underbrace{\mu_{T^8_{EM}}T^8_{EM}}_{\text{Natural death}}$$

T^8_{Non} -CD8Non - Non-cognate CD8+ T-cell count

$$\frac{d}{dt}\left(T^8_{Non}\right) = Pulled\left[T^8_{Non}\right]_g - \underbrace{\mu_{T^8_{Non}}T^8_{Non}}_{\text{Natural death}}$$

SB1.5 Cytokines

F_α - TNF - concentration of Tumor Necrosis Factor α

$$\frac{d}{dt}\left(F_\alpha\right) = \underbrace{\alpha_{30} M_I}_{\text{Production by } M_I} + \underbrace{\alpha_{31} M_A \left(\frac{I_\gamma + \beta_2\left(B_E + wB_I\right)}{I_\gamma + \beta_2\left(B_E + wB_I\right) + f_1 I_4 + f_7 I_{10} + s_{10}}\right)}_{\text{IFN-IL mediated production of TNF}\alpha \text{ by } M_A}$$

$$+ \underbrace{\alpha_{32} T_1^4}_{\text{Production by } T_1^4} + \underbrace{\alpha_{33}\left(\frac{T_C + T^8}{2m}\right)}_{\text{Production by CD8}^+ \text{ T-cells}} - \underbrace{\mu_{F_\alpha} F_\alpha}_{\text{Clearance}}$$

I_γ - IG - concentration of Interferon-γ

$$\frac{d}{dt}\left(I_\gamma\right) = \underbrace{s_g\left(\frac{B_E + wB_I}{B_E + wB_I + c_{10}}\right)\left(\frac{I_{12}}{I_{12} + s_7}\right)}_{\text{Other}} + \underbrace{\alpha_{5a} T_1^4\left(\frac{M_A}{M_A + c_{5a}}\right)}_{\text{Production by } T_1^4} + \underbrace{\alpha_{5b} T^8\left(\frac{M_A}{M_A + c_{5b}}\right)}_{\text{Production by } T^8} + \underbrace{\alpha_{5c} M_I}_{\text{Production by } M_I}$$

$$+ \underbrace{\alpha_7 T_0^4\left(\frac{I_{12}}{I_{12} + f_4 I_{10} + s_4}\right) + \alpha_7 T_0^8\left(\frac{I_{12}}{I_{12} + f_4 I_{10} + s_4}\right)}_{\text{Interleukin-mediated production by primed T-cells}} - \underbrace{\mu_{I_\gamma} I_\gamma}_{\text{Clearance}}$$

I_{12} - I12 - concentration of Interleukin-12

$$\frac{d}{dt}\left(I_{12}\right) = \underbrace{s_{12}\left(\frac{B_E + wB_I}{B_E + wB_I + c_{230}}\right)}_{\text{Other}} + \underbrace{\alpha_{23} M_R\left(\frac{B_E + wB_I}{B_E + wB_I + c_{23}}\right)}_{\text{Production by } M_R} + \underbrace{\alpha_8 M_A\left(\frac{s}{s + I_{10}}\right)}_{\text{Production by } M_A} - \underbrace{\mu_{I_{12}} I_{12}}_{\text{Clearance}}$$

I_{10} - I10 - concentration of Interleukin-10

$$\frac{d}{dt}\left(I_{10}\right) = \underbrace{\delta_7\left(M_I + M_A\right)\left(\frac{s_6}{I_{10} + f_6 I_\gamma + s_6}\right)}_{\text{IFN-IL mediated production by macrophages}} + \underbrace{\alpha_{16} T_1^4 + \alpha_{17} T_2^4}_{\text{Production by CD4}^+ \text{ T-cells}} + \underbrace{\alpha_{18}\left(\frac{T_C + T^8}{2m}\right)}_{\text{Production by CD8}^+ \text{ T-cells}} - \underbrace{\mu_{I_{10}} I_{10}}_{\text{Clearance}}$$

I_4 - I4 - concentration of Interleukin-4

$$\frac{d}{dt}\left(I_4\right) = \underbrace{\alpha_{11} T_0^4}_{\text{Production by } T_0^4} + \underbrace{\alpha_{12} T_2^4}_{\text{Production by } T_2^4} - \underbrace{\mu_{I_4} I_4}_{\text{Clearance}}$$

SB2: Lymph node and blood equations

The lymph node compartment will clonally expand and differentiate effector T cells to be sent to blood. Cells in blood can be recruited to granulomas based on their cytokine levels, which is represented by terms indicated by $Pulled\left[\cdot\right]_g$ (Section B3). We select each parameter in the following equations using the LHS sampling scheme using the ranges presented in Section B5.2. In Table SB2 is an index of all state variables in the compartment representing lymph nodes and blood.

Table SB2: **State variables of lymph node and blood coupled ODEs.**

Variable	Non-symbolic	Unit	Variable description
	**Lymph Node**		
APC	APC	count	Antigen-presenting cells in LN
N_4^{LN}	LnN4	count	Mtb-specific naive CD4$^+$ T-cell count in LN
P_4^{LN}	LnP4	count	Mtb-specific precursor CD4$^+$ T-cell count in LN
E_4^{LN}	LnE4	count	Mtb-specific effector CD4$^+$ T-cell count in LN
CM_4^{LN}	LnCM4	count	Mtb-specific central memory CD4$^+$ T-cell count in LN
EM_4^{LN}	LnEM4	count	Mtb-specific effector memory CD4$^+$ T-cell count in LN
$N_{Non,4}^{LN}$	LnN4Non	count	Nonspecific naive CD4$^+$ T-cell count in LN
$CM_{Non,4}^{LN}$	LnCM4Non	count	Nonspecific central memory CD4$^+$ T-cell count in LN
N_8^{LN}	LnN8	count	Mtb-specific naive CD8$^+$ T-cell count in LN
P_8^{LN}	LnP8	count	Mtb-specific precursor CD8$^+$ T-cell count in LN
E_8^{LN}	LnE8	count	Mtb-specific effector CD8$^+$ T-cell count in LN
CM_8^{LN}	LnCM8	count	Mtb-specific central memory CD8$^+$ T-cell count in LN
EM_8^{LN}	LnEM8	count	Mtb-specific effector memory CD8$^+$ T-cell count in LN
$N_{Non,8}^{LN}$	LnN8Non	count	Nonspecific naive CD8$^+$ T-cell count in LN
$CM_{Non,8}^{LN}$	LnCM8Non	count	Nonspecific central memory CD8$^+$ T-cell count in LN
	**Blood**		
N_4^{B}	BlN4	pg/mL	Mtb-specific naive CD4$^+$ T-cell count in blood
E_4^{B}	BlE4	pg/mL	Mtb-specific effector CD4$^+$ T-cell count in blood
CM_4^{B}	BlCM4	pg/mL	Mtb-specific central memory CD4$^+$ T-cell count in blood
EM_4^{B}	BlEM4	pg/mL	Mtb-specific effector memory CD4$^+$ T-cell count in blood
$N_{Non,4}^{B}$	BlN4Non	pg/mL	Nonspecific naive CD4$^+$ T-cell count in blood
$E_{Non,4}^{B}$	BlE4Non	pg/mL	Nonspecific effector CD4$^+$ T-cell count in blood
$CM_{Non,4}^{B}$	BlCM4Non	pg/mL	Nonspecific central memory CD4$^+$ T-cell count in blood
$EM_{Non,4}^{B}$	BlEM4Non	pg/mL	Nonspecific effector memory CD4$^+$ T-cell count in blood
N_8^{B}	BlN8	pg/mL	Mtb-specific naive CD8$^+$ T-cell count in blood
E_8^{B}	BlE8	pg/mL	Mtb-specific effector CD8$^+$ T-cell count in blood
CM_8^{B}	BlCM8	pg/mL	Mtb-specific central memory CD8$^+$ T-cell count in blood
EM_8^{B}	BlEM8	pg/mL	Mtb-specific effector memory CD8$^+$ T-cell count in blood
$N_{Non,8}^{B}$	BlN8Non	pg/mL	Nonspecific naive CD8$^+$ T-cell count in blood
$E_{Non,8}^{B}$	BlE8Non	pg/mL	Nonspecific effector CD8$^+$ T-cell count in blood
$CM_{Non,8}^{B}$	BlCM8Non	pg/mL	Nonspecific central memory CD8$^+$ T-cell count in blood
$EM_{Non,8}^{B}$	BlEM8Non	pg/mL	Nonspecific effector memory CD8$^+$ T-cell count in blood

SB2.1 Antigen Presenting Cells

APC - APC - Antigen presenting cells. Received APCs are sent from granulomas, which is detailed in Section B3.

$$\frac{d}{dt}(APC) = -\mu_5 APC + \sum_{i \in \{\text{Granulomas}\}} \text{Received from Granuloma } i$$

SB2.2 CD4+ T-cells in lymph nodes

N_4^{LN} - LnN4 - CD4+ Mtb-specific naive T-cell count

$$\frac{d}{dt}\left(N_4^{LN}\right) = \alpha \left[\underbrace{k_1 N_4^B \left(\frac{APC}{APC + hs_1}\right)}_{\text{Cytokine-driveen recruitment}} + \underbrace{\xi_1 N_4^B}_{\text{LN Influx}} \right] - \underbrace{\xi_2 N_4^{LN}}_{\text{LN Efflux}} - \underbrace{k_2 N_4^{LN} APC}_{\text{Differentiation into } P_4^{LN}}$$

P_4^{LN} - LnP4 - CD4+ Mtb-specific precursor T-cell count

$$\frac{d}{dt}\left(P_4^{LN}\right) = \underbrace{k_2 N_4^{LN} APC}_{\text{Differentiation from } N_4^{LN}} + \underbrace{k_3 CM_4^{LN} APC}_{\text{Differentiation from } CM_4^{LN}} + \underbrace{k_4 P_4^{LN}\left(1 - \frac{P_4^{LN}}{\rho_1}\right)\left(\frac{APC}{APC + hs_4}\right)}_{\text{Proliferation}}$$
$$- \underbrace{k_5 P_4^{LN}\left(\frac{APC}{APC + hs_5}\right)}_{\text{Differention into } E_4^{LN}} - \underbrace{k_6 P_4^{LN}\left(1 - \frac{APC}{APC + hs_5}\right)}_{\text{Differentiation into } CM_4^{LN}} - \underbrace{\mu_6 P_4^{LN}}_{\text{Natural death}}$$

E_4^{LN}- LnE4 - CD4+ Mtb-specific effector T-cell count

$$\frac{d}{dt}\left(E_4^{LN}\right) = \underbrace{k_5 P_4^{LN}\left(\frac{APC}{APC + hs_5}\right)}_{\text{Differention from } P_4^{LN}} - \underbrace{\xi_3 E_4^{LN}}_{\text{LN Efflux}} - \underbrace{k_7 E_4^{LN}}_{\text{Differentiation into } EM_4^{LN}}$$

CM_4^{LN}- LnCM4 - CD4+ Mtb-specific central memory T-cell count

$$\frac{d}{dt}\left(CM_4^{LN}\right) = \alpha \left[\underbrace{k_8 CM_4^B \left(\frac{APC}{APC + hs_8}\right)}_{\text{Cytokine-mediated recruitment}} + \underbrace{\xi_4 CM_4^B}_{\text{LN Influx}} \right] + \underbrace{k_6 P_4^{LN}\left(1 - \frac{APC}{APC + hs_5}\right)}_{\text{Differentiation from } P_4^{LN}}$$
$$- \underbrace{k_3 CM_4^{LN} APC}_{\text{Differentiation to } P_4^{LN}} - \underbrace{\xi_5 CM_4^{LN}}_{\text{LN Efflux}}$$

EM_4^{LN} - LnEM4 - CD4+ Mtb-specific effector memory T-cell count

$$\frac{d}{dt}\left(EM_4^{LN}\right) = \underbrace{k_7 E_4^{LN}}_{\text{Differentiation from } E_4^{LN}} - \underbrace{\xi_6 EM_4^{LN}}_{\text{LN Efflux}}$$

$N_{Non,4}^{LN}$ - LnN4Non - Non-cognate naive CD4+ T-cell count

$$\frac{d}{dt}\left(N_{Non,4}^{LN}\right) = \alpha \left(\underbrace{k_1 N_{Non,4}^B \left(\frac{APC}{APC + hs_1}\right)}_{\text{Cytokine-mediated recruitment}} + \underbrace{\xi_1 N_{Non,4}^B}_{\text{LN Influx}} \right) - \underbrace{\xi_2 N_{Non,4}^{LN}}_{\text{LN Efflux}}$$

$CM_{Non,4}^{LN}$ - LnCM4Non - Non-cognate central memory CD4+ T-cell count

$$\frac{d}{dt}\left(CM_{Non,4}^{LN}\right) = \alpha\left(\underbrace{k_8 CM_{Non,4}^{B}\left(\frac{APC}{APC+hs_8}\right)}_{\text{Cytokine-mediated recruitment}} + \underbrace{\xi_4 CM_{Non,4}^{B}}_{\text{LN Influx}}\right) - \underbrace{\xi_5 CM_{Non,4}^{LN}}_{\text{LN Efflux}}$$

SB2.3 CD8+ T-cells in lymph nodes

N_8^{LN} - LnN8 - Mtb-specific naive CD8+ T-cell count

$$\frac{d}{dt}\left(N_8^{LN}\right) = \alpha\left(\underbrace{k_{10} N_8^{B}\left(\frac{APC}{APC+hs_{10}}\right)}_{\text{Cytokine-mediated recruitment}} + \underbrace{\xi_7 N_8^{B}}_{\text{LN Influx}}\right) - \underbrace{\xi_8 N_8^{LN}}_{\text{LN Efflux}} - \underbrace{k_{11} N_8^{LN} APC\left(\frac{E_4^{LN}+w_{P_4}P_4^{LN}}{E_4^{LN}+w_{P_4}P_4^{LN}+hs_{11}}\right)}_{\text{Priming mediated by CD4-related cytokines}}$$

P_8^{LN} - LnP8 - Mtb-specific precursor CD8+ T-cell count

$$\frac{d}{dt}\left(P_8^{LN}\right) = \underbrace{k_{11} N_8^{LN} APC\left(\frac{E_4^{LN}+w_{P_4}P_4^{LN}}{E_4^{LN}+w_{P_4}P_4^{LN}+hs_{11}}\right)}_{\text{Priming mediated by CD4-related cytokines}} + \underbrace{k_{12} CM_8^{LN} APC}_{\text{Differentiation from } CM_8^{LN}} + \underbrace{k_{13} P_8^{LN}\left(1-\frac{P_8^{LN}}{\rho_1}\right)\left(\frac{APC}{APC+hs_{13}}\right)}_{\text{Cytokine-mediated proliforation}} - \underbrace{k_{15} P_8^{LN}\left(1-\left\{\frac{APC}{APC+hs_{14}}\right\}\right)}_{\text{Differentiation to } CM_8^{LN}} - \underbrace{k_{14} P_8^{LN}\left\{\frac{APC}{APC+hs_{14}}\right\}}_{\text{Differentiation to } E_8^{LN}} - \underbrace{\mu_7 P_8^{LN}}_{\text{Natural death}}$$

E_8^{LN} - LnE8 - Mtb-specific effector CD8+ T-cell count

$$\frac{d}{dt}\left(E_8^{LN}\right) = \underbrace{k_{14} P_8^{LN}\left\{\frac{APC}{APC+hs_{14}}\right\}}_{\text{Differentiation from } P_8^{LN}} - \underbrace{\xi_9 E_8^{LN}}_{\text{LN Efflux}} - \underbrace{k_{16} E_8^{LN}}_{\text{Differentiation to } EM_8^{LN}}$$

CM_8^{LN} - LnCM8 - Mtb-specific central memory CD8+ T-cell count

$$\frac{d}{dt}\left(CM_8^{LN}\right) = \alpha\left[\underbrace{k_{17} CM_8^{B}\left(\frac{APC}{APC+hs_{17}}\right)}_{\text{Cytokine-mediated recruitment}} + \underbrace{\xi_{10} CM_8^{B}}_{\text{LN Influx}}\right] + \underbrace{k_{15} P_8^{LN}\left(1-\left\{\frac{APC}{APC+hs_{14}}\right\}\right)}_{\text{Differentiation from } P_8^{LN}} - \underbrace{k_{12} CM_8^{LN} APC}_{\text{Cytokine-mediated differentiation to } P_8^{LN}} - \underbrace{\xi_{11} CM_8^{LN}}_{\text{LN Efflux}}$$

EM_8^{LN} - LnEM8 - Mtb-specific effector memory CD8+ T-cell count

$$\frac{d}{dt}\left(EM_8^{LN}\right) = \underbrace{k_{16} E_8^{LN}}_{\text{Differentiation from } E_8^{LN}} - \underbrace{\xi_{12} EM_8^{LN}}_{\text{LN Efflux}}$$

$N_{Non,8}^{LN}$ - LnN8Non - Non-cognate Naive CD8+ T-cell count

$$\frac{d}{dt}\left(N_{Non,8}^{LN}\right)=\alpha\left[\underbrace{k_{10}N_{Non,8}^{B}\left(\frac{APC}{APC+hs_{10}}\right)}_{\text{Cytokine-mediated recruitment}}+\underbrace{\xi_7 N_{Non,8}^{B}}_{\text{LN Influx}}\right]-\underbrace{\xi_8 N_{Non,8}^{LN}}_{\text{LN Efflux}}$$

$CM_{Non,8}^{LN}$ - LnCM8Non - Non-cognate central memory CD8+ T-cell count

$$\frac{d}{dt}\left(C_{Non,8}^{LN}\right)=\alpha\left[\underbrace{k_{17}CM_{Non,8}^{B}\left(\frac{APC}{APC+hs_{17}}\right)}_{\text{Cytokine-mediated recruitment}}+\underbrace{\xi_{10}CM_{Non,8}^{B}}_{\text{LN Influx}}\right]-\underbrace{\xi_{11}CM_{Non,8}^{LN}}_{\text{LN Efflux}}$$

SB2.4 CD4+ T-cells in blood

N_4^B - BlN4 - Mtb-specific naive CD4+ T-cell concentration

$$\frac{d}{dt}\left(N_4^B\right)=\underbrace{\lambda S_{N_4}}_{\text{Production in thymus}}+\underbrace{\alpha^{-1}\xi_2 N_4^{LN}}_{\text{LN Efflux}}-\underbrace{k_1 N_4^B\left(\frac{APC}{APC+hs_1}\right)}_{\text{Cytokine-mediated recruitment}}-\underbrace{\xi_1 N_4^B}_{\text{LN Influx}}-\underbrace{\mu_8 N_4^B}_{\text{Natural dath}}$$

E_4^B - BlE4 - Mtb-specific effector CD4+ T-cell concentration

$$\frac{d}{dt}\left(E_4^B\right)=\underbrace{\alpha^{-1}\xi_3 E_4^{LN}}_{\text{LN Efflux}}-\underbrace{\mu_1 E_4^B}_{\text{Natural death}}-\sum_{g\in Granulomas}Pulled\left[E_4^B\right]_g$$

CM_4^B - BlCM4 - Mtb-specific central memory CD4+ T-cell concentration

$$\frac{d}{dt}\left(CM_4^B\right)=\underbrace{\alpha^{-1}\xi_5 CM_4^{LN}}_{\text{LN Efflux}}-\underbrace{\xi_4 CM_4^B}_{\text{LN Influx}}-\underbrace{k_8 CM_4^B\left(\frac{APC}{APC+hs_8}\right)}_{\text{Cytokine-mediated recruitment}}$$

EM_4^B - BlEM4 - Mtb-specific effector memory CD4+ T-cell concentration

$$\frac{d}{dt}\left(EM_4^B\right)=\underbrace{\alpha^{-1}\xi_6 EM_4^{LN}}_{\text{LN Efflux}}-\underbrace{\mu_2 EM_4^B}_{\text{Natural death}}-\sum_{g\in Granulomas}Pulled\left[EM_4^B\right]_g$$

$N_{Non,4}^B$ - BlN4Non - Non-cognate naive CD4+ T-cell concentration

$$\frac{d}{dt}\left(N_{Non,4}^B\right)=\underbrace{(1-\lambda)\,s_{N_4}}_{\text{Production in thymus}}+\underbrace{\alpha^{-1}\xi_2 N_{Non,4}^{LN}}_{\text{LN Efflux}}-\underbrace{k_1 N_{Non,4}^B\left(\frac{APC}{APC+hs_1}\right)}_{\text{Cytokine-mediated recruitment}}-\underbrace{\xi_1 N_{Non,4}^B}_{\text{LN Influx}}-\underbrace{\mu_8 N_{Non,4}^B}_{\text{Natural death}}$$

$E_{Non,4}^B$ - BlE4Non - Non-cognate effector CD4+ T-cell concentration

$$\frac{d}{dt}\left(E_{Non,4}^B\right)=\underbrace{s_{E_{Non,4}}}_{\text{Production in thymus}}-\underbrace{\mu_4 E_{Non,4}^B}_{\text{Natural death}}-\sum_{g\in Granulomas}Pulled\left[E_{Non,4}^B\right]_g$$

$CM_{Non,4}^B$ - BlCM4Non - Non-cognate central memory CD4+ T-cell concentration

$$\frac{d}{dt}\left(CM_{Non,4}^B\right)=\underbrace{\alpha^{-1}\xi_5 CM_{Non,4}^{LN}}_{\text{LN Efflux}}-\underbrace{\xi_4 CM_{Non,4}^B}_{\text{LN Influx}}-\underbrace{k_8 CM_{Non,4}^B\left(\frac{APC}{APC+hs_8}\right)}_{\text{Cytokine-mediated recruitment}}$$

$EM_{Non,4}^B$ - BlEM4Non - Non-cognate effector memory CD4+ T-cell concentration

$$\frac{d}{dt}\left(EM_{Non,4}^B\right)=\underbrace{s_{EM_{Non,4}}}_{\text{Source from thymus}}-\underbrace{\mu_2 EM_{Non,4}^B}_{\text{Natural death}}-\sum_{g\in Granulomas}Pulled\left[EM_{Non,4}^B\right]_g$$

SB2.5 CD8⁺ T-cells in blood

N_8^B - BlN8 Mtb-specific naive CD8⁺ T-cell concentration

$$\frac{d}{dt}\left(N_8^B\right) = \underbrace{\lambda s_{N_8}}_{\text{Source from thymus}} + \underbrace{\alpha^{-1}\xi_8 N_8^{LN}}_{\text{LN efflux}} - \underbrace{k_{10} N_8^B \left(\frac{APC}{APC + hs_{10}}\right)}_{\text{Cytokine-mediated recruitment}} - \underbrace{\xi_7 N_8^B}_{\text{LN Influx}} - \underbrace{\mu_9 N_8^B}_{\text{Natural death}}$$

E_8^B - BlE8 - Mtb-specific effector CD8⁺ T-cell concentration

$$\frac{d}{dt}\left(E_8^B\right) = \underbrace{\alpha^{-1}\xi_9 E_8^{LN}}_{\text{LN Efflux}} - \underbrace{\mu_3 E_8^B}_{\text{Natural death}} - \sum_{g \in Granulomas} Pulled\left[E_8^B\right]_g$$

CM_8^B - BlCM8 - Mtb-specific central memory CD8⁺ T-cell concentration

$$\frac{d}{dt}\left(CM_8^B\right) = \underbrace{\alpha^{-1}\xi_{11} CM_8^{LN}}_{\text{LN Efflux}} - \underbrace{\xi_{10} CM_8^B}_{\text{LN Influx}} - \underbrace{k_{17} CM_8^B \left(\frac{APC}{APC + hs_{17}}\right)}_{\text{Cytokine-mediated recruitment}}$$

EM_8^B - BlEM8 - Mtb-specific Effector memory CD8⁺ T-cell concentration

$$\frac{d}{dt}\left(EM_8^B\right) = \underbrace{\alpha^{-1}\xi_{12} EM_8^{LN}}_{\text{LN Efflux}} - \underbrace{\mu_4 EM_8^B}_{\text{Natural death}} - \sum_{g \in Granulomas} Pulled\left[EM_8^B\right]_g$$

$N_{Non,8}^B$ - BlN8Non - Non-cognate naive CD8⁺ T-cell concentration

$$\frac{d}{dt}\left(N_{Non,8}^B\right) = \underbrace{(1-\lambda)\, s_{N_8}}_{\text{Source from thymus}} + \underbrace{\alpha^{-1}\xi_8 N_{Non,8}^{LN}}_{\text{LN Efflux}} - \underbrace{k_{10} N_{Non,8}^B \left(\frac{APC}{APC + hs_{10}}\right)}_{\text{Cytokine-mediated recruitment}} - \underbrace{\xi_7 N_{Non,8}^B}_{\text{LN Influx}} - \underbrace{\mu_9 N_{Non,8}^B}_{\text{Natural death}}$$

$E_{Non,8}^B$ - BlE8Non - Non-cognate effector CD8⁺ T-cell concentration

$$\frac{d}{dt}\left(E_{Non,8}^B\right) = \underbrace{s_{E_{Non,8}}}_{\text{Source from thymus}} - \underbrace{\mu_3 E_{Non,8}^B}_{\text{Natural death}} - \sum_{g \in Granulomas} Pulled\left[E_{Non,8}^B\right]_g$$

$CM_{Non,8}^B$ - BlCM8Non - Non-cognate central memory CD8⁺ T-cell concentration

$$\frac{d}{dt}\left(CM_{Non,8}^B\right) = \underbrace{\alpha^{-1}\xi_{11} CM_{Non,8}^{LN}}_{\text{LN Efflux}} - \underbrace{\xi_{10} CM_{Non,8}^B}_{\text{LN Influx}} - \underbrace{k_{17} CM_{Non,8}^B \left(\frac{APC}{APC + hs_{17}}\right)}_{\text{Cytokine-mediated recruitment}}$$

$EM_{Non,8}^B$ - BlEM8Non - Non-cognate effector memory CD8⁺ T-cell concentration

$$\frac{d}{dt}\left(EM_{Non,8}^B\right) = \underbrace{s_{EM_{Non,8}}}_{\text{Source from thymus}} - \underbrace{\mu_4 EM_{Non,8}^B}_{\text{Natural death}} - \sum_{g \in Granulomas} Pulled\left[EM_{Non,8}^B\right]_g$$

SB3: Coupling granuloma equations to lymph node and blood equations

SB3.1 Migration of APCs from granulomas to lymph nodes

Each granuloma begins to send a portion of its infected macrophages to the lymph node compartment. We use the sum of infected macrophages M_I and antigen Ag as a proxy for the number of dendritic cells that take Mtb to lung draining lymph nodes. Each granuloma sends APCs to the lymph nodes after time τ_s under the assumption that granulomas do not immediately begin to send dendritic cells to the lymphatic system. We compute APCs sent by granuloma g to the lymph node as:

$$APC \text{ Sent by Granuloma } g \text{ at time } t \text{ post-founding} = \begin{cases} 0 & \text{if } t < \tau_s \\ A_{frac} \cdot (Ag + M_I) & \text{otherwise.} \end{cases}$$

SB3.2 Granulomas request T-cells, pulling them from the blood compartment

In *HostSim*, a given host begins with 13 granulomas. As infection progresses, each granuloma will begin to attempt to recruit six types of T-cells from the blood compartment: $T_0^4, T_0^8, T_{EM}^4, T_{EM}^8, T_{Non}^4$, and T_{Non}^8. At each time step, each granuloma will independently request a number of T-cells given below:

- Requests for T_0^4 :

$$Requested\left[T_0^4\right] = \underbrace{\alpha_{1a}\left(w_2 M_I + M_A\right)}_{\text{Macrophage-mediated request}} + \underbrace{Sr_{1b}\left(\frac{F_\alpha}{F_\alpha + f_8 I_{10} + s_{4b2}}\right)}_{\text{TNF mediated recruitment}}$$

- Requests for T_0^8 :

$$Requested\left[T_0^8\right] = \underbrace{\alpha_{1a}\left(M_A + w_2 M_I\right)}_{\text{Macrophage-mediated recruitment}} + \underbrace{Sr_{1b}\left(\frac{F_\alpha}{F_\alpha + f_8 I_{10} + s_{4b2}}\right)}_{\text{TNF mediated recruitment}}$$

- Requests for T_{EM}^4:

$$Requested\left[T_{EM}^4\right] = \underbrace{Sr_{4EM}\left(\frac{F_\alpha}{F_\alpha + hs_{4EM}}\right)}_{\text{TNF driven recruitment}}$$

- Requests for T_{EM}^8:

$$Requested\left[T_{EM}^8\right] = \underbrace{Sr_{8EM}\left(\frac{F_\alpha}{F_\alpha + hs_{8EM}}\right)}_{\text{TNF-mediated recruitment}}$$

- Requests for T_{Non}^4:

$$Requested\left[T_{Non}^4\right] = \underbrace{Sr_{4Non}\left(\frac{F_\alpha}{F_\alpha + hs_{4Non}}\right)}_{\text{TNF driven recruitment}}$$

- Requests for T_{Non}^8:

$$Requested\left[T_{Non}^8\right] = \underbrace{Sr_{8Non}\left(\frac{F_\alpha}{F_\alpha + hs_{8Non}}\right)}_{\text{TNF driven recruitment}}$$

The blood's response to these requests is to provide lung each granuloma with T-cells. Since we model the T-cell subpopulations in more granularity in the lymph node and blood compartments, we associate the following populations between the terminology of the lymph node and blood equations; and the granuloma equations:

(1) Granuloma requests for T_0^4 pull from Lymph/blood concentration E_4^B.
(2) Granuloma requests for T_0^8 pull from Lymph/blood concentration E_8^B.
(3) Granuloma requests for T_{EM}^4 pull from Lymph/blood concentration EM_4^B.
(4) Granuloma requests for T_{EM}^8 pull from Lymph/blood concentration EM_8^B.
(5) Granuloma requests for T_{Non}^4 pull from both Lymph/blood concentrations $E_{Non,4}^B$ and $EM_{Non,4}^B$.
(6) Granuloma requests for T_{Non}^8 pull from both Lymph/blood concentrations $E_{Non,8}^B$ and $EM_{Non,8}^B$.

For T_0^4, T_0^8, T_{EM}^4, and T_{EM}^8, the amount that is pulled from the blood to provide to granuloma g is given by:

$$\begin{aligned} \underbrace{Pulled\left[T^*\right]_g}_{T\text{ - Cell count}} &= \alpha \cdot \underbrace{Pulled\left[E^*\right]_g}_{E\text{ - Concentration}} \\ &= \begin{cases} Requested\left[T^*\right]_g & \text{if } \sum_{\text{Granulomas } j} Requested\left[T^*\right]_j < \alpha \cdot E^* \\ Relative\ Request\left[T^*\right]_g & \text{otherwise.} \end{cases} \end{aligned}$$

Here, if there are not enough T-cells in the blood to meet the amount requested by the lung granulomas, available T-cells will be divided among the requesting granulomas by using the following relative request. For granuloma g, its relative request is

$$Relative\ Request\,[T^*]_g = \frac{\alpha \cdot E^*}{\sum_{\text{All granulomas}j} Request\,[T^*]_j}.$$

For T^4_{Non} and T^8_{Non}, we combine their two associated blood concentrations. For example,

$$\begin{aligned} Pulled\left[T^4_{Non}\right]_g &= \alpha\left(Pulled\left[E^B_{Non,4}\right]_g + Pulled\left[EM^B_{Non,4}\right]_g\right) \\ &= \begin{cases} Requested\left[T^4_{Non}\right]_g & \text{if } \sum_{\text{Granulomas } j} Requested\left[T^4_{Non}\right]_j < \alpha\left(E^B_{Non,4} + EM^B_{Non,4}\right) \\ Relative\ Request\left[T^4_{Non}\right]_g & \text{otherwise} \end{cases}. \end{aligned}$$

If the blood has excess T-cells ($E^B_{Non,4}$ and $EM^B_{Non,4}$ in this case), the weight of their contribution to the granulomas is based on their current relative population:

$$\alpha \cdot Pulled\left[E^B_{Non,4}\right]_g = Requested\left[T^4_{Non}\right]_g \frac{E^B_{Non,4}}{\left(E^B_{Non,4} + EM^B_{Non,4}\right)};$$

$$\alpha \cdot Pulled\left[EM^B_{Non,4}\right]_g = Requested\left[T^4_{Non}\right]_g \frac{EM^B_{Non,4}}{\left(E^B_{Non,4} + EM^B_{Non,4}\right)}.$$

If there are not enough T-cells for the request, the relative requests are calculated similarly and the blood concentration is depleted:

$$\alpha \cdot Pulled\left[E^B_{Non,4}\right]_g = \frac{Requested\left[T^4_{Non}\right]_g}{\sum_{\text{Granulomas } j} Requested\left[T^4_{Non}\right]_j} \frac{E^B_{Non,4}}{\left(E^B_{Non,4} + EM^B_{Non,4}\right)};$$

$$\alpha \cdot Pulled\left[EM^B_{Non,4}\right]_g = \frac{Requested\left[T^4_{Non}\right]_g}{\sum_{\text{Granulomas } j} Requested\left[T^4_{Non}\right]_j} \frac{EM^B_{Non,4}}{\left(E^B_{Non,4} + EM^B_{Non,4}\right)}.$$

Pulled concentrations for T^8_{Non} are calculated in the same way.

SB4: Dissemination

In *HostSim*, nonsterile granulomas have a chance to disseminate. Each granuloma begins with a local dissemination probability adjustment L_t and nonlocal dissemination probability adjustment N_t when the granuloma is created (either primary at $t = 0$, or at some later time). At each timestep, a dissemination event is determined randomly determining:

$$\{\text{Local dissemination occurs at time } t\} = (L_t + U[0,1]) > 1$$

and

$$\{\text{Nonlocal dissemination occurs at time } t\} = (N_t + U[0,1]) > 1$$

At each time step, the probability adjustment of local dissemination increases:

$$L_t = L_{t-1} + \lambda_{local}\left(\frac{[B_I + B_E]\,(t)}{[B_I + B_E]\,(t) + k_{local}}\right).$$

Similarly, nonlocal dissemination probability adjustment builds as

$$N_t = N_{t-1} + \lambda_{nonlocal}\left(\frac{[B_I + B_E]\,(t)}{[B_I + B_E]\,(t) + k_{nonlocal}}\right).$$

A new locally-disseminated granuloma is created with its parameters selected to be normally distributed around the parameter values of the primary granuloma that it disseminated from with $\sigma = 10\%$ of the width of the parameter's range (log-transformed, if the parameter is log-uniformly sampled). This is because of the assumption

that nearby lung tissue conditions give rise to similar dynamics. Nonlocally disseminated granlomas are given parameters uniformly sampled out of the ranges presented in Section B5.

Disseminated granulomas begin with one infected macrophage and an average number of intracellular bacteria from its parent (B_I/M_I from the primary granuloma that seeded it).

SB5: Antibiotic treatment model

Our antibiotic treatment model assumes that antibiotic treatment begins on a pre-specified day $t_i > 0$ where 0 is the day of pulmonary infection. For a virtual host undergoing virtual treatment, there is a pre-specified regimen of 1 or more antibiotics. This model is separated in to two major components: pharmacokinetics and pharmacodynamics. Broadly, we assume that there are no drug-drug interactions that affect pharmacokinetics, and the processes detailed for computing drug concentrations are repeated for each drug independently.

SB5.1 Pharmacokinetics model

All concentrations are assumed to be uniformly 0 for $t < t_i$. Concentrations and drug masses are tracked independently for each drug in the regimen. Parameters governing each drug's concentration in time are given in Section B6.

SB5.1.1 Plasma and lung pharmacokinetics

"$Dose$" may be considered to be a δ-function impuse of drug once per given dosing interval. The magnitude of the impulse "i.e. dose amount" is a parameter measured in mg/kg. Note that a primary output of this set of equations is the concentration of drug in healthy lung tissue $C_{lung} = D_L/V_L$, which is updated once every hour of simulated time. Also, the variable δ_{iT} is set to either 1 or 0 based on however many transition compartments the specific drug is using.

D_{T_1} - DT1 - Dose concentration compartment 1:

$$\frac{d(D_{T_1})}{dt} = Dose - \underbrace{\delta_{2T} k_d D_{T_1}}_{\text{To } T_2} - \underbrace{\delta_{1T} k_d D_{T_1}}_{\text{Direct to } P}$$

D_{T_2} - DT2 - Dose concentration 2:

$$\frac{d(D_{T_2})}{dt} = \underbrace{\delta_{2T} k_d D_{T_1}}_{\text{From } T_1} - \underbrace{\delta_{2T} k_d D_{T_2}}_{\text{To } P}$$

D_{PT} - DPT - Drug concentration in peripheral tissue:

$$\frac{d(D_{PT})}{dt} = \underbrace{Q\left(\frac{D_P}{V_P} - \frac{D_{PT}}{V_{PT}}\right)}_{\text{Drug uptake into peripheral tissue}}$$

D_P - DP - Drug concentration in plasma:

$$\frac{d(D_P)}{dt} = \underbrace{\delta_{1T} k_d D_{T_1}}_{\text{Direct from } T_1} + \underbrace{\delta_{2T} k_d D_{T_2}}_{\text{From } T_2} - \underbrace{\mu_{D_P} \cdot \frac{D_P}{V_P}}_{\text{Clearance in plasma}} - \underbrace{Q\left(\frac{D_P}{V_P} - \frac{D_{PT}}{V_{PT}}\right)}_{\text{Drug uptake into peripheral tissue}}$$

D_L - DL - Drug concentration in the lung:

$$\frac{d}{dt}(D_L) = \underbrace{Q\left(P_L \frac{D_P}{V_P} - \frac{D_L}{V_L}\right)}_{\text{From plasma}} - \underbrace{\mu_D D_L}_{\text{Lung clearance}}$$

TABLE SB3. **Minimum inhibitory concentrations per antibiotic.**

Antibiotic	Minimum inhibitory concentration (mg/L)
Isoniazid	0.004
Rifampacin	0.002
Pyrazinamide	3
Ethambutol	0.06
Bedaquiline	0.006
Pretomanid	0.008
Linezolid	0.05
Moxifloxacin	1

SB5.1.2 Granuloma pharmacokinetics

Note that the quantity C_{lung} is considered as a constant in these equations, and is updated hourly from the whole-lung pharmacokinetics. Also note that the parameter μ_D is inherited from the virtual host's whole-lung parameter μ_D, as we assume that the pharmacokinetics of the viable cellular area are the same as the lung tissue that the granuloma is found in.

D_a - DA - Drug in cellular area, measured in mg

$$\frac{d}{dt}(D_a) = \underbrace{Q_a S A_g \left(P_a C_{lung} - \frac{D_a}{V_a}\right)}_{\text{Lung to Cellular}} - \underbrace{Q_c S A_c \left(P_c \frac{D_a}{V_a} - \frac{D_c}{V_c}\right)}_{\text{Cellular to caseum}} - \underbrace{\mu_D D_a}_{\text{Clearance}}$$

D_C - DC - Drug in caseum, measured in mg

$$\frac{d}{dt}(D_c) = \underbrace{Q_c S A_c \left(P_c \frac{D_a}{V_a} - \frac{D_c}{V_c}\right)}_{\text{Cellular to caseum}} - \underbrace{\mu_D D_c}_{\text{Clearance}}$$

SB5.1.3 Effective caseum concentration

We assume that antibiotics in caseum are effective proportional to their concentration in the caseum. However, Instead of using $C_c = D_c/V_c$ directly, we consider a modified value

$$C_c^* = C_c \cdot \beta_{drug}^{r_c \cdot \tau / r_0}$$

where r_c is the radius of caseum and τ is a fitting parameter. This means that if $r_c \ll 1$, then $C_c^* \approx C_c$ (suggesting it's easier to treat smaller granulomas)

We choose the base β - the fold-reduction of effective concentration of the drug buried in r_0 =0.4mm of caseum. This gives us the **distribution-like** function β_{Drug}^{τ}. The lab of Veronique Dartois

https://www.ncbi.nlm.nih.gov/pmc/articles/PMC5607931/ listed fraction unbound (fu%), where 100% means the drug flows freely into caseum and 0% means the drug is entirely bound by caseum macromolecules. For each drug, we choose the range of β to be the confidence interval of the range of fu% in that work.

SB5.2 Pharmacodynamics model

SB5.2.1 Minimum inhibitory concentration and active drugs

We assume that drugs will not affect the system unless they are present in sufficient concentration. Drugs that are present in sufficient concentration, *active drugs*, are used in all calculations in the following subsections. Minimum inhibitory concentrations for each drug are given in Table SB3.

SB5.2.2 Pharmacodynamics modeling

We find KillBI/BE/BN $\delta_I, \delta_E, \delta_N$ based on the concentrations of active drugs from the PK model. Note if $V_a, V_c = 0$, the Hill fraction is considered to be 1). Units of D_x are in mg, and V_a are calculated in L. The equations to determine kill-rates in the absence of drug-drug interaction are

$$k_I = E_{B_I,max}\left(\frac{\left(\frac{D_a}{V_a}\right)^{h_{B_I}}}{\left(\frac{D_a}{V_a}\right)^{h_{B_I}} + c_{50,B_I}^{h_{B_I}}}\right);$$

$$k_E = E_{B_E,max}\left(\frac{\left(\frac{D_a}{V_a}\right)^{h_{B_E}}}{\left(\frac{D_a}{V_a}\right)^{h_{B_E}} + c_{50,B_E}^{h_{B_E}}}\right);$$

$$\text{and } \delta_N = E_{B_N,max}\left(\frac{\left(\frac{D_c}{V_c}\right)^{h_{B_N}}}{\left(\frac{D_c}{V_c}\right)^{h_{B_N}} + c_{50,B_N}^{h_{B_N}}}\right).$$

SB5.2.3 Modeling drug-drug interactions

We incorporate drug-drug interactions into our model regimens when multiple drugs are present within a physiological compartment (e.g., cellular area or caseum). Briefly, we adjust effective concentrations of active drugs using fractional inhibitory concentrations (FICs) of drug combinations predicted by an *in silico* tool, INDIGO-MTB (inferring drug interactions using chemogenomics and orthology optimized for Mtb), as we have done previously. INDIGO-MTB is a machine learning based tool that uses known drug interactions and drug transcriptomics data to predict unknown drug interactions in the form of FICs. FIC values lower or higher than 1 means the drugs are synergistic or antagonistic, respectively, whereas an FIC value of 1 means the drugs do not interact, i.e., they are additive.

We model drug interaction by converting concentrations of each active drug i (C_i) to equipotent concentrations of drug i_{max}, the drug with highest maximal killing rate (i.e., highest E_{max}). To do that, we calculate the adjusted concentration of i ($C_{i,adj}$), which is the concentration of i_{max} that would kill Mtb with the same rate as drug i with concentration C_i:

$$C_{i,adj} = \left(\frac{C_{i_{max},50}^{h_{i_{max}}} C_i^{h_i}}{\frac{E_{max,i_{max}}}{E_{max,i}}\left(C_i^{h_i} + C_{i,50}^{h_i}\right) + C_i^{h_i}}\right)^{1/h_{i_{max}}}$$

where $C_{i_{max},50}$ and $C_{i,50}$ are the concentration of i_{max} and i at which half maximal killing is achieved, respectively, $E_{max,i_{max}}$ and $E_{max,i}$ are the maximal killing rate constants of drug i_{max} and drug i, respectively, and $h_{i_{max}}$ and h_i are the Hill coefficients of drug i_{max} and drug i, respectively. Once we calculate the adjusted concentrations of all drugs in a compartment, we determine the effective concentration (C_{eff}) with the following equation:

$$C_{eff} = \left(\sum_{i=1}^{n} C_{i,adj}^{FIC}\right)^{1/FIC}$$

where $C_{i,adj}$ is the adjusted concentration of drug i, n is the number of drugs in a compartment and FIC is the FIC value predicted for these n drugs by INDIGO-MTB. Then, we calculate the effective killing rate k by using C_{eff} and Hill parameters of i_{max} ($E_{max,i_{max}}$, $C_{50,i_{max}}$, $h_{i_{max}}$):

$$k\left(C_{eff}\right) = E_{max,i_{max}} \frac{C_{eff}^{h_{i_{max}}}}{C_{eff}^{h_{i_{max}}} + C_{i_{max},50}^{h_m}}.$$

SB5.2.4 Clearance of infected macrophages by drugs

We add the following terms to macrophage and caseum dynamics. Parameters ψ_i represent granuloma-driven factors contributing to heterogeneity macrophage clearance. We assume that the heterogeneity of macrophage clearance driven by drug PK/PD variability is accounted for by other factors in the drug-induced macrophage clearance terms in Section B1. Notably, those factors depend upon $R_{IC/EC}$, the ratio of intracellular to extracellular drugs (Table SB4).

TABLE SB4. **Ratio of intracellular to extracellular drugs.**

Antibiotic	$R_{IC/EC}$
Isoniazid	1.3
Rifampacin	6.5
Pyrazinamide	0.6
Ethambutol	10.3
Bedaquiline	482
Pretomanid	6.6
Linezolid	3.6
Moxifloxacin	17.5

SB6: Parameter ranges and initial conditions

Table SB5: **Granuloma parameter ranges.** Distributions listed as 'u' are uniformly sampled within the given range, and 'l' are log-uniformly sampled from within the given range. Note that the rate constants k_i in the granuloma compartment and LN/blood compartment are distinct. Certain parameters indicated with * have values computed dynamically by other model components. Also note that for space, we refer to "rates" as shorthand for "rate constants" in all following tables.

Parameter	Non-symbolic	Units	Granuloma parameter description	Minimum Value	Maximum Value	Distribution
ψ_C	psiC	-	Antibiotic clearance rate of M_I	0.0001	0.001	u
ψ_S	psiS	-	Survival rate of cleared M_I	0	1	u
C_{eff}	Ceff	-	Effective concentration of antibiotic	*	*	-
α_{4a}	alpha4a	1/day	Macrophage-driven M_R recruitment rate	0.7246	0.9097	u
β	Beta	1/pg	Scaling factor for F_α for M_R activation	8652476.9683	11390938.3080	u
w	w	-	Contribution of B_I to M_R recruitment	0.2601	0.3632	u
w_2	w2	-	Contribution of M_I to M_R recruitment	0.1	0.5	u
w_3	w3	-	Contribution of T_1^4 to M_I apoptosis	0.9572	1.1725	u
Sr_{4b}	Sr4b	1/day	F_α recruitment rate of M_R	400.3165	961.7117	u
f_8	f8	-	Ratio adjustment of I_{10}/F_α on M_R recruitment	0.02568	0.9748	u
f_9	f9	-	Ratio adjustment of I_{10}/F_α for apoptosis rates	0.1	1.0	u
s_{4b}	s4b	pg/mL	Half saturation of F_α on M_R recruitment	43.2587	432.1618	l
k_4	k4	1/day	M_A decativation by I_{10}	0.06138	0.1816	u
s_8	s8	pg/mL	Half saturation of I_{10} on M_A deactivation	18.2688	156.2867	l
k_2	k2	1/day	M_R infection rate	0.1126	0.4900	u
c_9	c9	count	Half saturation of B_E on M_R infection	1305.8725	8590.4539	u
k_3	k3	1/day	M_R activation rate	0.1080	0.5012	u
f_1	f1	-	Ratio adjustment of I_4/I_γ on M_R activation	132.009929	477.7016	u
s_1	s1	pg/mL	Half saturation of I_γ dependent M_R activation	2.6104	120.4874	l
c_8	c8	count	Half saturation of B_E and B_I on M_R activation	345.3760	681.6706	u
μ_{M_R}	muMR	1/day	M_R death rate	0.004432	0.005695	u
k_{17}	k17	1/day	Maximum rate of M_I bursting	0.03598	0.3017	u
N	N	count	Carrying capacity of Mtb in one M_I	5	40	u
k_{14a}	k14a	1/day	T-cell induced apoptosis rate of M_I	0.2726	0.45	u
c_4	c4	count	Half saturation of T_1^4/M_I on T-cell apoptosis	905.8568	9420.9084	l

Table SB5: **Granuloma parameter ranges.** Distributions listed as 'u' are uniformly sampled within the given range, and 'l' are log-uniformly sampled from within the given range. Note that the rate constants k_i in the granuloma compartment and LN/blood compartment are distinct. Certain parameters indicated with * have values computed dynamically by other model components. Also note that for space, we refer to "rates" as shorthand for "rate constants" in all following tables.

Parameter	Non-symbolic	Units	Granuloma parameter description	Minimum Value	Maximum Value	Distribution
k_{14b}	k14b	1/day	F_α-induced apoptosis rate	0.2230	0.4355	u
k_{52}	k52	1/day	Cytotoxic killing rate of M_I	0.08592	0.3519	u
w_1	w1	-	Contribution of T_1^4 to cytotoxic killing	0.2192	0.7413	u
c_{52}	c52	count	Half-saturation of T_C on cytotoxic M_I killing	2588.9284	8088.5625	u
$C_{T_1^4}$	cT1	count	Half saturation of T_1^4 on cytotoxic killing	80.6446	300.4901	u
μ_{M_I}	muMI	1/day	M_I death rate	0.002965	0.003799	u
μ_{M_A}	muMA	1/day	M_A death rate	0.05	0.1	u
α_{1a}	alpha1a	1/day	Macrophage request rate of T_0^4	0.1769	0.4336	u
Sr_{1b}	Sr1b	1/day	F_α dependent T_0^4 recruitment	23767.7947	55329.7803	u
s_{4b2}	s4b2	pg/mL	Half saturation of F_α dependent T_0^4 recruitment	462.3252	1057.4677	l
α_2	alpha2	1/day	Maximum growth rate of T_0^4	0.2444	0.9666	u
c_{15}	c15	count	Half saturation of M_A proliferation of T_0^4	531.8574	9574.4150	u
k_6	k6	1/day	Maximum T_0^4 to T_1^4 differentiation rate	0.01372	0.09727	u
f_7	f7	-	Effect of I_{10} on I_γ induced $T_0^4 \rightarrow T_1^4$ differentiation	5.7185	45.2677	u
k_7	k7	1/day	Maximum rate of $T_0^4 \rightarrow T_2^4$ differentiation	0.2437	0.6525	u
f_2	f2	-	Ratio adjustment of I_γ/I_4 on $T_0^4 \rightarrow T_2^4$ differentiation	0.1938	0.4207	u
s_2	s2	pg/mL	Half saturation of I_4 for $T_0^4 \rightarrow T_2^4$ differentiation	100.3818	964.1090	l
μ_{T_0}	muT0	1/day	CD3$^+$ primed T-cell death rate	0.1957	0.2474	u
m	m	-	Fraction of differentiating T_0^8 that become T^8 (not T_C)	0.1064	0.9043	u
μ_{T_g}	muTg	1/day	T_C death rate	0.005308	0.01990	l
c	c	pg/mL	Half saturation of I_γ on T_1^4 apoptosis	462.9043	3161.3955	u
$\mu_{T_1^4}$	muT1	1/day	T_1^4 death rate	0.2848	0.3698	u
$\mu_{T_2^4}$	muT2	1/day	T_2^4 death rate	0.2913	0.3678	u
$\mu_{T_{C\gamma}}$	muTCg	1/day	I_γ driven apoptosis rate of T_C and T^8	0.006021	0.09506	l
c_c	cc	pg/mL	Half saturation of I_γ on T_C and T^8 apoptosis	368.2888	9634.1767	u
μ_{T_C}	muTC	1/day	T_C death rate	0.2570	0.3325	u

Table SB5: **Granuloma parameter ranges.** Distributions listed as 'u' are uniformly sampled within the given range, and 'l' are log-uniformly sampled from within the given range. Note that the rate constants k_i in the granuloma compartment and LN/blood compartment are distinct. Certain parameters indicated with * have values computed dynamically by other model components. Also note that for space, we refer to "rates" as shorthand for "rate constants" in all following tables.

Parameter	Non-symbolic	Units	Granuloma parameter description	Minimum Value	Maximum Value	Distribution
α_{30}	alpha30	1/day	Rate of F_α production by M_I	0.04521	0.09694	u
α_{31}	alpha31	1/day	Rate of F_α production by M_A	0.03113	0.09610	u
β_2	beta2	1/pg	Scaling factor of Mtb on F_α production by M_A	10944.8638	13022.3032	u
s_{10}	s10	pg/mL	Half saturation of I_γ on F_α production by M_A	102.8084	1500.4010	u
α_{32}	alpha32	pg/(mL*day)	F_α production rate by T_1^4	0.1843	0.3102	u
α_{33}	alpha33	pg/(mL*day)	F_α production rate by T^8 and T_C	0.1633	0.2997	u
μ_{TNF}	muTNF	1/day	F_α clearance rate	0.9334	1.5101	u
s_g	sg	pg/(mL*day)	I_γ production from other sources (e.g. dendritic cells)	846.3332	9484.1532	l
c_{10}	c10	count	Half saturation of I_γ production from other sources	315332.8289	536623.8464	u
s_7	s7	pg/mL	Half saturation of I_{12} on I_γ production from other sources	891.2493	1151.7672	u
α_{5a}	alpha5a	pg/day	Rate of I_γ production by T_1^4	0.5443	0.8698	u
c_{5a}	c5a	count	Half saturation of M_A on I_γ production by T_1^4	303.7365	687.08808	u
α_{5b}	alpha5b	pg/day	I_γ production by T^8	1.1445	15.1796	u
α_{5c}	alpha5c	pg/day	I_γ production by M_I	0.5592	0.9184	u
c_{5b}	c5b	count	Half saturation of M_A on I_γ production by T^8	235.8238	846.5511	u
α_7	alpha7	pg/day	I_γ production by T_0^4	0.08541	0.3098	u
f_4	f4	-	Adjustment of I_{10}/I_{12} on I_γ production	1.2966	1.6715	u
s_4	s4	pg/mL	Half saturation of I12 on I_γ	321.7448	865.2110	u
μ_{I_γ}	muIG	1/day	I_γ clearance rate	6.4475	12.6934	u
α_{23}	alpha23	pg/day	Rate of I_{12} production by M_R	0.003485	0.004652	u
c_{23}	c23	count	Half saturation of Mtb on I_{12} production by M_R	157.08445	525.4198	u
α_8	alpha8	pg/day	Rate of production of I_{12} by M_A	0.3764	0.8612	u
s_{12}	s12	pg/day	Rate of production of I_{12} by dendritic cells	2361.1941	4060.7654	u
c_{230}	c230	count	Half-saturation of Mtb in I_{12} production by DCs	365.6847	761.7284	u
$\mu_{I_{12}}$	muI12	1/day	I_{12} clearance rate	0.9319	1.2420	u
s	s	pg/mL	I_{10} effect on I_{12} production by M_A	191.7181	694.3849	u
δ_7	delta7	pg/day	I_{10} production by M_A	0.1106	0.6169	u

Table SB5: **Granuloma parameter ranges.** Distributions listed as 'u' are uniformly sampled within the given range, and 'l' are log-uniformly sampled from within the given range. Note that the rate constants k_i in the granuloma compartment and LN/blood compartment are distinct. Certain parameters indicated with * have values computed dynamically by other model components. Also note that for space, we refer to "rates" as shorthand for "rate constants" in all following tables.

Parameter	Non-symbolic	Units	Granuloma parameter description	Minimum Value	Maximum Value	Distribution
s_6	s6	pg/mL	Half saturation of I_{10} on self	587.3834	858.9373	u
f_6	f6	-	Ratio adjustment of I_γ on I_{10}	0.3011	0.3889	u
α_{16}	alpha16	pg/day	I_{10} production by T_1^4	0.4280	0.6693	u
α_{17}	alpha17	pg/day	I_{10} production by T_2^4	0.4149	0.4786	u
α_{18}	alpha18	pg/day	I_{10} production by T_C and T^8	0.5184	0.6648	u
$\mu_{I_{10}}$	muI10	1/day	I_{10} clearance rate	0.5904	4.007931	u
α_{11}	alpha11	pg/day	I_4 production by T_0^4	0.02944	0.06404	u
α_{12}	alpha12	pg/day	I_4 production by T_2^4	0.02110	0.06407	u
μ_{I_4}	muI4	1/day	I_4 clearance rate	2.3700	3.08806	u
α_{19}	alpha19	1/day	B_I growth rate	0.1081	1.6183	l
α_{20}	alpha20	1/day	B_E growth rate	0.1927	0.6209	u
N_{fracc}	Nfracc	-	Fraction of surviving B_I released by T-cell M_I apop.	0.3074	0.7893	u
N_{fraca}	Nfraca	-	Fraction of surviving B_I released by TNF M_I apop.	0.2680	0.5658	u
k_{15}	k15	1/day	B_E killing rate by M_A	0.04058	0.1173	u
k_{18}	k18	1/day	B_E killing rate by M_R	0.0003264	0.0005505	u
N_{fracd}	Nfracd	-	Fraction of surviving B_I released by M_I natural death.	0.0008721	0.001098	u
μ_{B_I}	muBI	1/day	B_I death rate	0.00002973	0.00004521	u
μ_{B_E}	muBE	1/day	B_E death rate	0.000000002053	0.000000003702	u
Sr_{4Non}	Sr4Non	1/day	Rate of TNF-driven T_{Non}^4 recruitment	158.6388	450.4727	u
hs_{4Non}	hs4Non	pg/mL	Half saturation of TNF in T_{Non}^4 recruitment	6.1706	49.6606	u
μ_{4Non}	mui4Non	1/day	Death rate of T_{Non}^4	0.2631	0.3579	u
Sr_{8Non}	Sr8Non	1/day	Rate of TNF-driven T_{Non}^8 recruitment	156.08771	450.8067	u
hs_{8Non}	hs8Non	pg/day	Half saturation of TNF in T_{Non}^8 recruitment	4.3815	40.4821	u
$\mu_{T_{Non}^8}$	mui8Non	1/day	Death rate of T_{Non}^8	0.2644	0.3543	u
Sr_{4EM}	Sr4EM	1/day	Rate of TNF-driven T_{EM}^4 recruitment	5145.1195	10500.04020	u
hs_{4EM}	hs4EM	pg/mL	Half saturation of TNF in T_{EM}^4 recruitment	100.0	200.0	u

Table SB5: **Granuloma parameter ranges.** Distributions listed as 'u' are uniformly sampled within the given range, and 'l' are log-uniformly sampled from within the given range. Note that the rate constants k_i in the granuloma compartment and LN/blood compartment are distinct. Certain parameters indicated with * have values computed dynamically by other model components. Also note that for space, we refer to "rates" as shorthand for "rate constants" in all following tables.

**Parameter**	**Non-symbolic**	**Units**	**Granuloma parameter description**	**Minimum Value**	**Maximum Value**	**Distribution**
$\mu_{T^4_{EM}}$	mui4EM	1/day	Death rate of T^4_{EM}	0.001368	0.002737	u
k_{31}	k31	1/day	Differentiation rate of $T^4_{EM} \to T^4_1$	0.05592	0.1	l
k_{32}	k32	1/day	Differentiation rate of $T^4_{EM} \to T^4_2$	0.0006898	0.001086	u
Sr_{EM^8}	Sr8EM	1/day	Rate of TNF-driven T^8_{EM} recruitment	60.2088	115.9625	u
hs_{EM^8}	hs8EM	pg/mL	Half saturation of TNF in T^8_{EM} recruitment	100	300	u
$\mu_{T^8_{EM}}$	mui8EM	1/day	Death rate of T^8_{EM}	0.001368	0.002737	u
k_{33}	k33	1/day	Differentiation rate of $T^8_{EM} \to T_C$	0.06339	0.1	l
$k34$	k34	1/day	Differentiation rate of $T^8_{EM} \to T^8$	0.05329	0.1	l
τ_s	taus	days	Time that granuloma begins sending APCs to LN	4	17	u
A_{frac}	APCpercent	-	Fraction of M_I and Ag that gets sent as APCs	0.1723	0.2893	u
k_{local}	localDissemCFUHalf	count	Half-saturation of Mtb for local dissemination	10000	50000	u
λ_{local}	localDissemLambda	1/day	Maximum rate of local dissemination	0.0005	0.009	l
$k_{nonlocal}$	nonLocalDissemCFUHalf	count	Half-saturation of Mtb for nonlocal dissemination	5223.04303	10608.01033	u
$\lambda_{nonlocal}$	nonLocalDissemLambda	1/day	Maximum rate of nonlocal dissemination	0.0001	0.002	u
μ_{B_N}	muBN	1/day	Death rate of B_N	0	0	u
C_N	CN	-	Fraction of released B_I that become B_N	0.0003125	0.08172	l
μ_{Ag}	muAg	1/day	Decay rate of Mtb antigen	0.003985	0.02686	u
N_f	Nf	Mϕ/caseum	Macrophage biomass that becomes caseum	13	18	u
N_{Ca}	Nca	1/(day*caseum)	Clearance rate of caseum by neutrophils	0.000001173	0.000008621	u

Table SB6: **Granuloma ODE initial conditions.** Notations are as in table SB5.

Initial Condition	Non-Symbolic	Units	Granuloma initial condition description	Min initial value	Max initial value	Distribution
$M_R(0)$	MR	count	Resting macrophages	1.0	5.0	u
$M_I(0)$	MI	count	Infected macrophages	1.0	1.0	u
$M_A(0)$	MA	count	Activated macrophages	1.0	1.0	u
$B_I(0)$	BI	count	Intracellular Mtb	1.0	1.0	u
$B_E(0)$	BE	count	Extracellular Mtb	0	0	u
$B_N(0)$	BN	count	Non-replicating Mtb	0	0	u
$T_0^4(0)$	T40	count	Mtb-specific primed CD4$^+$ T-cells	0	0	u
$T_1^4(0)$	Th1	count	Mtb-specific Th1 CD4$^+$ T-cells	0	0	u
$T_{Non}^4(0)$	CD4Non	count	Nonspecific CD4$^+$ T-cells	0	0	u
$T_2^4(0)$	Th2	count	Mtb-specific Th2 CD4$^+$ T-cells	0	0	u
$T_{EM}^8(0)$	EMCD4	count	Mtb-specific CD4$^+$ effector memory T-cells	0	0	u
$T_0^8(0)$	T80	count	Mtb-specific primed CD8$^+$ T-cells	0	0	u
$T_C(0)$	TC	count	Mtb-specific cytotoxic CD8$^+$ T-cells	0	0	u
$T^8(0)$	T8	count	Mtb-specific effector CD8$^+$ T-cells	0	0	u
$T_{EM}^8(0)$	EMCD8	count	Mtb-specific CD8$^+$ effector memory T-cells	0	0	u
$T_{Non}^8(0)$	CD8Non	count	Nonspecific CD8$^+$ T-cells	0	0	u
$F_\alpha(0)$	TNF	pg/mL	TNF-α concentration	0	0	u
$I_\gamma(0)$	IG	pg/mL	IFN-γ concentration	0	0	u
$I_{12}(0)$	I12	pg/mL	Interleukin 12 concentration	0	0	u
$I_{10}(0)$	I10	pg/mL	Interleukin 10 concentration	0	0	u
$I_4(0)$	I4	pg/mL	Interleukin 4 concentration	0	0	u
$Ca(0)$	CA	mass of $M\phi$	Necrotic tissue mass	0	0	u
$Ag(0)$	AG	mass Ag/Mtb	Antigen mass within granulomas	0	0	u

Table SB7: **Lymph node and blood coupled ODE parameter ranges.** Notations are as in table SB5. Note that the rate constants k_i in the granuloma compartment and LN/blood compartment are different.

Parameter	non-symbolic	Units	LN/blood parameter description	Lower range	Upper range	Distribution
α	alpha	μL	Conversion from Blood concentration to LN cell counts	360000.0	360000.0	u
Host LNs	hostLn	count	Number of involved LNs in host (Used for initial conditions)	5.0	5.0	-
λ	lambda	-	Frequency of Mtb-specific naive cells in the host	0.0001	0.0001	u
hs_1	hs1	count	Half saturation of APC in N_4^B recruitment to LN	1092.8546	8061.6792	l
hs_{10}	hs10	count	Half saturation of APC in N_8^B recruitment to LN	45.5413	87.7386	u
hs_{11}	hs11	count	Half saturation of CD4$^+$ surrogates in N_8^{LN} priming	12.9592	47.9200	u
hs_{13}	hs13	count	Half saturation of APC in P_8^{LN} proliforation	2684.2696	4055.8241	u
hs_{14}	hs14	count	HS of APC in $P_8^{LN} \to E_8^{LN}$ and $P_8^{LN} \to CM_8^{LN}$differentiation	1904.3871	4144.4838	u
hs_{17}	hs17	count	Half saturation of APC in CM_8^B recruitment to LN	66.1225	403.04107	u
hs_4	hs4	count	Half saturation of APC in P_4^{LN} proliforation	4057.5368	28401.2710	u
hs_5	hs5	count	HS of APC in $P_4^{LN} \to E_4^{LN}$ and $P_4^{LN} \to CM_4^{LN}$ differentiation	4134.3783	8006.2583	u
hs_8	hs8	count	Half saturation of APC in $CM_{Non,4}^B$ recruitment to LN	40.4710	57.04244	u
k_1	k1	1/day	Rate of N_4^B recruitment to LN	0.6880	0.8540	u
k_{10}	k10	1/day	Rate of N_8^B recruitment to LN	0.5380	0.6764	u
k_{11}	k11	1/day	Rate of N_8^{LN} priming	0.0001043	0.0002272	u
k_{12}	k12	1/day	CM_8^{LN} reactivation rate	0.0001206	0.0007482	u
k_{13}	k13	1/day	Rate of P_8^{LN} proliforation	0.1961	0.7954	u
k_{14}	k14	1/day	Rate of $P_8^{LN} \to E_8^{LN}$ differentiation	0.2548	0.7351	u
k_{15}	k15	1/day	Rate of $P_8^{LN} \to CM_8^{LN}$ differentiation	0.5286	0.8602	u
k_{16}	k16	1/day	Rate of $E_8^{LN} \to EM_8^{LN}$ differentiation	0.1573	0.8217	u
k_{17}	k17	1/day	Rate of CM_8^B recruitment to LN	0.3483	0.9012	u
k_2	k2	1/day	Rate of $N_4^{LN} \to P_4^{LN}$ differentiation	0.3279	0.8656	u
k_3	k3	1/day	Rate of $CM_4^{LN} \to P_4^{LN}$ differentiation	0.02161	0.07979	u
k_4	k4	1/day	Rate of P_4^{LN} proliforation	2.08336	4.7319	u
k_5	k5	1/day	Rate of $P_4^{LN} \to E_4^{LN}$ differentiation	0.2721	0.8993	u
k_6	k6	1/day	Rate of $P_4^{LN} \to CM_4^{LN}$ differentiation	0.3110	0.8645	u
k_7	k7	1/day	Rate of $E_4^{LN} \to EM_4^{LN}$ differentiation	0.6220	0.9249	u

Table SB7: **Lymph node and blood coupled ODE parameter ranges.** Notations are as in table SB5. Note that the rate constants k_i in the granuloma compartment and LN/blood compartment are different.

Parameter	non-symbolic	Units	LN/blood parameter description	Lower range	Upper range	Distribution
k_8	k8	1/day	Rate of CM_4^B recruitment to LN	0.02620	0.06984	u
μ_1	mu1	1/day	Death rate of E_4^B	0.2	0.2	u
μ_2	mu2	1/day	Death rate of EM_4^B	0.001368	0.002737	u
μ_3	mu3	1/day	Death rate of E_8^B	0.2	0.2	u
μ_4	mu4	1/day	Death rate of EM_8^B	0.001368	0.002737	u
μ_5	mu5	1/day	Death rate of APC	0.05	0.05	u
μ_6	mu6	1/day	Death rate of P_4^{LN}	0.0005	0.0005	u
μ_7	mu7	1/day	Death rate of P_8^{LN}	0.015	0.015	u
μ_8	mu8	1/day	Death rate of N_4^B	0.3	0.3	u
μ_9	mu9	1/day	Death rate of μ_9^{LN}	0.05	0.05	u
ρ_1	rho1	count	Precursor cell carrying capacity	3467280.5534	14650451.8418	u
w_{P_4}	Wp4	-	Contribution of P_4^{LN} in P_8^{LN} priming	0.7355	0.7355	u
ξ_{11}	xi11	1/day	$CM_8^{LN} \rightarrow CM_8^B$ LN efflux rate	0.2750	1.2233	u
ξ_{12}	xi12	1/day	$EM_8^{LN} \rightarrow EM_8^B$ LN efflux rate	0.2190	1.5214	u
ξ_2	xi2	1/day	$N_4^{LN} \rightarrow N_4^B$ LN efflux rate	1.9942	4.5066	u
ξ_3	xi3	1/day	$E_4^{LN} \rightarrow E_4^B$ LN efflux rate	16.9937	23.6017	u
ξ_5	xi5	1/day	$CM_4^{LN} \rightarrow CM_4^B$ LN Efflux rate	1.07179	3.9185	u
ξ_6	xi6	1/day	$EM_4^{LN} \rightarrow EM_4^B$ LN Efflux rate	0.1567	29.2311	u
ξ_8	xi8	1/day	$N_8^{LN} \rightarrow N_8^B$ LN Efflux rate	0.6344	2.2140	u
ξ_9	xi9	1/day	$E_8^{LN} \rightarrow E_8^B$ LN Efflux rate	1.8734	4.4935	u
ξ_1	xi1	1/day	$N_4^B \rightarrow N_4^{LN}$ LN Influx rate	0.3988	0.9013	u
ξ_4	xi4	1/day	$CM_4^B \rightarrow CM_4^{LN}$ LN Influx rate	0.2143	0.7837	u
ξ_7	xi7	1/day	$N_8^B \rightarrow N_8^{LN}$ LN Influx rate	0.1268	0.4428	u
ξ_{10}	xi10	1/day	$CM_8^B \rightarrow CM_8^{LN}$ LN Influx rate	0.05500	0.2446	u
s_{N_4}	Sn4	1/day*μL	Production rate of naive CD4$^+$ T-cells	20.1207	47.9125	u
s_{N_8}	Sn8	1/day*μL	Production rate of naive CD8$^+$ T-cells	0.3830	0.4868	u
$s_{E_{Non,4}}$	Senc4	1/day*μL	Production rate of effector non-specific CD4$^+$ T-cells	15.3195	65.7082	u

Table SB7: **Lymph node and blood coupled ODE parameter ranges.** Notations are as in table SB5. Note that the rate constants k_i in the granuloma compartment and LN/blood compartment are different.

Parameter	non-symbolic	Units	LN/blood parameter description	Lower range	Upper range	Distribution
$s_{EM_{Non,4}}$	Semnc4	1/day*μL	Production rate of effector memory non-specific $CD4^+$ T-cells	6.4041	17.04971	u
$s_{E_{Non,8}}$	Senc8	1/day*μL	Production rate of effector non-specific $CD8^+$ T-cells	10.05032	69.3024	u
$s_{EM_{Non,8}}$	Semnc8	1/day*μL	Production rate of effector memory non-specific $CD8^+$ T-cells	2.8856	5.5002	u

Table SB8: **Lymph node and blood initial conditions.** Notations are as in table SB5. *We assume that at $t = 0$, blood and lymph node concentrations are at equilibrium, so blood concentrations are calculated via their lymph-node starting count via the conversion factor α and HostLNs.

Initial condition	Non-Symbolic	Unit	LN/Blood initial condition description	Lower range	Upper range	Distribution
$APC(0)$	APC	count	Antigen-presenting cells in LN	0	0	u
$N_4^{LN}(0)$	LnN4	count	Mtb-specific naive CD4$^+$ T-cell count in LN	2897.3857	6899.4095	u
$P_4^{LN}(0)$	LnP4	count	Mtb-specific precursor CD4$^+$ T-cell count in LN	0	0	u
$E_4^{LN}(0)$	LnE4	count	Mtb-specific effector CD4$^+$ T-cell count in LN	0	0	u
$CM_4^{LN}(0)$	LnCM4	count	Mtb-specific central memory CD4$^+$ T-cell count in LN	0	0	u
$EM_4^{LN}(0)$	LnEM4	count	Mtb-specific effector memory CD4$^+$ T-cell count in LN	0	0	u
$N_4^{B}(0)$	BlN4	pg/mL	Mtb-specific naive CD4$^+$ T-cell concentration in blood	*	-	u
$E_4^{B}(0)$	BlE4	pg/mL	Mtb-specific effector CD4$^+$ T-cell concentration in blood	*	-	u
$CM_4^{B}(0)$	BlCM4	pg/mL	Mtb-specific central memory CD4$^+$ T-cell concentration in blood	*	-	u
$EM_4^{B}(0)$	BlEM4	pg/mL	Mtb-specific effector memory CD4$^+$ T-cell concentration in blood	*	-	u
$N_8^{LN}(0)$	LnN8	count	Mtb-specific naive CD8$^+$ T-cell count in LN	5515.3085	7010.3557	u
$P_8^{LN}(0)$	LnP8	count	Mtb-specific precursor CD8$^+$ T-cell count in LN	0	0	u
$E_8^{LN}(0)$	LnE8	count	Mtb-specific effector CD8$^+$ T-cell count in LN	0	0	u
$CM_8^{LN}(0)$	LnCM8	count	Mtb-specific central memory CD8$^+$ T-cell count in LN	0	0	u
$EM_8^{LN}(0)$	LnEM8	count	Mtb-specific effector memory CD8$^+$ T-cell count in LN	0	0	u
$N_8^{B}(0)$	BlN8	pg/mL	Mtb-specific naive CD8$^+$ T-cell concentration in blood	*	-	u
$E_8^{B}(0)$	BlE8	pg/mL	Mtb-specific effector CD8$^+$ T-cell concentration in blood	*	-	u
$CM_8^{B}(0)$	BlCM8	pg/mL	Mtb-specific central memory CD8$^+$ T-cell concentration in blood	*	-	u
$EM_8^{B}(0)$	BlEM8	pg/mL	Mtb-specific effector memory CD8$^+$ T-cell concentration in blood	*	-	u
$N_{Non,4}^{LN}(0)$	LnN4Non	count	Nonspecific naive CD4$^+$ T-cell count in LN	28970960.05181	68987195.6821	u
$CM_{Non,4}^{LN}(0)$	LnCM4Non	count	Nonspecific central memory CD4$^+$ T-cell count in LN	13572957.9382	32571421.8047	u
$N_{Non,4}^{B}(0)$	BlN4Non	pg/mL	Nonspecific naive CD4$^+$ T-cell concentration in blood	402.3744	958.1554	u
$E_{Non,4}^{B}(0)$	BlE4Non	pg/mL	Nonspecific effector CD4$^+$ T-cell concentration in blood	76.5979	328.5413	u
$CM_{Non,4}^{B}(0)$	BlCM4Non	pg/mL	Nonspecific central memory CD4$^+$ T-cell concentration in blood	188.5133	452.3808	u
$EM_{Non,4}^{B}(0)$	BlEM4Non	pg/mL	Nonspecific effector memory CD4$^+$ T-cell concentration in blood	160.1049	426.2429	u
$N_{Non,8}^{LN}(0)$	LnN8Non	count	Nonspecific naive CD8$^+$ T-cell count in LN	55147570.5476	70096547.5890	u

Table SB8: **Lymph node and blood initial conditions.** Notations are as in table SB5. *We assume that at $t = 0$, blood and lymph node concentrations are at equilibrium, so blood concentrations are calculated via their lymph-node starting count via the conversion factor α and HostLNs.

Initial condition	Non-Symbolic	Unit	LN/Blood initial condition description	Lower range	Upper range	Distribution
$CM^{LN}_{Non,8}(0)$	LnCM8Non	count	Nonspecific central memory $CD8^+$ T-cell count in LN	8169880.9373	30928893.4342	u
$N^{B}_{Non,8}(0)$	BlN8Non	pg/mL	Nonspecific naive $CD8^+$ T-cell concentration in blood	765.9384	973.5631	u
$E^{B}_{Non,8}(0)$	BlE8Non	pg/mL	Nonspecific effector $CD8^+$ T-cell concentration in blood	50.2516	346.5122	u
$CM^{B}_{Non,8}(0)$	BlCM8Non	pg/mL	Nonspecific central memory $CD8^+$ T-cell concentration in blood	113.4705	429.5679	u
$EM^{B}_{Non,8}(0)$	BlEM8Non	pg/mL	Nonspecific effector memory $CD8^+$ T-cell concentration in blood	160.3165	305.5719	u

Table SB9: **Pharmacokinetic parameter ranges.** Notations are as in table SB5. Note that all initial conditions are set to 0, because no drug is present in the patient prior to dosing. We uniformly sampled all host-scale PK parameters and host-baseline values of granuloma-scale parameters. Finally, note that we allow for distinct values of V_L, V_{PT}, and V_P within each host. This is because each drug's PK model is calibrated to a distinct datasets to replicate those experimental trajectories. This is also why we do not include PK parameters in the granuloma and host-scale sensitivity analyses, as this variation is best considered as a form of noise in the model.

Parameter	Non-symbolic	Units	PK parameter description	Scale	Drug	Lower range	Upper range	Distribution
Q_a	QA	$\frac{m}{h}$	Permeability of drug from lung into viable cellular area	Granuloma	INH	1.5	2	u
					RIF	1	1.2	u
					PZA	1	1.2	u
					EMB	0.05	0.09	u
					BDQ	0.5	5	u
					PTM	0.1	0.14	u
					LZD	0.5	0.9	u
					MXF	0.05	0.09	u
P_A	PA	-	Drug partition coefficient between lung and granuloma	Granuloma	INH	0.95	1	u
					RIF	0.4	1.1	u
					PZA	0.4	1.1	u
					EMB	0.8	1.1	u
					BDQ	0.9	10	u
					PTM	2	3	u
					LZD	0.8	1.0	u
					MXF	0.8	1.1	u

Table SB9: **Pharmacokinetic parameter ranges.** Notations are as in table SB5. Note that all initial conditions are set to 0, because no drug is present in the patient prior to dosing. We uniformly sampled all host-scale PK parameters and host-baseline values of granuloma-scale parameters. Finally, note that we allow for distinct values of V_L, V_{PT}, and V_P within each host. This is because each drug's PK model is calibrated to a distinct datasets to replicate those experimental trajectories. This is also why we do not include PK parameters in the granuloma and host-scale sensitivity analyses, as this variation is best considered as a form of noise in the model.

Parameter	Non-symbolic	Units	PK parameter description	Scale	Drug	Lower range	Upper range	Distribution
Q_c	QC	$\frac{L}{h}$	Permeability of drug from viable cellular area into caseum	Granuloma	INH	0.08	0.1	u
					RIF	0.8	1	u
					PZA	0.8	1	u
					EMB	0.8	1	u
					BDQ	0.08	0.1	u
					PTM	0.8	1	u
					LZD	0.8	1	u
					MXF	0.08	0.1	u
P_C	PC	-	Drug partition coefficient between viable cellular area and caseum	Granuloma	INH	0.8	0.8	u
					RIF	0.4	1	u
					PZA	0.5	0.8	u
					EMB	0.04	0.8	u
					BDQ	0.012	0.05	u
					PTM	1	1	-
					LZD	0.6	0.8	u
					MXF	0.2	0.55	u
β	beta	-	Drug penetration exponentiation base	Granuloma	INH	0.999	1	u
					RIF	0.0493	0.0533	u
					PZA	0.999	1	u
					EMB	1	1	-
					BDQ	.01	.01	-
					PTM	.0511	.0951	u
					LZD	0.227	0.329	u
					MXF	0.098	0.172	u

Table SB9: **Pharmacokinetic parameter ranges.** Notations are as in table SB5. Note that all initial conditions are set to 0, because no drug is present in the patient prior to dosing. We uniformly sampled all host-scale PK parameters and host-baseline values of granuloma-scale parameters. Finally, note that we allow for distinct values of V_L, V_{PT}, and V_P within each host. This is because each drug's PK model is calibrated to a distinct datasets to replicate those experimental trajectories. This is also why we do not include PK parameters in the granuloma and host-scale sensitivity analyses, as this variation is best considered as a form of noise in the model.

Parameter	Non-symbolic	Units	PK parameter description	Scale	Drug	Lower range	Upper range	Distribution
μ_C	muC	$\frac{1}{h \cdot kg}$	Drug degradation and clearance or binding within caseum	Granuloma	INH	0.8	25	u
					RIF	0.08	0.15	u
					PZA	0.08	0.15	u
					EMB	0.01	0.012	u
					BDQ	0.1	0.12	u
					PTM	0.32	0.8	u
					LZD	0.61	3	u
					MXF	0.01	0.05	u
k_d	kd	$\frac{1}{h}$	Transport rate from drug transit compartments	Host	INH	0.5	6	l
					RIF	0.2	0.4	l
					PZA	0.55	0.75	l
					EMB	1	2.75	l
					BDQ	0.03	0.1	l
					PTM	0.35	1.2	l
					LZD	0.48	1.3	l
					MXF	0.025	3.9	l
-	Transit	-	Number of drug transit compartments	Host	INH	2	2	-
					RIF	2	2	-
					PZA	1	1	-
					EMB	1	1	-
					BDQ	1	1	-
					PTM	2	2	-
					LZD	1	1	-
					MXF	1	1	-

Table SB9: **Pharmacokinetic parameter ranges.** Notations are as in table SB5. Note that all initial conditions are set to 0, because no drug is present in the patient prior to dosing. We uniformly sampled all host-scale PK parameters and host-baseline values of granuloma-scale parameters. Finally, note that we allow for distinct values of V_L, V_{PT}, and V_P within each host. This is because each drug's PK model is calibrated to a distinct datasets to replicate those experimental trajectories. This is also why we do not include PK parameters in the granuloma and host-scale sensitivity analyses, as this variation is best considered as a form of noise in the model.

Parameter	Non-symbolic	Units	PK parameter description	Scale	Drug	Lower range	Upper range	Distribution
Q	Q	$\frac{L}{h \cdot kg}$	Permeability of drug into peripheral tissue	Host	INH	0.2	7	u
					RIF	1.7	5	u
					PZA	0.1	0.7	u
					EMB	0.04	0.9	u
					BDQ	0.2	0.8	u
					PTM	3	4	u
					LZD	10.36	45	u
					MXF	0.05	40	u
μ_D	muD	$\frac{L}{h \cdot kg}$	Decay rate of drug within lung tissue	Host	INH	0.08	2.5	u
					RIF	0.08	0.15	u
					PZA	0.08	0.15	u
					EMB	0.01	0.012	u
					BDQ	0.01	0.012	u
					PTM	0.32	0.8	u
					LZD	0.61	3	u
					MXF	0.01	0.05	u
μ_{DP}	muDP	$\frac{L}{h \cdot kg}$	Decay rate of drug within peripheral tissue	Host	INH	0.08	2.5	u
					RIF	0.08	0.15	u
					PZA	0.01	0.05	u
					EMB	1	2.5	u
					BDQ	1.5	4	u
					PTM	0.2	0.8	u
					LZD	0.04	0.91	u
					MXF	0.05	0.6	u

Table SB9: **Pharmacokinetic parameter ranges.** Notations are as in table SB5. Note that all initial conditions are set to 0, because no drug is present in the patient prior to dosing. We uniformly sampled all host-scale PK parameters and host-baseline values of granuloma-scale parameters. Finally, note that we allow for distinct values of V_L, V_{PT}, and V_P within each host. This is because each drug's PK model is calibrated to a distinct datasets to replicate those experimental trajectories. This is also why we do not include PK parameters in the granuloma and host-scale sensitivity analyses, as this variation is best considered as a form of noise in the model.

Parameter	Non-symbolic	Units	PK parameter description	Scale	Drug	Lower range	Upper range	Distribution
V_P	VP	$\frac{L}{kg}$	Volume of Plasma distribution	Host	INH	0.5	3.0	u
					RIF	0.08	0.6	u
					PZA	0.25	0.75	u
					EMB	2.7	4.5	u
					BDQ	0.05	5	u
					PTM	1	4	u
					LZD	0.16	1.71	u
					MXF	0.65	2.5	u
P_L	PL	-	Drug partition coefficient between lung and plasma	Host	INH	1	1	-
					RIF	0.07	1	u
					PZA	1	1	-
					EMB	12	16	u
					BDQ	10	500	u
					PTM	1.2	20	u
					LZD	1.1	2	u
					MXF	1	9	u
V_{PT}	VPT	$\frac{L}{kg}$	Volume of peripheral tissue distribution	Host	INH	25	40	u
					RIF	0.05	0.2	u
					PZA	0.01	0.05	u
					EMB	0.08	1	u
					BDQ	0.1	50	u
					PTM	3.5	6	u
					LZD	0.2	1.9	u
					MXF	0.03	0.4	u

Table SB9: **Pharmacokinetic parameter ranges.** Notations are as in table SB5. Note that all initial conditions are set to 0, because no drug is present in the patient prior to dosing. We uniformly sampled all host-scale PK parameters and host-baseline values of granuloma-scale parameters. Finally, note that we allow for distinct values of V_L, V_{PT}, and V_P within each host. This is because each drug's PK model is calibrated to a distinct datasets to replicate those experimental trajectories. This is also why we do not include PK parameters in the granuloma and host-scale sensitivity analyses, as this variation is best considered as a form of noise in the model.

Parameter	Non-symbolic	Units	PK parameter description	Scale	Drug	Lower range	Upper range	Distribution
V_L	VL	$\frac{L}{kg}$	Volume of lung distribution	Host	INH	0.08	0.08	-
					RIF	0.08	0.6	u
					PZA	0.08	0.6	u
					EMB	1	6	u
					BDQ	1	6	u
					PTM	1	6	u
					LZD	1	6	u
					MXF	0.05	0.6	u
